# A study of deregulated MMR pathways and anticancer potential of curcuma derivatives using computational approach

**DOI:** 10.1038/s41598-021-89282-5

**Published:** 2021-05-12

**Authors:** Priyanjali Bhattacharya, Trupti N. Patel

**Affiliations:** Department of Integrative Biology, Vellore Institute of Technology, Vellore, India

**Keywords:** Computational biology and bioinformatics, Cancer, Haematological cancer

## Abstract

Plant derived products have steadily gained momentum in treatment of cancer over the past decades. Curcuma and its derivatives, in particular, have diverse medicinal properties including anticancer potential with proven safety as supported by numerous in vivo and in vitro studies. A defective Mis-Match Repair (MMR) is implicated in solid tumors but its role in haematologic malignancies is not keenly studied and the current literature suggests that it is limited. Nonetheless, there are multiple pathways interjecting the mismatch repair proteins in haematologic cancers that may have a direct or indirect implication in progression of the disease. Here, through computational analysis, we target proteins that are involved in rewiring of multiple signaling cascades via altered expression in cancer using various curcuma derivatives (*Curcuma longa L.* and *Curcuma caesia Roxb.*) which in turn, profoundly controls MMR protein function. These biomolecules were screened to identify their efficacy on selected targets (in blood-related cancers); aberrations of which adversely impacted mismatch repair machinery. The study revealed that of the 536 compounds screened, six of them may have the potential to regulate the expression of identified targets and thus revive the MMR function preventing genomic instability. These results reveal that there may be potential plant derived biomolecules that may have anticancer properties against the tumors driven by deregulated MMR-pathways.

## Introduction

Cancer is a multistep process that involves overexpression of oncogenes and silencing of tumor suppressors through mutations or epimutations. Mutations in cancer, lead to uncontrolled cellular proliferation and evasion of apoptosis as directed by ‘driver mutations’ in solid tumors and haematologic malignancies^1^. Loss of function of the TSGs (Tumour Suppressor Genes) and accompanied increase in the oncogenic expression rewire the signaling cascades that promote ‘malignant phenotype’. In order to provide an effective treatment that deals with such perturbations, the ‘functional nodes of malformed network’ needs to be identified and restrained^1,2^.

The genomic DNA is under continuous stress due to endogenous and/ or exogenous toxic insults, for which multiple DNA repair mechanisms exists viz., excision repair (base excision repair-BER; nucleotide excision repair-NER), mismatch repair (MMR), and double stranded break repair (homologues recombination- HR and non-homologous end joining- NHEJ)^3^. These protect the damage to genetic material and formation of abnormal cells that may become immortalized instead of going through senescence. The Mis-Match Repair (MMR) system is one such mechanism involving 9 genes- MSH2-6, MLH1 and 3, and PMS1-2 that form heterodimeric protein complexes which help in recognition and repair of mis-incorporations and mis-alignments^3,4^. Approximately 15% of all primary tumors exhibit MMR deficiency, which can directly impact the DNA leading to malignant transformation^4^. Stoklosa et al. (2008) found that increased level of reactive oxygen species (ROS) resulted in accumulation of DNA lesions in BCR-ABL positive CML (Chronic Myeloid Leukaemia) cells which was associated with inhibition of MMR functions leading to increased genomic instability^5^. Mutations in MMR genes viz. MSH2 and MLH1 and promoter hypermethylation of MLH1 correlates with loss of function of mismatch repair and AML (Acute Myeloid Leukaemia) pathogenesis^6^. Besides this, a series of research conducted between 1994 and 2011 suggested mutations in one or both copies of mismatch repair genes results in myeloid and/ or lymphoid malignancies, supporting the role of MMR deficiency in malignant transformation^7–10^. Though microsatellite instability in MMR genes are not directly implicated in haematologic malignancies, their role in causation and progression of blood-related cancers cannot be completely repudiated.

Plant derived products- phytochemicals; have been used for medicinal purposes, traditionally across the Asian subcontinent. Through extensive research and pharmaceutical success, currently four major groups of plant products viz., vinca alkaloids, the epipodophyllotoxins, the taxanes, and the camptothecin derivatives have revolutionized anticancer treatments. Due to their geographical, chemical and biological diversity, with an easy to access, researchers across the globe are working towards development of efficient anticancer drugs from various plants^11^. Multiple variety of turmeric is cultivated around the world especially in the tropical and subtropical regions of India, Southeast Asia, and Indonesia. Turmeric belongs to Zingiberaceae family and carries curcumin, a polyphenol, in its rhizomes. Besides being used as flavouring and colouring agent, turmeric has wide range of medicinal applications against microbial infections, inflammations, and neurological diseases, and most importantly in cancer prevention and cure^12^. The anticancer mechanism of curcumin involves inhibition of proliferation, invasion, and metastasis, and induction of apoptosis which works via modulating various signaling pathways^13,14^.

*Here, we select targets for turmeric compounds that are implicated in cancers, especially leukaemia and lymphomas, which directly or indirectly impact the function of MMR proteins. We use computational tools to investigate the potential of biomolecules from Curcuma longa L. and Curcuma caesia Roxb. (Black turmeric) against abnormal functional nodes that in turn regulate the expression of MMR proteins in cancer.*

## Results

### Analysis of molecular properties, biological activity, ADME-tox, drug and lead-likeness of curcuma compounds

A total of 536 curcuma compounds (*C. longa L*., and *C. caesia Roxb*.) were screened using Molinspiration, preADMET, pkCSM, and SwissADME tools to determine the physicochemical properties, biological activities, ADME-Tox (Absorption-Distribution-Metabolism-Excretion and Toxicity), and drug/lead-likeness.

From the initial dataset, through exclusion, a total of 30 compounds were selected based on their molecular, biological, pharmacokinetic and drug-like properties (Tables 1, 2, 3, 4).

**Table 1:** Molinspiration results depicting Molecular properties of ALL the 30 compounds filtered from 536 compounds.

Compound ID	miLogP	TPSA	Natoms	MW	nON	nOHNH	nrotB	Volume
EF31 (CL)	0.66	54.88	21	277.33	4	1	2	256.12
UBS109	1.25	46.09	22	291.35	4	0	2	273.06
2	2.90	66.76	23	308.33	4	2	2	277.05
5	1.69	**115.05**	25	340.33	6	4	6	297.13
8	1.73	66.76	17	234.25	4	2	4	216.00
9	**6.52**	65.00	31	432.60	5	1	9	441.07
10	**5.34**	89.14	31	426.51	6	2	9	404.63
64	1.34	63.16	25	331.38	5	0	3	303.22
71	1.80	65.11	23	306.32	5	0	4	271.58
α-pinene	3.54	**0.00**	10	**136.24**	0	0	0	151.81
acsjm5	2.97	63.58	21	284.31	4	1	5	260.96
acsjm6	2.52	63.58	19	256.26	4	1	5	227.84
Bisacurone	1.87	57.53	18	252.35	3	2	4	258.48
Curcumol	4.38	29.46	17	236.35	2	1	1	242.05
1d	2.54	15.79	10	**131.18**	1	1	0	129.58
242	2.46	29.96	16	209.25	2	0	3	197.70
E21CH	2.24	66.76	18	248.28	4	2	5	232.81
46	**5.83**	79.90	31	424.54	5	2	8	412.21
155	1.48	42.85	18	236.27	3	0	4	220.96
180	0.68	74.60	16	220.22	4	2	4	198.83
181	0.99	63.60	17	234.25	4	1	5	216.35
182	1.17	54.37	15	204.22	3	1	4	190.81
184	0.70	83.83	18	250.25	5	2	5	224.37
185	3.38	72.84	25	348.44	5	1	8	341.15
3(CCR)	2.79	43.69	20	281.44	3	2	6	298.49
91	2.25	57.53	18	252.35	3	2	0	253.50
84	2.88	34.14	17	234.34	2	0	3	239.94
88	2.28	63.60	19	262.31	4	1	0	240.16
95	2.54	57.53	19	264.37	3	2	0	263.52
103	2.88	34.14	17	234.34	2	0	3	239.94

*CL Curcuma longa L.*, *CCR Curcuma caesia Roxb*., *TPSA* topological polar surface area, *MW* molecular weight, *nOHNH* H-bond donor, *nON* H-bond acceptor.

Bold compounds with minimum and maximum TPSA and compounds that did not fall in the expected range for miLogP and molecular weight parameters.

**Table 2:** Molinspiration results depicting Bioactivity of ALL the 30 compounds filtered from 536 compounds.

Compound ID	GPCR ligand	Ion channel modulator	Kinase inhibitor	Nuclear receptor ligand	Protease inhibitor	Enzyme inhibitor
EF31 (CL)	0.11	− 0.09	− 0.19	− 0.19	0.12	0.23
UBS109	0.15	− 0.08	− 0.04	− 0.08	0.08	0.17
2	− 0.12	− 0.12	− 0.24	0.14	0.00	0.09
5	− 0.01	− 0.15	− 0.26	0.20	− 0.07	0.13
8	− 0.53	− 0.51	− 0.64	− 0.40	− 0.58	0.03
9	− 0.23	− 0.23	− 0.37	0.15	− 0.21	0.01
10	− 0.09	− 0.39	− 0.25	0.04	− 0.03	0.23
64	− **0.09**	− **0.23**	− **0.21**	− **0.24**	− **0.07**	− **0.04**
71	− 0.05	− 0.65	− 0.23	− 0.20	− 0.47	0.12
α-pinene	− **0.48**	− **0.43**	− **1.50**	− **0.62**	− **0.85**	− **0.34**
acsjm5	− **0.38**	− **0.89**	− **0.47**	− **0.25**	− **0.34**	− **0.07**
acsjm6	− **0.48**	− **0.91**	− **0.62**	− **0.43**	− **0.54**	− **0.05**
Bisacurone	− 0.07	0.07	− 0.97	0.82	0.14	0.69
Curcumol	− 0.40	0.13	− 0.65	0.08	− 0.25	0.25
1d	− **0.44**	− **0.08**	− **0.41**	− **1.05**	− **1.25**	− **0.20**
242	− 0.17	− 0.04	− 0.37	− 0.66	− 0.46	0.09
E21CH	− 0.33	− 0.40	− 0.59	− 0.11	− 0.42	0.18
46	− 0.06	− 0.51	− 0.33	0.17	− 0.10	0.18
155	− 0.03	− 0.14	− 0.10	− 0.30	− 0.15	0.23
180	− 0.44	− 0.29	− 0.97	− 0.11	− 0.66	0.12
181	− 0.42	− 0.35	− 0.88	− 0.14	− 0.67	0.09
182	− 0.52	− 0.30	− 1.07	− 0.14	− 0.73	0.09
184	− 0.36	− 0.31	− 0.73	− 0.10	− 0.56	0.17
185	0.00	− 0.10	− 0.39	0.27	− 0.02	0.29
3 (CCR)	0.52	0.53	0.05	0.26	0.33	0.37
91	− 0.42	− 0.29	− 1.01	0.24	− 0.39	0.22
84	− **0.71**	− **0.59**	− **1.23**	− **0.42**	− **0.65**	− **0.22**
88	− 0.42	− 0.13	− 0.89	0.09	− 0.32	0.32
95	− 0.37	− 0.06	− 0.76	0.53	− 0.31	0.24
103	− **0.71**	− **0.59**	− **1.23**	− **0.42**	− **0.65**	− **0.22**

*CL Curcuma longa L.*, *CCR Curcuma caesia Roxb.*

Bold compounds that were disqualified.

**Table 3:** ADME-Tox and Druglikeness properties of 30 compounds as predicted by preADMET and pkCSM.

Compound ID	Absorption			Distribution		Metabolism				Excretion		Druglikeliness					Toxicity		
	HIA	Caco2	MDCK	PPB	BBB	CYP2C19	CYP2C9	CYP3A4	CYP2D6	log(CL_tot_)	Renal OCT2	CMC	Lead	MDDR	Lipinski	WDI	Ames	Mice	Rat
EF31 (CL)	95.99	44.79	32.46	54.10	0.08	N	N	N	N	0.816	N	*	**	#	##	*#	M	–	–
UBS109	97.53	55.56	42.13	58.73	0.53	N	N	N	N	0.682	N	*	**	#	##	*#	M	–	–
2	93.69	19.99	**7.10**	85.51	0.90	**I**	**I**	**I**	N	0.119	N	*	**	#	##	*#	M	–	–
5	83.96	18.89	53.37	**95.62**	0.47	**I**	**I**	**I**	N	**− 0.004**	N	*	**	#	##	*#	N	–	+
8	91.27	17.36	94.11	75.94	0.14	**I**	**I**	N	N	0.276	N	*	**	#	##	*#	**M**	** + **	** + **
9	95.65	46.53	170.46	73.65	0.02	**I**	**I**	**I**	N	**1.779**	N	*	**	#	##	*#	M	–	+
10	90.14	13.96	185.00	**90.60**	0.18	**I**	**I**	**I**	N	0.297	N	*	**	#	##	*#	M	+	–
64	97.90	30.12	**13.87**	80.66	0.06	N	**I**	N	N	0.363	N	*	**	#	##	*#	M	–	+
71	98.13	33.06	**12.49**	**92.20**	0.01	**I**	**I**	**I**	N	0.776	**Y**	*	**	#	##	*#	**M**	** + **	** + **
α-pinene	100.00	23.63	304.81	**100.00**	**5.53**	N	**I**	N	N	0.043	N	*	**	#	##	*#	M	–	+
acsjm5	95.92	20.84	64.62	**100.00**	0.06	**I**	**I**	**I**	N	0.772	N	*	**	#	##	*#	**M**	** + **	** + **
acsjm6	95.85	13.84	47.64	**100.00**	0.09	**I**	**I**	**I**	N	0.745	N	*	**	#	##	*#	**M**	** + **	** + **
Bisacurone	90.98	24.61	321.05	88.34	0.45	**I**	**I**	N	N	1.382	N	*	**	#	##	*#	N	–	–
Curcumol	97.00	53.10	63.27	**100.00**	**4.21**	N	**I**	**I**	N	0.975	N	*	**	#	##	*#	M	–	+
1d	100.00	32.56	73.05	**92.93**	**9.74**	**I**	**I**	**I**	N	0.481	**Y**	*	**	#	##	*#	M	+	–
242	100.00	53.78	65.66	89.79	**2.51**	**I**	**I**	N	N	0.213	N	*	**	#	##	*#	M	+	–
E21CH	91.63	17.28	72.08	**91.29**	0.26	**I**	**I**	N	N	0.365	N	*	**	#	##	*#	M	–	+
46	93.88	30.53	79.12	**90.82**	0.13	**I**	**I**	**I**	N	0.327	N	*	**	#	##	*#	M	+	–
155	97.84	55.30	62.77	**92.89**	0.20	**I**	**I**	N	N	0.185	N	*	**	#	##	*#	**M**	** + **	** + **
180	89.50	15.43	42.32	68.24	0.05	**I**	**I**	**I**	N	0.140	N	*	**	#	##	*#	M	–	+
181	94.78	14.87	104.17	66.27	0.01	**I**	**I**	N	N	0.199	N	*	**	#	##	*#	M	–	+
182	94.80	20.88	37.90	76.25	0.03	**I**	**I**	N	N	0.156	N	*	**	#	##	*#	M	–	+
184	88.38	6.90	76.21	61.05	0.03	**I**	**I**	**I**	N	0.138	N	*	**	#	##	*#	M	–	+
185	94.32	8.32	152.16	60.13	0.01	**I**	**I**	**I**	N	1.663	N	*	**	#	##	*#	M	–	+
3 (CCR)	91.45	38.05	56.08	85.74	**3.21**	N	N	N	N	0.938	N	*	**	#	##	*#	N	–	–
91	90.44	19.40	56.30	61.89	1.48	N	**I**	N	N	1.064	N	*	**	#	##	*#	N	–	–
84	99.52	29.45	176.01	**100.00**	0.88	N	**I**	**I**	N	1.063	**Y**	*	**	#	##	*#	N	–	+
88	94.69	20.78	45.34	87.97	0.61	N	**I**	**I**	N	1.074	N	*	**	#	##	*#	N	–	+
95	91.37	21.12	58.94	72.92	**2.33**	N	**I**	**I**	N	1.156	N	*	**	#	##	*#	N	–	+
103	99.52	29.45	176.01	**100.00**	0.88	N	**I**	**I**	N	1.063	N	*	**	#	##	*#	N	–	+

*CL Curcuma longa L., CCR Curcuma caesia Roxb*., *HIA* human intestinal absorption, *MDCK* madin-darby canine kidney, *PPB* plasma protein binding, *BBB* blood brain barrier, *CYP* cytochrome P450, *N* noninhibitor, *I* inhibitor, *OCT2* organic cation transporter 2, *N* no, *Y* yes; CMC—*—satisfied, Lead—**—Suitable if binding affinity is > 0.1 µM, MDDR—#—MID-structure ranges only, Lipinski—##—Suitable, WDI—*#—90% cut off; Ames—M- Mutagen, N-Non mutagen; Carcinogenicity—+ : Positive, −: Negative; Bold- compounds with high and/or low ADME scores, CYP inhibition properties, renal OCT2 substrate property, violating CMC/WDI rules, and showing mutagenecity and carcinogenicity.

**Table 4:** Additional Drug (Ghose-Veber-Egan-Muegge) and Lead-like properties of 30 compounds as predicted by SwissADME.

Compound ID	Druglikeliness				Leadlikeness
	Ghose	Veber	Egan	Muegge	
EF31 (CL)	Y	Y	Y	Y	Y
UBS109	Y	Y	Y	Y	Y
2	Y	Y	Y	Y	Y
5	Y	Y	Y	Y	Y
8	Y	Y	Y	Y	**N**
9	Y	Y	Y	Y	Y
10	Y	Y	Y	Y	Y
64	Y	Y	Y	Y	Y
71	Y	Y	Y	Y	Y
α-pinene	**V**	Y	Y	**V**	**N**
acsjm5	Y	Y	Y	Y	**N**
acsjm6	Y	Y	Y	Y	Y
Bisacurone	Y	Y	Y	Y	Y
Curcumol	Y	Y	Y	Y	**N**
1d	**V**	Y	Y	**V**	**N**
242	Y	Y	Y	Y	**N**
E21CH	Y	Y	Y	Y	**N**
46	Y	Y	Y	Y	Y
155	Y	Y	Y	Y	Y
180	Y	Y	Y	Y	**N**
181	Y	Y	Y	Y	**N**
182	Y	Y	Y	Y	**N**
184	Y	Y	Y	Y	Y
185	Y	Y	Y	Y	Y
3 (CCR)	Y	Y	Y	Y	Y
91	Y	Y	Y	Y	Y
84	Y	Y	Y	Y	**N**
88	Y	Y	Y	Y	Y
95	Y	Y	Y	Y	Y
103	Y	Y	Y	Y	Y

*CL Curcuma longa L.*, *CCR Curcuma caesia Roxb.*, *Y* yes, *N* no, *V* violated.

Bold compounds that did not satisfy drug/lead-like properties.

Table 1 demonstrates the molecular properties of all the 30 compounds. The octanol/water partition coefficient indicated by **logP** showed 27 curcuma compounds were in the range of 0.66–3.38; except 3 compounds (Compound- 9, 10, and 46 of *C. Longa L.*), for which the value of logP was > 5 thus not satisfying the Lipinski rule (Rule of five: −2 ≤ logP ≤ 5). Hence the other compounds, falling in acceptable range were lipophilic, which is a major descriptor to understand the absorption, distribution, transport, and impact of biomolecules in physiological systems. The **molecular weight** of 28 compounds was found to be in an acceptable range viz. 200 ≤ MW ≤ 500 (Rule of five). The compounds with low molecular weight tend to absorb well, and hence are more suited as pharmaceutical products. However, 2 curcuma compounds viz. α-pinene, and Compound 1d (*C. longa L.*) violated this parameter (< 200 g/mol) due to very low molecular weight. Though such low molecular weight compounds, also known as ‘fragments’, are currently being screened for druggability, here we have dismissed them. The number of **H-bond donors** (≤ 5) and **acceptors** (≤ 10) for all 30 compounds were ranged between 0–4 and 0–6 respectively, which in turn satisfied the Lipinski’s rule of five. The **topological polar surface area** (TPSA < 140 Å), which defines the ‘relative propensity for polar interactions’ of target proteins with ligands was least and maximum for- α-pinene (0 Å), and Compound 5 (115.05 Å) of *C. longa L.* respectively covering all the compounds here. The minimum and maximum number of **rotatable bonds** was found to be 0 and 9 respectively for all the 30 compounds, depicting molecular flexibility of these compounds allowing possible favourable interaction with proteins.

Table 2 demonstrates the bioactivity of 30 compounds in terms of GPCR ligand, ion channel modulator, kinase-protein-enzyme inhibitors, and nuclear receptor ligand. The *C. longa L.* Compounds- 64, α-pinene, acsjm5, acsjm6, 1d, and *C. caesia Roxb*. Compounds 84 and 103 didn’t show any promising bioactivity, hence were disqualified from the study.

The ADME-Tox properties and druglikeness of 30 compounds as predicted by preADMET and pkCSM are enlisted in Table 3.The **absorption (A)** of the curcuma compounds was screened against HIA (Human Intestinal Absorption), Caco-2, and MDCK (Madin-Darby Canine Kidney) parameters. The HIA of 30 compounds was in the range of ~ 83%–100%; indicating the compounds to be well absorbed. All the 30 compounds were moderately permeable with respect to Caco-2 (4 < Caco-2 < 70) and MDCK (25 < MDCK < 500) cell permeability. However, three compounds- 2, 64, and 71 (*C. longa L.*) were less permeable in terms of MDCK. The **distribution (D)** parameter evaluated the level of Plasma Protein Binding (PPB) and Blood Brain Barrier (BBB) penetration. Total thirteen compounds viz. 5, 10, 71, α-pinene, acsjm5, acsjm6, curcumol, 1d, E21CH, 46, 155 of *C. longa L.*, and Compounds 84, and 103 of *C. caesia Roxb*. showed strong plasma protein binding affinity qualifying them for further analysis. In BBB category, 6 compounds viz. α-pinene, curcumol, 1d and 242 of *C. longa L.*, and Compounds 3, and 95 of *C. caesia Roxb*. exhibited high absorption to CNS (Central Nervous System), thus indicating their potential to work across the blood brain barrier. The **metabolism (M)** parameter evaluated the cytochrome P450 inhibitors and non-inhibitors. A compound that is a non-inhibitor serves as less toxic drug. Twenty two *C. longa L.* Compounds viz., 2, 5, 8, 9, 10, 64, 71, α-pinene, acsjm5, acsjm6, Bisacurone, Curcumol, 1d, 242, E21CH, 46, 155, 180, 181, 182, 184, and 185 while five compound from *C. caesia Roxb*. Compounds- 103, 91, 88, 95 and 84 were selective inhibitors of cytochrome P450 enzymes—CYP4502C9, 2C19, and 3A4. The **excretion (E)** parameter calculated total clearance [log(CL_tot_)] and Renal OCT2 (Organic Cation Transporter 2) properties of curcuma compounds. The log(CL_tot_) value was minimum for Compound 5 (−0.004) and maximum for Compound 9 (1.779) of *C. longa L*. Besides this, compounds 71, 1d (*C. longa L*), and 84 (*C. caesia Roxb.*) were found to be a substrate for Renal OCT2. The compound with minimum clearance and substrate to Renal OCT2 (reducing Renal OCT2 dependent clearance) may show have increased systemic presence and may incur toxicity.

The ‘**druglikeness**’ was calculated using CMC/MDDR/Lead-like rules, World Drug Index (WDI), Lipinski’s ‘rule of five’, and the Ghose, Veber, Egan, and Muegge methods (Tables 3, 4). The CMC, MDDR and WDI enlist compounds that are under development, tested in humans or launched (sources: patent, literature, conference proceedings). The qualifying criteria of **CMC-like rule** are- logP: 0.4–5.6; molecular weight: 160–480; number of atoms: 20–70; and molar refractivity: 40–130. The **MDDR-like rule** discriminates a compound as drug-like, non-drug like and mid-structure depending upon the number of rings, rigid bonds and rotatable bonds. The cut off criteria for **Lead like rule** are divided into three categories- high affinity leads [affinity < < 0.1 µM; molecular weight >>   350; ClogP < 3]; lead like leads [affinity > 0.1 µM; molecular weight < 350; ClogP < 3], and drug like leads [affinity > 0.1 µM; molecular weight > 350; ClogP > 3]. The compounds with molecular properties within 90% range are categorized into **WDI**. All the 30 compounds were in the mid-structure ranges which otherwise qualified the drug-like properties using CMC (except 3 compounds- α-pinene, 1d, 46 of *C. longa L.*), and WDI (except 8 compounds- 9, 10, acsjm5, acsjm6, 1d, 46, 184, 185 of *C. longa L.*).

In terms of toxicity, the *C. longa L.* Compounds- 8, 71, acsjm5, acsjm6, and 155 were found to be both mutagenic and carcinogenic. Alternate toxicities of other biomolecules need to be tested in vitro or in vivo.

The additional drug-like properties along with lead-likeness of 30 compounds are tabulated in Table 4. The **Ghose** method filters the compound on basis of molecular weight [160 ≤ MW ≤ 480], logP [−0.4 ≤ logp ≤ 5.6], molar refractivity [40 ≤ MR ≤ 130], and total number of atoms [20 ≤ atoms ≤ 70]. The **Veber** method considers number of rotatable bonds [rotB ≤ 10] and topological polar surface area [TPSA ≤ 140]. The **Egan** method considers logP [logP ≤ 5.88], and TPSA [≤ 13.16]. Finally, the **Muegge** method calculates- molecular weight [200 ≤ MW ≤ 600], logP [−2 ≤ logP ≤ 5], TPSA [≤ 150], number of rings [≤ 7], carbon number [> 4], number of heteroatom [> 1], rotatable bonds [≤ 15], H-bond acceptor [≤ 10], and donor [≤ 5]. Twenty eight compounds satisfied Ghose-Veber-Egan-Muegge criteria excluding α-pinene, and Compound 1d of *C. longa L.* which violated the Ghose, and Muegge rules. A good lead is a ‘molecular entity suitable for optimization’. The leads, by definition, need to be smaller in size, less hydrophobic in comparison to drug-like molecules and undergo chemical modifications to increase lipophilicity. The *C. longa L.* Compounds- 8, α-pinene, acsjm5, curcumol, 1d, 242, E21CH, 180, 181, 182, and *C. caesia Roxb*. Compound 84 did not satisfy lead like criteria [250 ≤ MW ≤ 350; logP ≤ 3.5; rotatable bonds ≤ 7].

**Figure 1:**
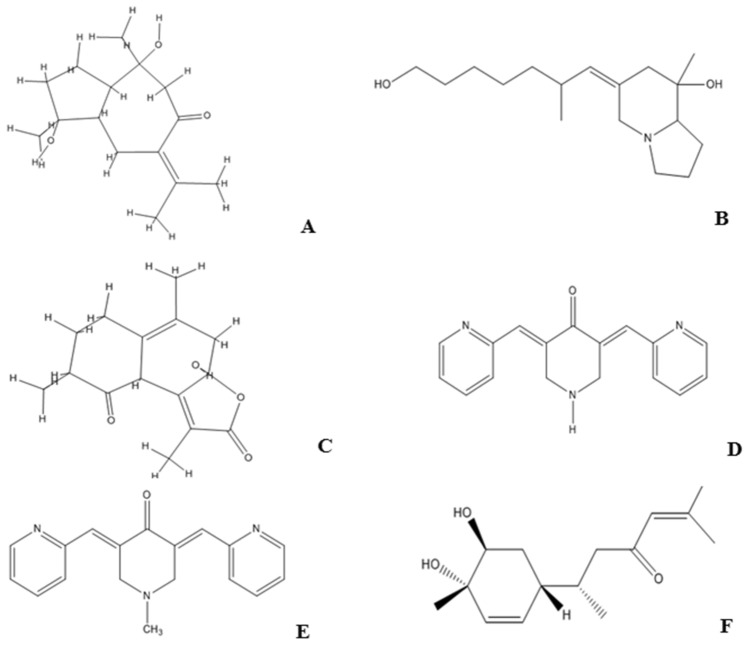
Structure of Ligands (**A**) Compound 91, (**B**) Compound 3, (**C**) Compound 88, (**D**) EF31, (**E**) UBS109, (**F**) Bisacurone.

The six curcuma compounds viz. Compounds 3, 88, 91 from *C. caesia Roxb.*, and EF31, UBS109, Bisacurone from *C. longa L.* moderately satisfied all the criteria and were further used in the study. The Fig. 1 shows the chemical structure of these 6 compounds.

The Table 5 demonstrates the chemical formula and IUPAC names of the six compounds. Amongst these, the Compounds 3, 88, and 91 are natural compounds present in *C. caesia Roxb*., and Bisacurone is a non-curcuminoid found in the oil of *C. longa L.* Earlier investigation identified these three natural compounds of black turmeric derived from hexane rhizome extract^15,16^. EF31 and UBS109, on the other, are two monocarbonyl derivatives of *C. longa L.*

**Table 5:** Chemical Formula and IUPAC Name of the six compounds.

Compound ID	Chemical formula	IUPAC NAME
91 (CCR)	C_15_H_24_O_3_	(1S,3aS,4S,8aR)-1,4-dihydroxy-1,4-dimethyl-7-(propan-2-ylidene)octahydroazulen-6(1H)-one
3 (Pumiliotoxin)	C_17_H_31_NO_2_	(Z)-6-(7-hydroxy-2-methylheptylidene)-8-methyloctahydroindolizin-8-ol
88	C_15_H_18_O_4_	(3aR,8R,9aR)-3a-hydroxy-1,5,8-trimethyl-4,7,8,9a-tetrahydronaphtho[2,1-b]furan-2,9(3aH,6H)-dione
EF31 (CL)	C_17_H_15_N_3_O	(3E,5E)-3,5-bis(pyridin-2-ylmethylene)piperidin-4-one
UBS109	C_18_H_17_N_3_O	(3E,5E)-1-methyl-3,5-bis(pyridin-2-ylmethylene)piperidin-4-one
Bisacurone	C_15_H_24_O_3_	(S)-6-((1R,4S,5S)-4,5-dihydroxy-4-methylcyclohex-2-en-1-yl)-2-methylhept-2-en-4-one

*CCR Curcuma caesia Roxb*., *CL Curcuma longa L.*

The molecular properties of these 6 compounds are tabulated in Table 6. Most of the drugs in their active form are lipophilic since they are transported through the cell membrane via diffusion and not by specialized transport systems. The **logP value** for all the six compounds ranged between 0.66 and 2.79 (− 7.0 ≤ logP ≤ 6.0), which indicated the compounds to be lipophilic in nature. The lipophilic drugs tend to absorb more and excrete less giving a higher pharmacologic half-life to such molecules. A water soluble drug molecule facilitates delivery of its active ingredient in sufficient quantity but tend to excrete at a higher rate. The **Estimated SOLubility (ESOL ≤ 6)** as predicted by logS value indicated the compounds to be poorly soluble in water. The logS for the six compounds were: −2.54 (EF31), −2.91 (UBS109), −2.32 (Bisacurone), −2.61 (Compound 3), −1.71 (Compound 88), and −2.41 (Compound 91). Though lipophilicity is a desired feature, the biomolecules could be modified to increase their solubility and reduce their CNS, hepatic or liver toxicity. The **molecular weight** of the 6 compounds ranged between 252.35 and 291.35 g/mol which, as earlier mentioned, is indicative of better absorption of the biomolecules. The numbers of **hydrogen bond donor** were- 0 (UBS109), 1 (EF31 and compound 88), and 2 (Bisacurone, Compound 3 and 91), while the numbers of **hydrogen bond acceptor** were- 3 (Compound 3, 91 and Bisacurone) and 4 (EF31, UBS109 and Compound 88) showing a decent binding potency of these drug molecule to their targets. The number of **rotatable bonds** was- 0 (Compound 88 and 91), 2 (EF31 and UBS109), 4 (Bisacurone), and 6 (Compound 3) which enabled higher flexibility of the compounds for active molecular interaction. The **topological polar surface area (TPSA)** of the six curcuma compounds was in the range between 43.69 and 63.30 Å (TPSA ≤ 140 Å) indicating greater bioavailability and better drug distribution. Compound 3 satisfied most of the molecular properties as a candidate molecule.

**Table 6:** Molecular properties of 6 curcuma compounds.

Compound ID	miLogP	TPSA	Natoms	MW	nON	nOHNH	nrotB	Volume	LogS(ESOL)
91 (CCR)	2.25	57.53	18	252.35	3	2	0	253.50	− 2.41
3	**2.79**	**43.69**	**20**	**281.44**	**3**	**2**	**6**	**298.49**	− **2.61**
88	2.28	63.60	19	262.31	4	1	0	240.16	− 1.71
EF31 (CL)	0.66	54.88	21	277.33	4	1	2	256.12	− 2.54
UBS109	1.25	46.09	22	291.35	4	0	2	273.06	− 2.91
Bisacurone	1.87	57.53	18	252.35	3	2	4	258.48	− 2.32

*CCR Curcuma caesia Roxb*., *CL Curcuma longa L.*, *miLogP* octanol/water partition coefficient, *nOHNH* H-bond donor, *nON* H-bond acceptor, *MW* molecular weight, *TPSA* topological polar surface area, *nrotB* no. of rotatable bond, *natoms* no. of atoms, *ESOL* estimated solubility.

Bold- Compound 3 having maximum volume, lipophilicity, hydrogen bond donors, and rotatable bonds.

The bioactivity scores of 6 compounds are enlisted in Table 7. The **nuclear receptors** are highly conserved transcription factors containing DNA binding and ligand binding domains while the **GPCR**s bind and respond to distinct extracellular ligands^17^. Binding of ligand allows the receptor to trigger multiple intracellular signaling cascades via specific ligand bound receptor conformation^18^. The Compounds 3, 88, 91, and Bisacurone satisfied ‘nuclear receptor ligand’ criteria, which means these compounds can possibly mediate transcriptional regulation of genes involved in numerous biological functions. Besides this, the Compound 3, EF31, and UBS109 satisfied GPCR ligand criteria. Therefore, binding of these ligands with GPCR might activate the flow of signal via modulating the downstream effectors. The natural products inhibit certain kinases, and enzymes. In our study, we found all these six compounds had **enzymatic inhibition properties** and Compound 3, in particular, showed kinase inhibition property. Similarly, overexpression of **ion channels** in pathophysiology of cancer like diseases has prompted towards discovery of potent ligands with ion channel modulators. In our study, Bisacurone (0.07), and Compound 3 (0.53) modulated the ion channels. **Protease inhibitors**, on the other, can prevent tumor progression and carcinogenesis and via blocking or altering the access to enzyme’s catalytic site^19^. We found Compound 3 (0.33), EF31 (0.12), UBS109 (0.08), and Bisacurone (0.14) having protease inhibitor activity.

**Table 7:** Molinspiration results depicting Bioactivity of 6 compounds.

Compound ID	GPCR ligand	Ion channel modulator	Kinase inhibitor	Nuclear receptor ligand	Protease inhibitor	Enzyme inhibitor
91 (CCR)	− 0.42	− 0.29	− 1.01	0.24	− 0.39	0.22
3	**0.52**	**0.53**	**0.05**	**0.26**	**0.33**	**0.37**
88	− 0.42	− 0.13	− 0.89	0.09	− 0.32	0.32
EF31 (CL)	0.11	− 0.09	− 0.19	− 0.19	0.12	0.23
UBS109	0.15	− 0.08	− 0.04	− 0.08	0.08	0.17
Bisacurone	− 0.07	0.07	− 0.97	0.82	0.14	0.69

*CCR Curcuma caesia Roxb*., *CL Curcuma longa L.*

Bold maximum bioactivity of Compound 3.

The Bioactivity scores identified **Compound 3 (Pumiliotoxin** from ***C. caesia Roxb.)*** to have maximum biological activity (score > 0.0). This was followed by Bisacurone, EF31, UBS109, Compounds 88 and 91. We hypothesize that the physiological action exerted by these compounds could be due to interactions with GPCR and nuclear receptor ligands, modulating ion channel receptors and inhibiting protease, kinase and other enzymes.

Table 8 demonstrates the ADME properties of 6 compounds under study using Pre-ADMET and pkCSM tools.

**Table 8:** ADME properties of 6 compounds as predicted by preADMET and pkCSM.

Compound ID	Absorption			Distribution		Metabolism				Excretion	
	HIA	Caco2	MDCK	PPB	BBB	CYP2C19	CYP2C9	CYP3A4	CYP2D6	log(CL_tot_) (log ml/min/kg)	Renal OCT2
91 (CCR)	90.44	19.40	56.30	61.89	1.48	N	I	N	N	1.064	No
3	**91.45**	**38.05**	**56.08**	**85.74**	**3.21**	**N**	**N**	**N**	**N**	**0.938**	**No**
88	94.69	20.78	45.34	87.97	0.61	N	I	I	N	1.074	No
EF31 (CL)	95.99	44.79	32.46	54.10	0.08	N	N	N	N	0.816	No
UBS109	97.53	55.56	42.13	58.73	0.53	N	N	N	N	0.682	No
Bisacurone	90.98	24.61	321.05	88.34	0.45	I	I	N	N	1.382	No

*CCR Curcuma caesia Roxb*., *CL Curcuma longa L.*, *HIA* human intestinal absorption, *MDCK* madin-darby canine kidney, *PPB* plasma protein binding, *BBB* blood brain barrier, *CYP* cytochrome P450, *N* non inhibitor, *I* inhibitor, *CL*_*tot*_ total clearance, *OCT2* organic cation transporter 2.

Bold Compound 3 with favourable ADME properties.

#### Absorption (A)

The orally administered drugs are primarily absorbed in gut and intestine which can be precisely predicted using HIA value. The HIA of the six curcuma compounds were- 95.99 (EF31), 97.53 (UBS109), 90.98 (Bisacurone), 91.45 (Compound 3), 90.44 (Compound 91), and 94.69 (Compound 88); indicating higher absorption of these molecules. The permeability of a drug molecule is equal to its diffusion coefficient and human Caco-2 Adenocarcinoma cells and MDCK (Madin-Darby Canine Kidney) are widely used as in vitro models to predict drug permeability. In silico Caco-2 (4 < Caco-2 < 70) and MDCK (25 < MDCK < 500) permeability values for the six compounds ranged between 19.40–55.56 and 32.46–321.05 respectively predicting the compounds to be moderately permeable. This can result in moderate absorption across the gastrointestinal (GI) milieu followed by their distribution throughout the body.

#### Distribution (D)

The plasma protein binding (PPB) correlates to lipophilicity and dependent on concentration and number of binding sites of target protein. The degree of PPB affinity is directly proportional to the efficacy of bioactive compounds. The most important proteins involved in drug binding are- human serum albumin, alpha1-acid glycoprotein and lipoproteins. A compound being more lipophilic exerts stronger plasma-protein binding. The PPB assessment predicted that Compound 3, 88, and Bisacurone have moderate affinity (85.74–88.34%) while Compound 91, EF31, UBS109 (54.10–61.89%) showed weak affinity with plasma proteins. In general, a weak interaction with plasma protein indicates unrestricted transport across the cell membrane and free biomolecules to interact with the target and other proteins reducing bioavailability. The blood–brain barrier (BBB) protects the brain from exogenous compounds. A drug’s ability to cross the blood–brain barrier may, on one hand, increase toxicity when distributed through Central Nervous System (CNS) while on the other hand, may improve the drug functionality especially for targeting brain metastasis. The BBB penetration revealed, except EF31 (low < 0.1), Compound 3 having higher absorption (> 2.0), and Compound 88, 91, UBS109, and Bisacurone having moderate absorption (0.1–2.0) in CNS which qualifies them for eliminating cancer cells in the brain.

#### Metabolism (M)

The CYP450 system essentially aids in drug metabolism (M) and detoxification of substances from our system. Drugs that interact with CYP450 might get metabolized by one or multiple CYP450 enzymes and these drugs may either inhibit or induce cytochrome system. The inhibitors often results in unwanted drug interaction and delays the effect of candidate drugs. In our study, Compound 91 showed inhibition towards CYP2C9; Compound 88 inhibited CYP2C9, CYP3A4; and Bisacurone inhibited CYP2C19, CYP2C9. Selective inhibition of cytochrome P450 enzymes suggest that the compound may not exert higher toxicity or cause unwanted drug interactions. Compound 3, EF31, and UBS109 were non-inhibitors of cytochrome showing complete metabolism of these compounds. None of the compounds inhibited all the enzymes of the CYP450 system.

#### Excretion (E)

The elimination process of a drug molecule is termed as ‘clearance’ which is most predominantly performed by the kidneys post metabolism in the liver. The total clearance log(CL_tot_) with respect to hepatic and renal clearances as performed by pkCSM server, predicted that the rate of excretion was higher for Bisacurone (1.382); moderate for Compounds 88 (1.074) and 91 (1.064) and least for Compound 3(0.938), EF31(0.816), and UBS109 (0.682). Low clearance may be indicative of slow metabolism and thus increased half-life in the system. The Renal OCT2 (Organic Cation Transporter 2) is another excretion parameter determined by pkCSM. These protein transporters are important for renal uptake, disposition and clearance of a drug molecule. An OCT2 substrate can cause adverse interactions with inhibitors. None of the six compounds were found to be a substrate for Renal OCT2 thus predicting possibility of OCT2 dependent renal clearance of these compounds.

The Table 9 enlists the 6 compounds which are candidate for lead-likeness, and their toxicity. EF31 and UBS109 were predicted to be mutagen via Ames test but they were non-toxic to rat and mice models. Compound 88 was a non-mutagen; although it was found to be toxic for rat model but non-toxic for mice model. Compound 3, 91, and Bisacurone were non-mutagen and nontoxic. Additionally, the analysis of hepatotoxicity of the six compounds by pkCSM predicted only UBS109 was hepatotoxic. Besides this, the maximum recommended tolerated dose (MRTD) that estimates the threshold of dose producing an ‘acceptable level of toxicity’ was found to be higher for Bisacurone (0.705 (> 0.477 log mg/kg/day)) and lower (≤ 0.477 log mg/kg/day) for Compounds 3, 88, 91, EF31 and UBS109. Bisacurone may thus have the potential to cause an adverse effect which cannot be predicted unless tested in animal models. These six compounds were also found to satisfy lead-like criteria [250 ≤ MW ≤ 350; logP ≤ 3.5; rotatable bonds ≤ 7].

**Table 9:** Toxicity, drug-like and lead-like properties of six curcuma compounds as predicted by preADMET, pkCSM and SwissADME.

Compound ID	Druglikeness									Toxicity(mutagenecity/carcinogenicity)					Lead-likeness
	CMC	Lead	MDDR	Lipinski	WDI	Ghose	Veber	Egan	Muegge	Ames	Mice	Rat	Hepato-toxicity	MTD (log mg/kg/day)	
91 (CCR)	*	**	#	##	*#	Y	Y	Y	Y	N	-ve	-ve	N	0.329	Y
3	*	**	**#**	**##**	*#	**Y**	**Y**	**Y**	**Y**	**N**	**-ve**	**-ve**	**N**	− **0.172**	**Y**
88	*	**	#	##	*#	Y	Y	Y	Y	N	-ve	+ ve	N	0.444	Y
EF31 (CL)	*	**	#	##	*#	Y	Y	Y	Y	M	-ve	-ve	N	− 0.161	Y
UBS109	*	**	#	##	*#	Y	Y	Y	Y	M	-ve	-ve	Y	− 0.379	Y
Bisacurone	*	**	#	##	*#	Y	Y	Y	Y	N	-ve	-ve	N	0.705	Y

*CCR Curcuma caesia Roxb*., *CL Curcuma longa L.,* CMC—*—satisfied, Lead—**—Suitable for binding affinity is > 0.1 µM, MDDR—#- Mid-structure ranges only, Lipinski—##—Suitable, WDI—*#- 90% cut off, Ghose/Veber/Egan/Muegge- Y- Yes; Ames—M- Mutagen, N-Non mutagen/No; Carcinogenicity—+ ve- Positive, -ve—Negative; Hepatotoxicity- Y- yes, N- no; MTD- maximum tolerated dose; Y- yes; Bold- Compound 3 with favourable drug and lead-likeness properties and no toxicity.

*Considering the molecular properties, biological activities, ADME-Tox, drug, and lead-likeness criteria, we identified Compound 3 (Pumiliotoxin of C. Caesia Roxb.) could be a potent candidate for developing anticancer drug.*

### Prediction of metabolic sites of ligands

**Figure 2:**
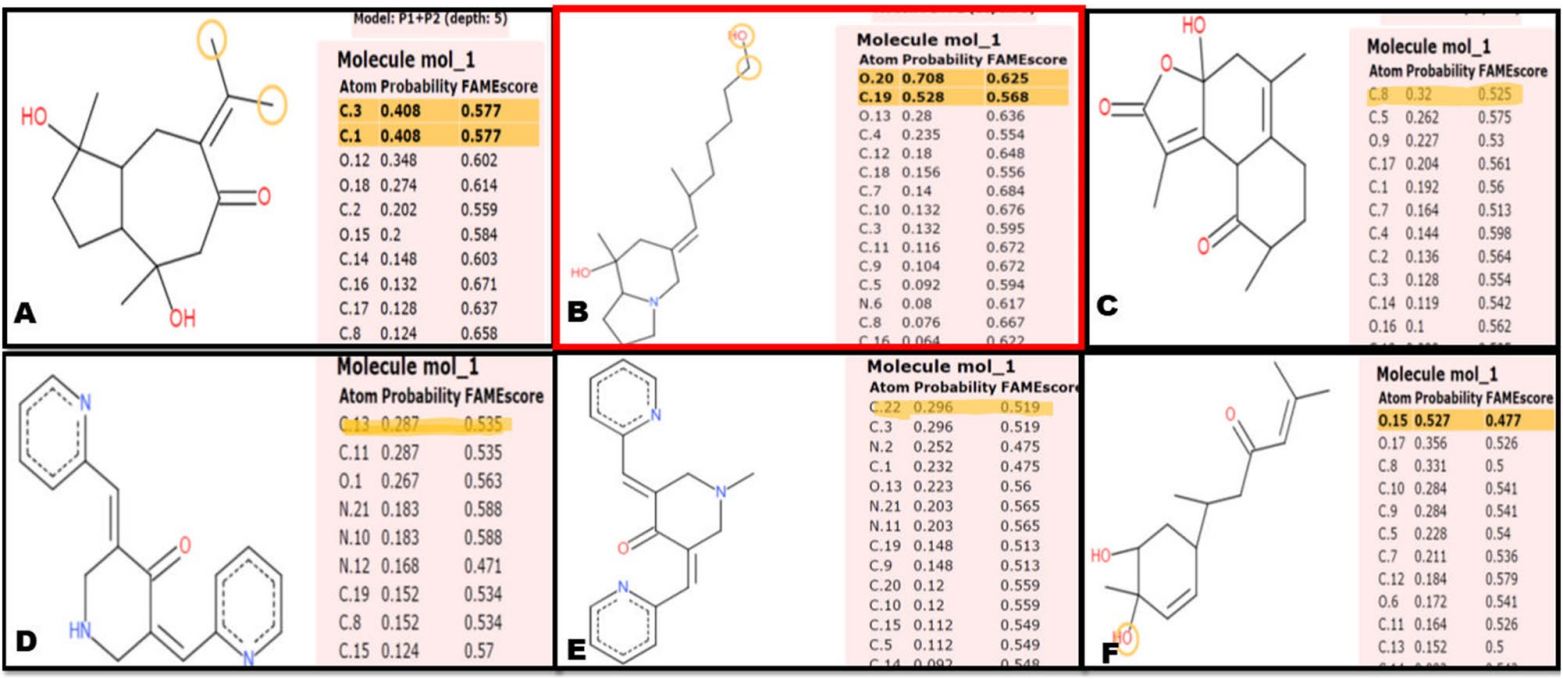
Site of Metabolism prediction of (**A**) Compound 91, (**B**) Compound 3, (**C**) Compound 88, (**D**) EF31, (**E**) UBS109, (**F**) Bisacurone.

The metabolic sites of the compounds are shown in Fig. 2. Among the 6 compounds, Compound 91 and 3 showed high metabolic sites at-carbon atom number 3 (score: 0.577), and 1 (score: 0.577); and oxygen atom number 20 (score: 0.625), carbon atom number 19 (score: 0.568) respectively. Compound 88, EF31, UBS109 showed moderate metabolic sites at carbon atom number 8 (score: 0.525); carbon atom number 13 (score: 0.535), and 11 (score: 0.535); carbon atom number 22 (score: 0.519), and 3 (0.519) respectively. Bisacurone was seen to have its metabolic site at oxygen atom number 15 (score: 0.477). All these sites interpret that the compounds could have the potential to initiate and carry out catalytic reactions that impact the various cellular functions, on administration.

### Protein targets for curcuminoids

The COSMIC database was used for selection and retrieval of proteins associated with haematologic malignancies. To perform molecular docking we selected 8 proteins viz. abl1 (v-abl Abelson murine leukaemia viral oncogene homolog 1), myc, max (myc-associated factor X), myb, pcna (Proliferating cell nuclear antigen), top3a (Topoisomerase 3α), p73, and blm (Bloom syndrome); molecular aberrations of which are known to be associated with malignant transformation (as per the mutational profiling) (Supplementary Links and Table S1–S8).

**Figure 3:**
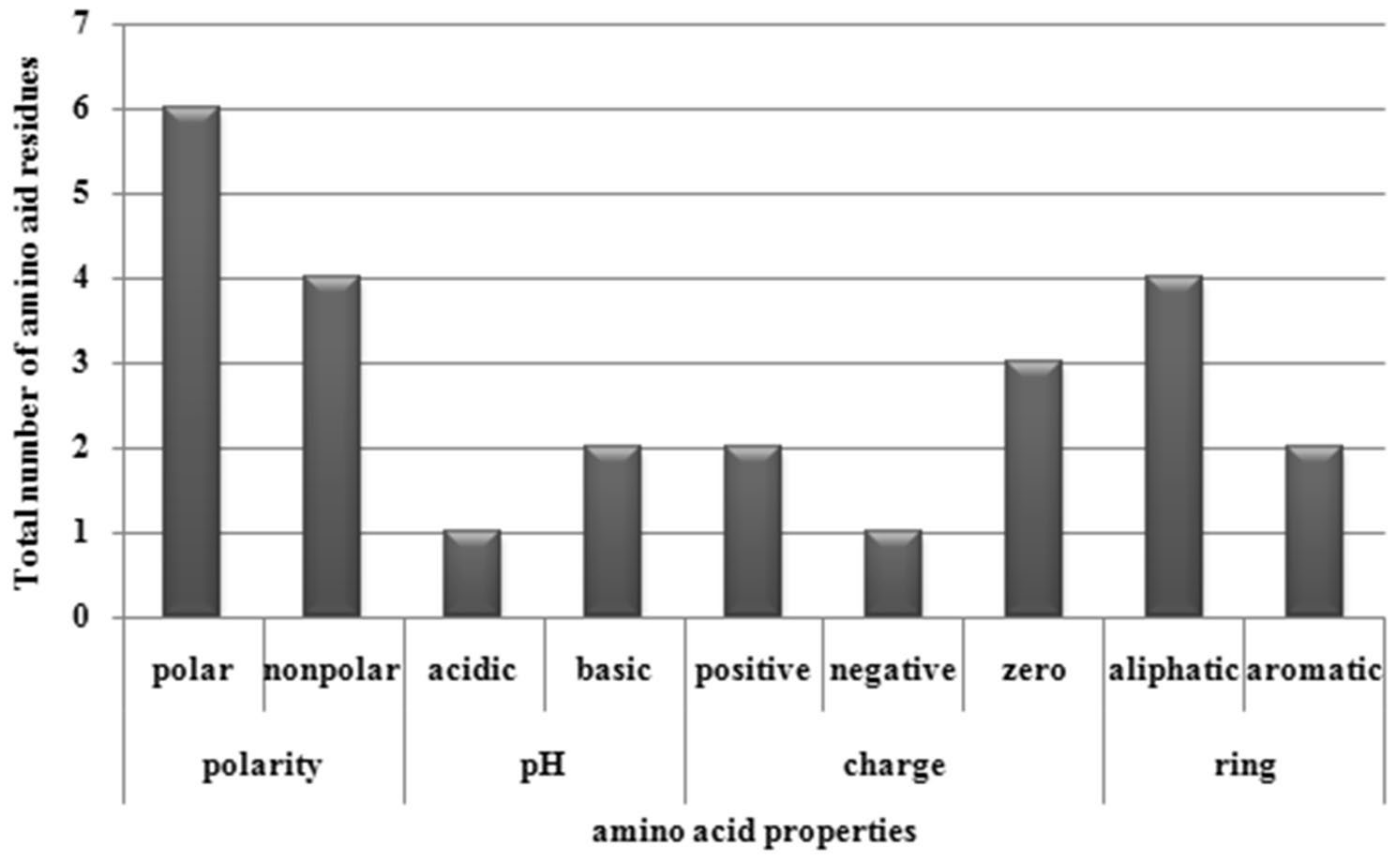
Contribution of different classes of amino acid residues at binding sites of ligand; X axis: amino acid properties; Y axis: Total number of amino acid residues.

The PDB IDs of the crystal structures of these proteins were- 2FO0 (**abl1**); 6G6K (**myc** and **max**); 1U7B (**pcna**); 4CGY (**top3a**); 1DXS, 2WQI, and 2XWC (**p73**); 4O3M and 5LUP (**blm**). The 3D structure of myb protein was modelled using Phyre2.0 and was validated using PROCHECK Ramachandran Plot analysis server due to unavailability of the structure in database. Approximately 55.2% residues were found to be in favoured region for myb (Supplementary Fig. 1A,B), Supplementary Table S9). The active sites of all the proteins were predicted using MetaPocket 2.0, the results of which are tabulated in Supplementary Table S10–S17. The output revealed that polar residues occurred at high frequency in active site architecture and participated in ligand binding by formation of hydrogen bonds. Figure 3 shows the contribution of different classes of amino acid residues at binding sites of ligand molecules.

For **abl1** protein, the binding site was enriched with valine (non-polar, aliphatic) and tyrosine (aromatic), and the active residues were distributed in binding pocket 1. In **myc** protein, the active site residues were observed in binding pocket 1, and the site was enriched with phenylalanine (aromatic), and lysine (positively charged, basic, polar, hydrophilic). In case of **max**, the most abundant active site residues were lysine, and arginine (positively charged, basic, polar, hydrophilic), glutamate (negatively charged, acidic, polar, hydrophilic), and glycine (non-polar, aliphatic), all of which were present in binding pocket 5. The residues leucine, and proline (non-polar, aliphatic), serine, and threonine (polar, non-charged) were ample in active site of **myb** protein and resided in binding pockets 1, 2 and 5. For **pcna**, glutamate (negatively charged, acidic amino acids, polar, hydrophilic) was abundant in binding pocket 1.The residues asparagine, and threonine (polar, non-charged), leucine, and proline (non-polar, aliphatic) were rich in binding pockets 1 and 2 of **top3a**. In case of, **p73**_**1DXS**_, the prominent residues were glutamate (negatively charged, acidic, polar, hydrophilic), leucine (non-polar, aliphatic), and serine (polar, non-charged); located in pockets 1, 2, 3, and 5. For **p73**_**2XWC**_, the binding pocket 1 was abundant with proline (non-polar, aliphatic). The residues leucine (non-polar, aliphatic) and arginine (positively charged, basic, polar, and hydrophilic) were present in binding pocket 1 of **blm**_**5LUP**_.

The protein domain analysis by scanPROSITE and MOTIF search revealed the active site residues to reside on- SH3 (residue no. 66–118) and protein kinase (residue no.242–492) domain of **abl1**; leucine zipper (residue no. 408–438) and helix-loop-helix DNA binding (residue no. 355–407) domains of **myc**; DUF5716 (Domain of Unknown Function) (residue no. 60–156) of **max**; LMSTEN (residue no. 267–313), C-terminal (residue no. 401–560), and DNA binding domain of **myb** (residue no. 41–86); N-terminal domain of **pcna** (residue no. 1–124); Topoisomerase (residue no. 196–603) and Toprim (residue no. 36–181) domains of **top3a**; SAM (residue no. 486–549), and DNA binding (residue no. 118–308) domains of **p73;** DEAD helicase (residue no. 671–838), RQC (residue no. 1072–1195) and BDHCT (residue no. 372–411) domains of **blm**. The detailed domain analysis results are provided in Supplementary Fig. 2 and Supplementary Table S18.

### Virtual screening of ligands with proteins involved in Haematologic Cancers

The preliminary analysis of 6 curcuma compounds (Compounds 91, 3, 88 and EF31, UBS109, Bisacurone) with desired molecular, biological and druglikeness properties against the target proteins (abl1, myc, max, myb, pcna, top3a, p73, and blm) was performed using AutoDock Vina to check for binding affinity. Table 10 enlists the binding affinity scores that ranged between -4.4 kcal/mol to -8.8 kcal/mol for the 6 ligands and 8 proteins under study. The maximum binding efficacy was exerted by black turmeric Compound 88 (Supplementary Fig. 3). This was followed by Compound 91, UBS109, EF31, Compound 3, and Bisacurone according to their descending order of binding affinity scores.

**Table 10:** Binding affinity of 6 compounds as predicted by AutoDock Vina.

Compound ID	abl1	myc	max	myb	pcna	top3a	p73			blm	
							1DXS	2WQI	2XWC	4O3M	5LUP
91 (CCR)	− 6.8	− 6.3	− 6.2	− 7.7	− 6.9	− 6.5	− 5.4	− 7.0	− 5.9	− 6.8	− 5.1
3	− 6.2	− 6.2	− 5.4	− 7.0	− 5.4	− 5.8	− 5.4	− 6.4	− 5.8	− 6.9	− 5.6
88	− **8.1**	**-6.6**	**-6.7**	**-8.8**	− **7.6**	− **6.9**	− **5.6**	− **6.8**	− **6.3**	− **7.3**	− **5.7**
EF31 (CL)	− 6.0	− 6.1	− 5.8	− 7.4	− 5.8	− 6.7	− 5.4	− 6.6	− 6.3	− 7.3	− 6.2
UBS109	− 6.2	− 5.7	− 5.6	− 8.3	− 6.8	− 6.9	− 5.3	− 7.1	− 6.2	− 7.5	− 6.0
Bisacurone	− 5.3	− 5.7	− 4.4	− 7.0	− 5.8	− 5.5	− 4.6	− 5.8	− 5.3	− 5.7	− 5.0

*CCR Curcuma caesia Roxb*., *CLCurcuma longa L.*

Bold Compound 88 showing maximum affinity towards target proteins.

### Analysis of ligand similarity

The Compound 3, Pumiliotoxin of *C. Caesia Roxb*., was aligned against ATRA (All Trans Retinoic Acid) using LS-align tool due to their conformational similarity as identified in our previous investigation (unpublished). The Compound 3 was submitted as query ligand and ATRA was submitted as template ligand. The PC score based rigid and flexible LS-align algorithm identified 12 out of 14 aligned pairs with identical atom types at distance < 1 Å. Thus, Compound 3 and ATRA were found to share approximately 85% atomic identity (Supplementary Fig. 4–5). This was also followed by molecular docking of ATRA with proteins of interest (myc and p73) as was Compound 3. The sites of interactions of ATRA to the two target proteins were in concordance with Pumiliotoxin to myc and p73 respectively (Supplementary Fig. 6).

### *Molecular docking of compounds and proteins *via* autodock tools*

The results obtained from AutoDock Vina were finally confirmed via molecular docking of 6 curcuma compounds against 8 proteins using AutoDock Tools. The results showed that the curcuma compounds were agonistic to the target proteins. Interestingly, while we considered the minimum hydrogen bond distance between the active pocket residues of proteins and ligands, the best docking results were observed for the following complexes—**Compound 91-abl1** (− 6.14 kcal/mol), **Compound 91-max** (− 5.29 kcal/mol); **Compound 3-myc** (− 4.48 kcal/mol), **Compound 3-p73**_**1DXS**_ (− 4.90 kcal/mol); **Compound 88-myb** (− 6.49 kcal/mol), **Compound 88-blm**_**5LUP**_ (− .87 kcal/mol); **UBS109-myb** (− 7.41 kcal/mol); **EF31-pcna** (− 6.76 kcal/mol); **Bisacurone-top3a** (− 4.52 kcal/mol). These complexes were further evaluated and discussed to understand their effect on modulating the MMR cascade. The calculated best binding energy, inhibition constants, and hydrogen (H) bond forming residues in protein active site along with the bond distances are summarized in Table 11.

**Table 11:** Best docking results for 6 ligands with selected proteins with respect to minimum hydrogen bond distance.

Compound ID	Proteins	Binding Energy (kcal/mol)	Residues involved in H-bonding	H-bond distance (Å)	Inhibition constant (µM)
91 (CCR)	abl1	− **6.14**	**Val92**	**3.49**	**31.55**
	max	− **5.29**	**Lys153**	**2.79**	**133.59**
3	myc	− **4.48**	**Glu935**	**4.91**	**518.26**
	p73_1DXS_	− **4.90**	**Asp41**	**2.95**	**255.75**
88	myb	− **6.49**	**Gln274**	**2.83**	**17.49**
	blm_5LUP_	− 4.87	Glu377, Arg407, Cys380	2.85, 3.66, 4.96	268.77
UBS109 (CL)	myb	− 7.41	Lys587	3.07	3.72
EF31	pcna	− 6.76	Glu124, Glu25	3.32, 3.20	11.00
Bisacurone	top3a	− 4.52	Tyr377, Asn406	3.11, 5.20	483.90

*CCR Curcuma caesia Roxb*., *CL Curcuma longa L.*

Bold Compounds 91, 3, and 88 showing best binding efficacy against abl1,max;myc, p73; and myb respectively.

**Figure 4:**
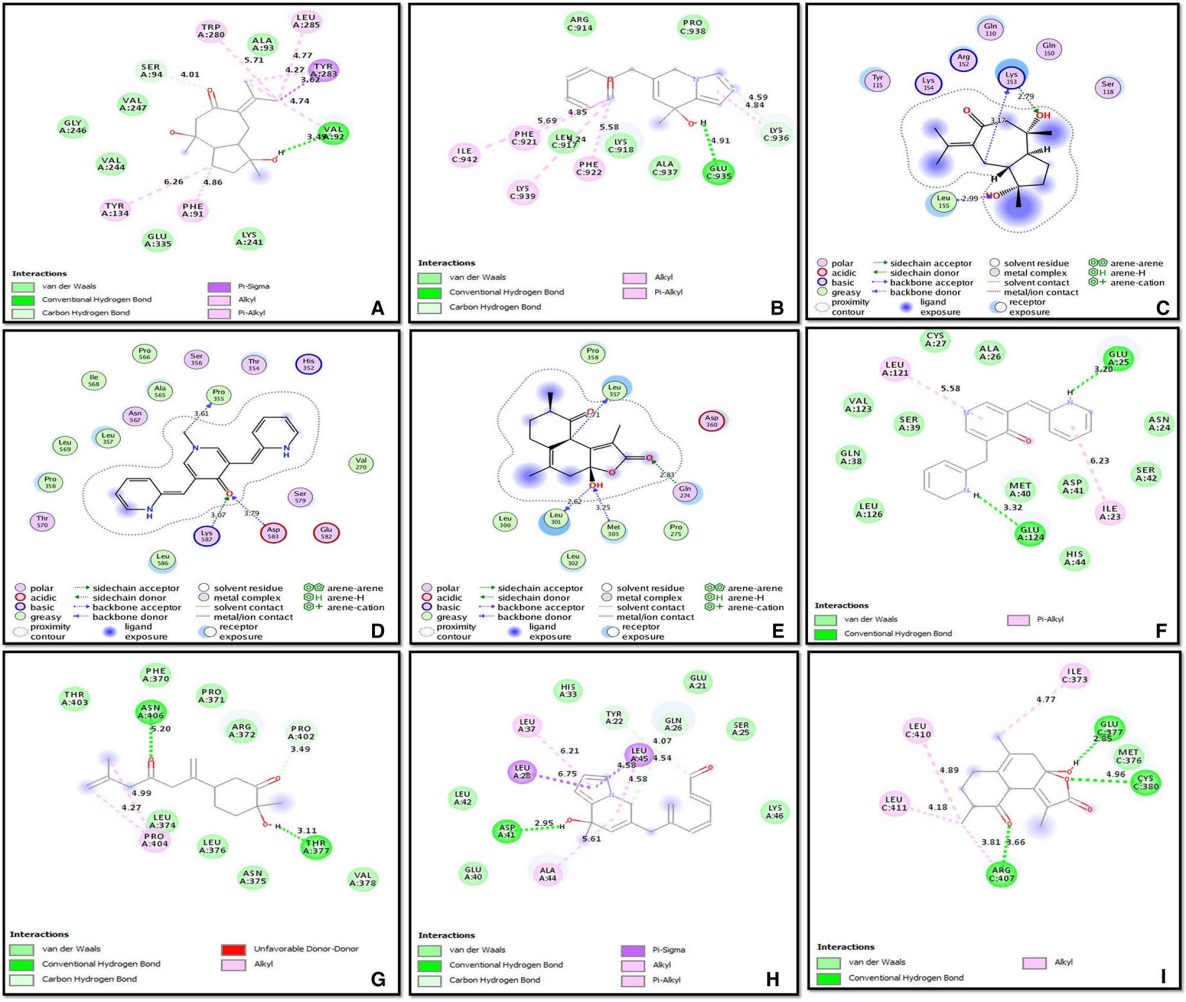
Best Docked complexes with respect to minimum hydrogen bond distance (**A**) Compound 91-abl1, (**B**) Compound 3-myc, (**C**) Compound 91-max, (**D**) UBS109-myb, (**E**) Compound 88-myb, (**F**) EF31-pcna, (**G**) Bisacurone-top3a, (**H**) Compound 3-p73_1DXS_, (**I**) Compound 88-blm_5LUP_.

The ligands were mainly found to interact with target proteins by means of hydrogen (H) bond. Additionally, some of the protein–ligand complexes- Bisacurone-abl1; UBS109-blm_5LUP_, top3a, and p73_2XWC_, Compound 3-myb, and Compound 88-p73_2WQI_ did not form any hydrogen bond with the active residues but interacted through other interatomic interactions viz., van der Waals interaction, C-H bonds, side chain donor, backbone donor and acceptor, pi-pi stack, pi-alkyl/alkyl, pi-sulphur, pi-sigma, pi-amide, and pi-cation/anion interactions. This was corroborated by Zhao and Huang (2011) who observed that H-bond alone might not be necessarily important for protein–ligand interactions^20^. Figure 4 represents the best docked complexes for each ligand to the target proteins with respect to minimum H-bond distance.

Overall, the binding energy scores of six ligands with target proteins ranged between − 3.33 and −7.41 kcal/mol (Table 12). According to their descending order of binding energy, UBS109 was found to be best fit for most of the target proteins (Supplementary Fig. 7). This was followed by EF31, Compounds 88, 91, 3 and Bisacurone respectively. Finally, the 8 proteins docked with curcuma compounds were studied for their interaction with mismatch repair system. STRING was used to carry out the interaction analysis.

**Table 12:** Binding energy scores of 6 ligands with target proteins as predicted from AutoDock Tools.

Compound ID	abl1	Myc	Max	myb	pcna	top3a	p73			Blm	
							1DXS	2WQI	2XWC	4O3M	5LUP
91 (CCR)	− 6.14	− 4.84	− 5.29	− 7.01	− 6.13	− 5.24	− 5.07	− 5.56	− 4.96	− 5.18	− 4.62
3	− 6.55	− 4.48	− 3.88	− 5.71	− 6.17	− 4.72	− 4.90	− 5.48	− 4.83	− 4.91	− 4.75
88	− 6.82	− 5.05	− 5.02	− 6.49	− 7.25	− 5.48	− 5.21	− 5.79	− 5.2	− 5.93	− 4.87
EF31 (CL)	− 6.20	− 5.33	− 5.37	− 6.98	− 6.76	− 5.67	− 5.63	− 6.12	− 6.65	− 5.41	− 6.33
UBS109	− **6.73**	− **5.39**	− **5.68**	− **7.41**	− **6.93**	− **6.01**	− **5.63**	− **6.13**	− **6.20**	− **5.54**	− **5.80**
Bisacurone	− 4.57	− 4.10	− 3.33	− 4.89	− 4.42	− 4.52	− 4.41	− 4.71	− 4.29	− 4.19	− 4.95

*CL Curcuma longa L.*, *CCR Curcuma caesia Roxb.*

Bold UBS109 showing maximum binding energy for target proteins.

**Figure 5:**
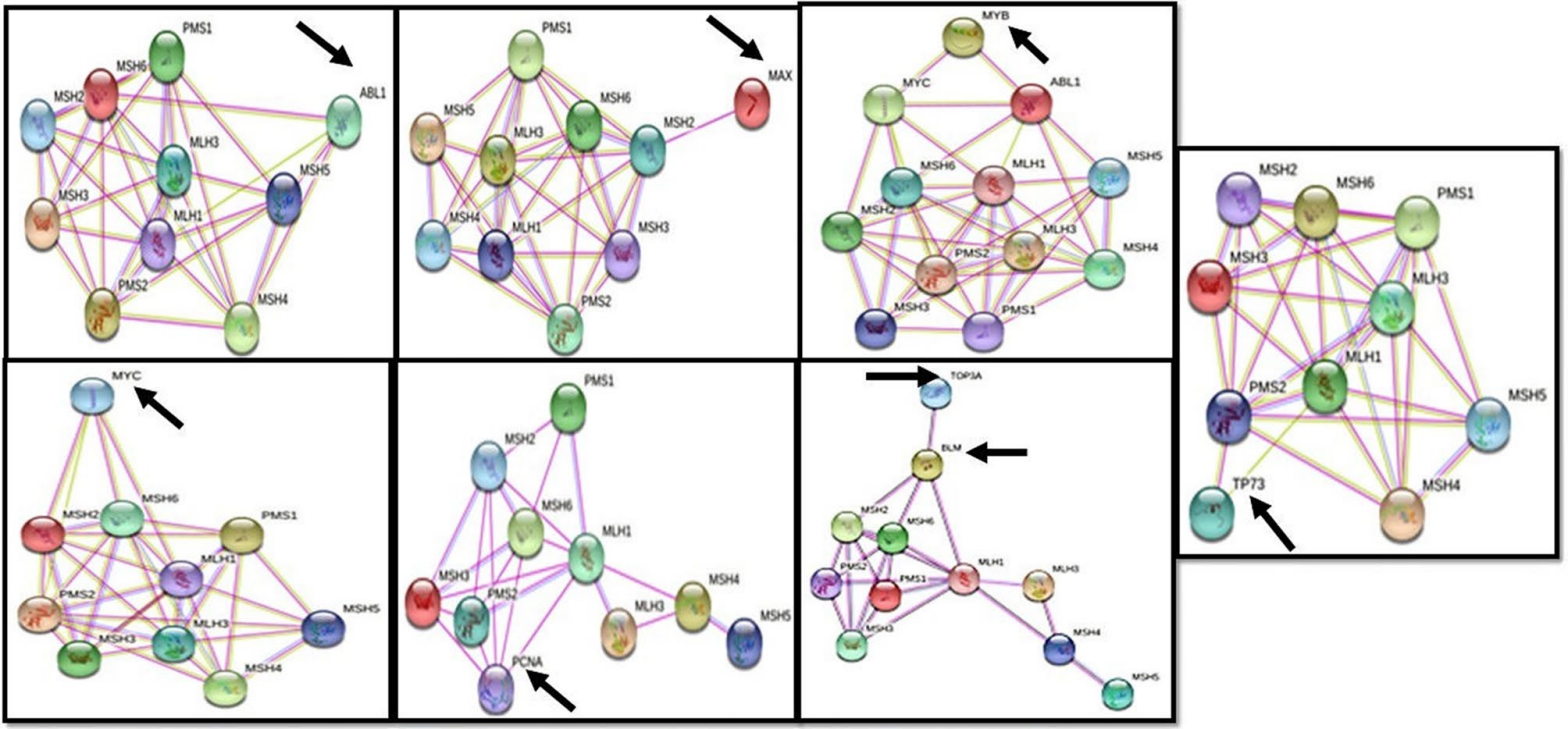
Interaction of the selected eight proteins involved in haematologic cancers (obtained from COSMIC) with any of the 8 MMR proteins as predicted by STRING; the arrows are indicative of the selected targets.

### Network analysis to understand proteins involved in haematologic cancers and their interactions with MMR

The STRING analysis identified the interaction of abl1, myc, max, myb, top3a, pcna, p73, and blm (score > 0.4) with mismatch repair proteins as interpreted by ‘experimental’ and ‘coexpression’ channels that analysed the data against KEGG. However, the ‘text mining’, ‘Databases’ and ‘Co-occurrence’ channels also helped in identifying additional inter-connection among the genes. Figure 5 shows the interactive network of the eight proteins with any of the 8 proteins of MMR system.

As per the ‘experimental’ and ‘coexpression’ channels, the following interactions were predicted—**abl1** with msh4, msh5, msh6; **myc** with msh2, msh6, mlh1; **max** with msh2; **pcna** with msh2, msh3, msh6, mlh1, pms2; **p73** with pms2; **blm** with msh2, msh6, mlh1. Additionally, **myb** showed its interaction with MMR via myc and abl1 and **top3a** showed its interaction with MMR via blm. The table below represents the scores of the most prominent interactions as predicted by STRING (Table 13).

**Table 13:** Interaction scores of target proteins with MMR.

Target proteins	Mismatch repair proteins	Interaction scores
abl1	msh5, msh6, msh4	0.751, 0.417, 0.464
myc	msh2, msh6, mlh1	0.618, 0.452, 0.626
max	msh2	0.520
myb-myc; myb-abl1	–	0.796;0.482
pcna	msh2, msh3, msh6, mlh1, pms2	0.817, 0.817, 0.964, 0.547, 0.547
top3a-blm	–	0.998
p73	pms2	0.535
blm	msh2, msh6, mlh1	0.589, 0.631, 0.994

## Discussion

**Figure 6:**
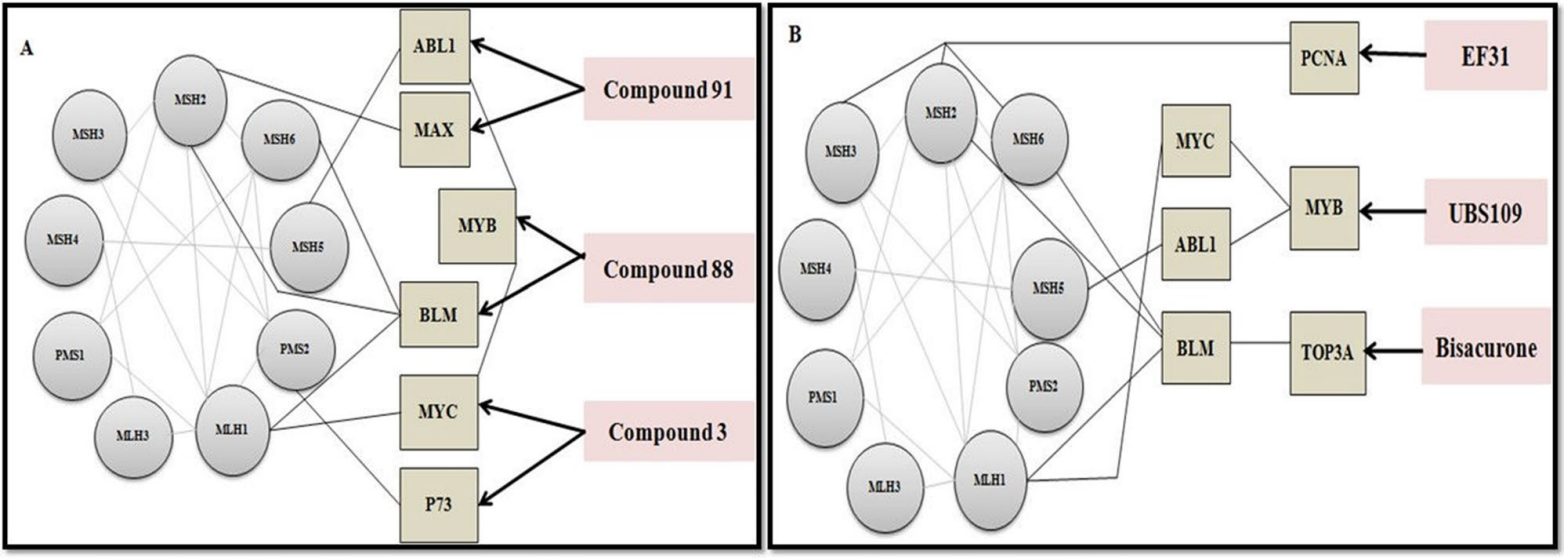
Schematic representation of possible implication of curcuma derivatives in activation of MMR system. (**A**) Shows compounds (91, 88, and 3) of *C. caesia Roxb*. interacting with their primary targets (abl1, max, myb, blm, myc, p73) which impact the MMR proteins (msh2, msh5, msh6, mlh1, pms2). (**B**) Depicts interactions that occur between the compounds (EF31, UBS109, and Bisacurone) of *C. longa L.* with their primary targets (pcna, myb, top3a) which further modulate MMR proteins (msh2, msh3, msh6, msh5, mlh1).

Cancer research has entered into an era of targeted therapeutics involving monoclonal antibodies, kinase inhibitors and immune checkpoint blockades. Despite targeting cancer associated biological pathways these treatments are limited by toxicities. In blood cancer, the current research is focused on CAR-T/NK treatments^21^. However, for over three decades now, plant products have secured a place in cancer treatment and these natural products have certainly come a long way as anticancer drugs. Curcumin was the first compound to be administered to human subjects in the year 1987 to observe its efficacy against cancer. Since then, it has been evaluated for wide range of biological activities in clinical perspective. Besides being cost-effective and capable of targeting multiple pathways, the curcuminoids limit treatment acquired resistance, show minimal side effects and might be used alone or in combination with existing therapies^22^. In the present study, we attempt to identify the efficacy of curcuma derived natural and synthetic compounds (*Curcuma longa L.* and *Curcuma caesia Roxb*.) against targeted proteins that cause deleterious consequences in haematologic malignancies via computational tools. Additionally, we infer that these proteins following the interaction with curcuma biomolecules may revive their own function and further rescue the expression of deregulated, non-mutated MMR protein in cancer. After initial screening of 536 curcuma compounds and further selecting 30 compounds based on their molecular, biological, and drug-like properties, we finalized 6 biomolecules viz., Compounds 3, 88, 91 from *C. caesia Roxb*., and EF31, UBS109, Bisacurone from *C. longa L*. The final docking with AutoDock tools helped us to recognize the H-bond dependent affinity of 6 curcuma compounds with eight targets. The overall docking analysis thus revealed the significance of the amino acid residues at active sites in creating a ‘local environment’ that aided recognition and binding of the ligands with target proteins. The figure below (Fig. 6) shows the possible implication of curcuma derivatives in rescuing MMR machinery during cancer treatment.

**Figure 7:**
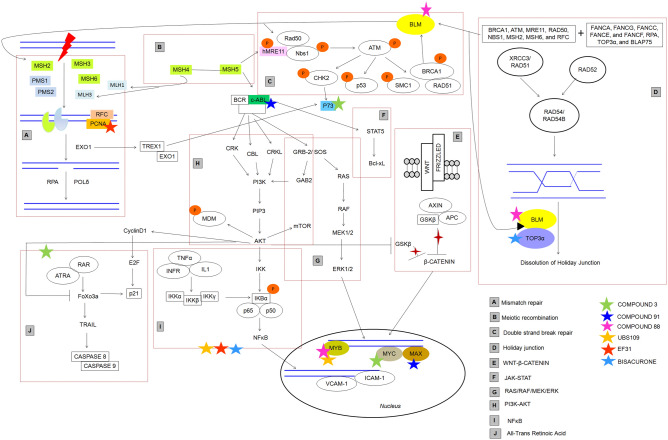
Diagrammatic representation of all the pathways involved as targets of anticancer biomolecules from Curcuma and their impact of Mismatch-Repair as elucidated from this study.

It is evident that the distinct types of leukaemia correlate with various forms of BCR-ABL oncogene which in turn activates MAPK, PI3K/Akt, NFκB, and STAT5 signaling pathways responsible for survival and proliferation of leukemic stem cells (LSCs)^23^. Piekarska et al*.* (2018) reported overexpression of abl1 in Philadelphia like ALL cases^24^. Despite availability of known tyrosine kinase inhibitors (TKIs) viz., imatinib, dasatinib, nilotinib, bosutinib, and ponatinib; the development of resistance due to acquisition of bcr-abl kinase domain mutations accompanied by toxic side effects, costs, and safety issues have subdued the fanaticism of using these drugs as ‘better choice’ in both CML (accelerated phase and blast crisis) and Ph^+^ ALL (Philadelphia positive Acute Lymphoblastic Leukaemia) cases^25–27^. The TKIs are also often not effective for genetically complex leukaemic cases due to the development of resistance towards TKIs. There are also possibilities of development of secondary malignancies following treatment with imatinib^28–30^. **Compound 91** was identified to be the best fit for abl1 (H-bond: Val^92^, distance: 3.49 Å) as understood through hydrogen-bond distance. The safety and efficacy of a modified Compound 91 in future can make it a good candidate to target abl1 differently than the current available drugs. Here, through STRING, abl1 shows direct interaction with MMR protein msh5^31^. Over-expression of msh5 has been reported in BCR-ABL positive CML^32^. Structural variations of MSH5 gene has been reported in T-cell ALL in a study conducted by Zhang and research group (2012)^33^. Disruption of functional msh5 protein leads to altered mismatch repair and affects double-strand break repair (DSBR) repair pathways (Fig. 7). The BCR-ABL positive CML (Chronic Myeloid Leukaemia) cells also lower the expression of mlh1 and pms2 and induce point mutations thus affecting the mismatch repair mechanisms^34,35^. Here we hypothesize that Compound 91 might pose a significant impact to eradicate the mutational events and overexpression of abl1 along with its fatal consequences on mismatch repair proteins involved in DSBR (Double Strand Break Repair) mechanisms.

In vitro and in vivo studies have shown that the binding of max with msh2, and myc with mlh1initiates the DNA repair process by formation of heterodimeric protein complexes^36^. A mutant max might lead to partial or complete loss of msh2 function, thus affecting MMR and causing loss of cellular apoptosis and genomic instability. It also affects the function of myc in normal and neoplastic conditions since myc forms sequence-specific DNA-binding complex with max^37^. **Compound 91** of ***C. caesia Roxb.*** was identified to be a decent agonist for max (H-bond: Lys^153^, distance: 2.79 Å). Therefore, this compound can possibly help to repress an overexpressed max by inhibiting myc-max dimerization and thus reducing DNA binding potential, and transcriptional activity of these proliferators in blood related cancers. Simultaneously, low expression of max will revive the function of msh2, and rewire the MMR. *To summarize this *in silico* data, Compound 91 can potentially target abl1, max, and myc pathways and revive DNA repair mechanisms.*

The constitutive dysregulation of myc protein is associated with its overexpression and poor prognosis in majority of human cancers including blood cancers. Downregulating myc has thus been a prime goal in anticancer therapies^38–40^ and it can be an ideal therapeutic target in haematologic malignancies as well. In the present study, we found **Compound 3** (Pumiliotoxin of ***C. caesia Roxb.***) having a good association with myc (H-bond: Glu^935^, minimum distance: 4.91 Å). Interestingly, myc also associates with mlh1 to regulate the mismatch repair^36^. Thus targeting myc with Pumiliotoxin (Compound 3) in blood cancer might help to upregulate mlh1 and direct the execution of damage recognition and repair. An interesting fact that came to light through our earlier studies (unpublished) is that, Compound 3 has a high resemblance to ATRA (All Trans Retinoic Acid) structurally as also inferred by LS-align here (Supplementary Fig. 4 and 5). ATRA has a role in downregulation of pin1 (Peptidylpropyl Cis/Trans Isomerase, NIMA-Interacting 1) in acute myeloid leukaemia (AML)^41^. Surprisingly, pin1 physically interacts with myc and both pin1 and myc are overexpressed in multiple cancers^42^. Downregulation of myc and pin1 via ATRA is already known^43,44^; however due to its short half-life it is not a very effective anticancer therapy. *Modification of Pumiliotoxin may therefore yield a novel and target driven future drug.*

Overexpression of p73 and its loss of expression as a result of hypermethylation were earlier reported in leukaemias and lymphomas^45,46^. Hence, protein p73 which is rarely mutated but frequently deregulated in cancer especially APL, requires therapeutic intervention^47^. Here, we identified **Compound 3** (− 4.9 kcal/mol; Asp^41^- 2.95 Å) to be best suited for binding with p73 and protecting the function of pms2. Under normal conditions, mismatch repair protein pms2 stabilizes p73 to stimulate p73-dependent apoptosis^48^. The requirement for pms2 in damage-induced activation of p73 is evident for direct signaling function of MMR proteins. Besides this, pms2 is a binding partner of mlh1. ATRA could be a potent modulator of aberrant p73 expression in haematologic cancers^47^. As mentioned above, structural resemblance has been found between Compound 3, and ATRA. *Thus, with proper modification of Compound 3, it may work as a good modulator of myc and p73 and aid the revival of MMR in various cancers.*

The overexpression, recurrent translocation and duplication of myb has been reported in AML, ALL, acute basophilic/myelomonocytic leukaemia, and adult T-cell leukaemia^49^. The MYB gene is indirectly connected to MMR via MYC and ABL1. A number of researches confirm that the binding of myb to the promoter regions of myc directly regulates the expression of myc protein^50^. The oncogenic myc and bcl-2 are known to be direct targets of myb^51^. This interdependency of myc and myb can be explored for therapeutic targeting. Similarly, bcr-abl1 transformed myeloid and lymphoid cells rely on aberrant expression of myb causing ‘addiction of leukemic cells towards myb’^52^. In our study we theorize, that indirect downregulation of abl1 and myc through Compound 91 and 3 respectively can alter the function of myb. Alternatively, we identified **Compound 88** (H-bond: Gln^274^, distance: 2.83 Å) from ***C. caesia Roxb***., and **UBS109 (**H-bond: Lys^587^, distance: 3.07 Å**)** from ***C. longa L.*** which prompted towards favourable binding with myb. These may modulate myb which in turn may downregulate the E2F1 transcriptional factor involved in creating a ‘second wave of transcription’ for progressing through aberrant cell cycle during cancer. The MMR genes MSH2 and MLH1 are known targets of E2F1^53^. While myc targets mlh1 and abl1 targets msh5 respectively, there is no direct interaction between myb and MMR proteins. These pathways may be explored in future for the various anticancer therapies.

Bloom syndrome patients develop haematologic malignancies frequently^54,55^. The yeast-two hybrid assay, co-immunoprecipitation and far western analysis confirmed the C-terminal region of blm to interact directly with mlh1 to maintain genomic stability^56^. Besides this, blm is also known to be regulated by msh2-msh6 heterodimeric complex^57^. We found black turmeric **Compound 88** docked best with protein blm (− 4.87 kcal/mol; H-bond: Glu^377^- 2.85 Å, Arg^407^- 3.66 Å, and Cys^380^- 4.96 Å). Hence, this natural compound of black turmeric might pose a significant impact on non-mutant deregulated blm expression such that its negative impact on MMR can be nullified.

PCNA is a central component of DNA replication and repair that interconnects MMR proteins msh3 and msh6. The pcna-msh3-msh6 complex, upon stacking on DNA, activates human MutS and MutL (MSH2-6 and MLH1,3; PMS1,2) components^58^. The elevated expression of pcna has been observed in multiple cancers including CML and CLL which correlates with poor survival^59^. A combined treatment of curcumin and doxorubicin was found to reduce expression of pcna in liver cancer^60^. Similar research investigated that curcumin alone or in combination with gemcitabine can suppress abnormally expressed PCNA in pancreatic cancer cells^61^. In present study, **EF31** formed best docked complex with pcna via H-bond formation with active residues Glu^124^ (3.32 Å) and Glu^25^ (3.20 Å). This suggests that the mutational effect of pcna can possibly be downregulated by EF31 to restore the MMR functionality but with minimum side effects that are exerted by doxorubicin or gemcitabine like drugs.

The top3a proteins though do not directly interact with MMR proteins, the BTR (BLM-TOP3A-RMI1/2) complex including blm and top3a are involved in DSBR^62,63^ wherein blm is known to interact with mlh1, msh2 and msh6. Overexpression of Topoisomerase has been recognized in multiple malignancies^64,65^. Here, **Bisacurone** showed its best binding affinity towards top3a by forming H-bond with active residues Tyr^377^ (H-bond distance: 3.11 Å) and Asn^406^ (H-bond distance: 5.2 Å). Thus we postulate, targeting mutant top3a with Bisacurone while also targeting blm with Compound 88 and UBS109, may cumulatively help in regulating the abnormal expression of top3a thereby impacting downstream effectors- proteins.

The figure below (Fig. 7) is a diagrammatic representation of the cancer-related pathways that can be targeted with curcuma compounds mentioned in this study, in order to protect and rewire the DNA mismatch repair system.

Several studies have investigated potential of curcumin, alone or in combination with other anticancer therapeutic drugs in modifying the expression of MMR proteins^66–68^. Shakibei et al. (2014) investigated effect of curcumin and 5-Fluorouracil on MMR deficient colorectal cancer (CRC) cells and found that curcumin not only increased the potency of 5-FU in a dose-dependent manner but also reduced the proliferation of MMR deficient tumor cells^66^. Chen et al. (2003) identified anti-leukemic mechanism of curcumin that elicited an increased expression of mismatch repair genes MLH1 and MSH2 followed by cellular apoptosis^67^. Jiang and colleagues (2010) investigated that MMR deficient CRC cells shows higher sensitivity towards curcumin which can be attributed to deregulation of multiple signaling cascades. Although curcumin induced oxidative damages were independent of MMR status; the activation of Chk1/2 and G2/M cell cycle arrest by curcumin requires intact MMR function^68^. From this computational study, we can suggest that Compounds 3 and 91 of *C. caesia Roxb.* had best drug like properties considering their interaction with myc, max, and abl1 respectively; the major contributors in emergence of haematologic malignancies. Additionally, Compound 88 and UBS109 bound well with protein myb. Recent investigations reported efficacy of UBS109, EF31 and Bisacurone against pancreatic cancer growth and breast cancer metastasis which majorly act upon NFκB and inhibit this cascade by suppressing IKKα and β^69–71^. However, the structure of UBS109 needs modification to reduce the mutagenecity and hepatotoxicity as predicted by the in silico tools in present study.

In a nutshell, the bioinformatics analyses revealed promising efficacy of the curcuma compounds against selective oncogenes and tumor suppressor gene; aberrations of which may possibly lead to deregulation of MMR system along with perturbation of functional inter and intra-molecular network. This study highlighted the significant protein–ligand interplay through various interatomic interactions and demonstrated the possible molecular mechanisms underlying the docking of these compounds with target proteins in haematologic malignancies. To our knowledge, till date there are no reports that computationally explored the anticancer potential of curcumin based ligands in haematologic malignancies with a focus on DNA mismatch repair machinery.

## Methodology

**Figure 8:**
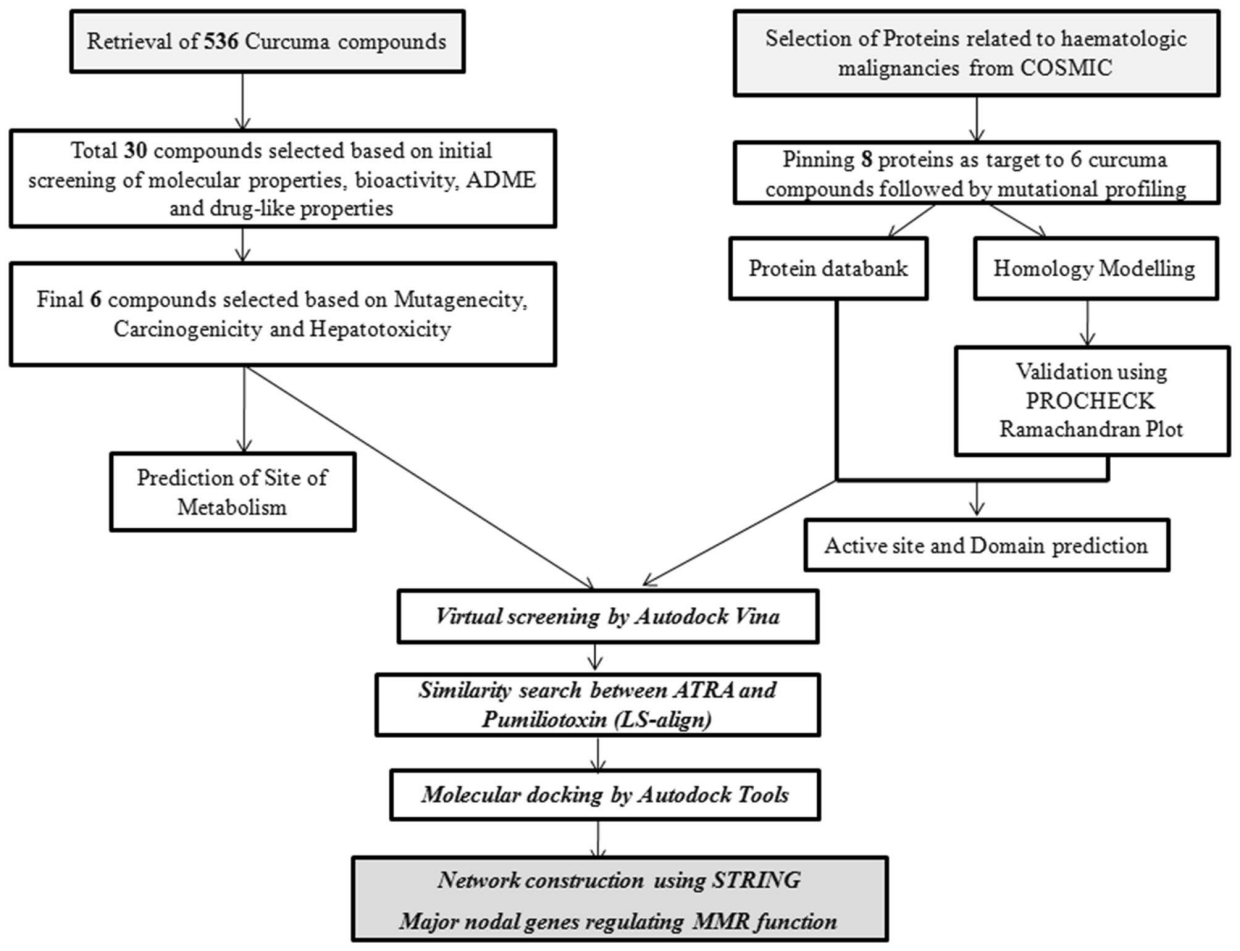
In silico Work-Flow of the present study.

The diagrammatic representation in Fig. 8 depicts the flow of work. The retrieval of chemical compounds and their docking with selected targets were carried out. This was followed by deriving the interaction map of target and MMR proteins effectively proving the impact of curcumin compounds on the function of MMR.

### Retrieval of ligands

A total of 536 curcuma compounds were retrieved from PubChem (through peer reviewed literature) (https://pubchem.ncbi.nlm.nih.gov/) and black turmeric database (http://blackspice.manipal.edu/) ^16,72,73^ (Supplementary Table S19). The structures were prepared using ChemDraw (https://www.perkinelmer.com/in/category/chemdraw) software and converted into .pdb format using Open Babel^74,75^.

### Calculation of molecular properties and bioactivity of ligands

The molecular properties and bioactivity of ligands were calculated using Molinspiration (https://www.molinspiration.com/). Fifteen descriptors analysed by Molinspiration were- molecular weight, logP, topological polar surface area (TPSA), volume, number of atoms, rotatable bonds, hydrogen bond donors and acceptors, range of violations to Lipinski's rule, and bioactivity. The bioactivity properties include- GPCR (G-protein coupled receptors) and nuclear receptor ligand, ion channel modulator, kinase, protease and enzyme inhibitors^76^.

### ADME-tox, drug and lead-likeness prediction of ligands

The ADME-Tox (PreADME/Tox prediction) (Absorption, Distribution, Metabolism, Excretion, Toxicity), water solubility, drug-like and lead-like properties of curcuma compounds were evaluated using PreADMET (https://preadmet.bmdrc.kr/), pkCSM (http://biosig.unimelb.edu.au/pkcsm/) and SwissADME (http://www.swissadme.ch/index.php) tools^77–79^. The ‘Absorption’ parameter checked for poor, moderate, and good absorption of the biomolecules, while the ‘Distribution’ parameter evaluated plasma protein binding (PPB- < 90%/ > 90%) and blood brain barrier (BBB- < 0.1/ 0.1–2.0/ > 2.0) values. The ‘Metabolism’ of the compounds was screened against Cytochrome P450 family of enzymes that include CYP2C9, CYP2C19, CYP2D6 and CYP3A4. The ‘Excretion’ of the compounds was calculated via the total clearance- log(CL_tot_) (log ml/min/kg) and Renal OCT2 (Organic Cation Transporter 2) values using pkCSM server. Mutagenecity and carcinogenicity were determined using in silico Ames test (mutagen/ non-mutagen) and toxicity (positive/negative) in rodent models. Besides this, pkCSM aided in prediction of hepatotoxicity and maximum tolerated dose (MTD) for a given compound. The SwissADME calculated physicochemical properties, lipophilicity, water solubility, pharmacokinetics, drug, and lead-likeness and medicinal chemistry related properties of a ligand molecule. In ‘Druglikeness’ parameter, the Lipinski’s rule of five, CMC (Comprehensive Medicinal Chemistry), lead-like, MDDR (Modern Drug Data Report), WDI (World.

Drug Index), Ghose, Veber, Egan, and Muegge rules were evaluated.

*All the biomolecules from various sources of Curcuma that satisfied the above criteria were further evaluated for energy minimization, virtual screening and docking.*

### Prediction of sites of metabolisms (SoMs) of compounds

FAME3 (https://nerdd.zbh.uni-hamburg.de/fame3/), the **FA**st**ME**tabolizer program, predicts the sites of metabolism (SoMs) in the atoms where a metabolic enzyme initiates a catalytic reaction^80^. In this study, prediction of such active sites gave us information of number of functionally interactive sites in the phytochemicals and in future can aid in designing drug derivatives.

### Retrieval and preparation of target proteins

The COSMIC (https://cancer.sanger.ac.uk/cosmic) database was used for selection of genes associated with haematologic malignancies^81^. A brief mutational profiling of the selected genes was carried out using PolyPhen-2 (http://genetics.bwh.harvard.edu/pph2/) and Meta-SNP (https://snps.biofold.org/meta-snp/)^82,83^. All the genes under study showed deleterious mutations in their coding regions suggesting that they contribute towards tumor initiation in the blood tissues they originate in. The search was restricted to blood-related cancers since it is the focus of this study. The RCSB Protein databank (https://www.rcsb.org/) was used to extract the crystal structures of the proteins of interest. Models were computed using Phyre2 for the proteins lacking a crystal structure in databank and validated using PROCHECK Ramachandran plot analyser (Protein Homology/analogY Recognition Engine2.0;http://www.sbg.bio.ic.ac.uk/~phyre2/html/page.cgi?id=index; https://servicesn.mbi.ucla.edu/PROCHECK/). Finally all the hetero-atoms, water molecules, and additional chains were removed from protein structures using Discovery Studio Visualizer prior to virtual screening and molecular docking.

### Protein active site prediction

Prior to docking, the prominent active binding sites of each protein molecule were predicted using MetaPocket2.0 (https://projects.biotec.tu-dresden.de/metapocket/index.php). The binding pockets, consisting of active residues for each protein, were identified which were later analysed compared to the docking results^84^.

### Domain analysis of proteins

MOTIF Search (https://www.genome.jp/tools/motif/) and ScanProsite (https://prosite.expasy.org/scanprosite/) were used to find the motifs/ domains of target proteins which may help in understanding the activity of the proteins.

### Energy minimization of ligands and proteins

The energy minimization of selected ligand molecules and proteins were performed using YASARA (Yet Another Scientific Artificial Reality Application) (http://www.yasara.org/minimizationserver.htm) which utilizes YASARA knowledge based potential force field^85^.

### Virtual screening of ligands

The virtual screening of ligand molecules were performed by AutoDock Vina software which utilizes a ‘gradient optimization method’ to improve its accuracy in prediction of binding affinity while minimizing the time^86^.

### Prediction of structural similarity between ligands

The LS-align (https://zhanglab.ccmb.med.umich.edu/LS-align/Database.html) tool was used to search for structural similarity between ligand molecules^87^.

### Molecular docking

Molecular docking was performed using AutoDock Tools 4.2.1 version^88^. The polar hydrogen was added to the receptor (proteins) followed by addition of Kollman charges and computing Gasteiger charges. The torsions were calculated for respective ligands and both receptor and ligand files were saved as .pdbqt format. The grid optimization was performed using AutoGrid programme and the grid box was centered such that it covers all identified active pocket amino acid residues. Docking was carried out using AutoDock programme and ten different conformations were generated with respect to their binding energies. The energy values in AutoDock are calculated on basis of various intermolecular bonds such as- hydrogen bond, desolvation energy, van der Waals, and electrostatic energy, internal energy of ligand, and torsional free energy. Amongst these, the desolvation and van der Waals energy together forms the binding energy; the hydrogen bond and van der Waals energy forms the docking energy and the strength of binding of ligand to the receptor is determined by electrostatic interactions. Complexes having lowest binding energy were considered as the best receptor-ligand structure and were chosen for post docking analysis. The results were visualized using Discovery Studio Visualizer and MOE (Molecular Operating Environment) softwares.

### Network construction

The STRING database (https://string-db.org/) was utilized to construct the interaction network between the target proteins selected in this study and proteins of the MMR system^89^. This aided in the understanding the impact of targets on the function of the MMR proteins and in future may find a way to modulate MMR, via the protective effect of curcuma compounds.

## Supplementary Information

Supplementary Information.
