# Supplementary material for: A study of deregulated MMR pathways and anticancer potential of curcuma derivatives using computational approach
Source: Sci Rep. 2021 May 12;11:10110. doi: 10.1038/s41598-021-89282-5 (PMC8115291; doi:10.1038/s41598-021-89282-5)
Supplement: Supplementary file 1 — Supplementary Information. [file 41598_2021_89282_MOESM1_ESM.docx]

**A Study of Deregulated MMR Pathways and Anticancer Potential of Curcuma Derivatives using Computational Approach**

**Priyanjali Bhattacharya^1^, Trupti N Patel^1*^**

**^1^Department of Integrative Biology, Vellore Institute of Technology, Vellore, India**

**^*^Corresponding Address- tnpatel@vit.ac.in, Dr.TNPatel@gmail.com**

**Supplementary Data**

**Distribution of Mutations among the Genes (selected) involved in Hematologic Malignancies from COSMIC Database and a brief mutational profile using various *in silico* tools**

Abbreviations -

*nsSNPs obtained from COSMIC; grey highlights- substitutions that are highly damaging considering ≥3 out of 5 different algorithms; N- Neutral, D- damaging, B-benign, NA- not applicable

- Link 1- ABL1;

<https://cancer.sanger.ac.uk/cosmic/gene/analysis?all_data=&coords=AA%3AAA&dr=&end=1150&gd=&id=368134&ln=ABL1&seqlen=1150&sn=haematopoietic_and_lymphoid_tissue&start=1#ts>

**Table S1:** *In silico* analysis of missense substitutions of abl1 protein

| **Mutation** | **Polyphen2.0** | **Panther** | **PhD-SNP** | **SIFT** | **SNAP** |
| --- | --- | --- | --- | --- | --- |
| G6R | 0.999 D | NA | 0.398 N | 0.180 N | 0.430 N |
| A53P | 0.009 B | NA | 0.475 N | 0.130 N | 0.380 N |
| A75T | 0.041 B | NA | 0.563 D | 0.060 N | 0.295 N |
| Y272H | 1.000 D | 0.548 D | 0.537 D | 0.000 D | 0.820 D |
| D344N | 0.900 D | 0.270 N | 0.446 N | 0.060 N | 0.210 N |
| N689S | 0.537 D | NA | 0.337 N | 0.890 N | 0.245 N |
| V806M | 0.483 D | NA | 0.389 N | 0.010 D | 0.565 D |
| T1084S | 0.649 D | NA | 0.680 D | 0.000 D | 0.560 D |
| K1099T | 0.997 D | NA | 0.801 D | 0.010 D | 0.720 D |
| C1119F | 0.999 D | NA | 0.878 D | 0.220 N | 0.655 D |
| E187K | 0.288 B | 0.251 N | 0.194 N | 0.090 N | 0.375 N |
| D295G | 0.243 B | 0.520 D | 0.204 N | 0.040 D | 0.460 N |
| E301V | 0.991 D | 0.530 D | 0.675 D | 0.000 D | 0.600 D |
| T334I | 0.999 D | 0.496 N | 0.736 D | 0.020 D | 0.470 N |
| T334L | 1.000 D | 0.463 N | 0.713 D | 0.030 D | 0.765 D |
| A621T | 0.280 B | NA | 0.421 N | 0.300 N | 0.200 N |
| P937L | 0.018 B | NA | 0.270 N | 0.000 D | 0.675 D |
| A1010T | 0.000 B | NA | 0.093 N | 0.000 D | 0.605 D |
| G60D | 0.016 B | NA | 0.575 D | 0.870 N | 0.285 N |
| G269E | 0.975 D | 0.606 D | 0.332 N | 0.150 N | 0.425 N |
| F330I | 0.915 D | 0.462 N | 0.116 N | 0.290 N | 0.165 N |
| F336R | 1.000 D | 0.637 D | 0.852 D | 0.000 D | 0.765 D |
| F336L | 0.942 D | 0.292 N | 0.373 N | 0.280 N | 0.445 N |
| M370T | 1.000 D | 0.953 D | 0.905 D | 0.000 D | 0.705 D |
| E374G | 1.000 D | 0.529 D | 0.781 D | 0.000 D | 0.630 D |
| F378I | 0.992 D | 0.501 D | 0.776 D | 0.040 D | 0.515 D |
| L389R | 1.000 D | 0.991 D | 0.923 D | 0.000 D | 0.715 D |
| H415R | 0.002 B | 0.544 D | 0.316 N | 0.130 N | 0.300 N |

- Link 2- MYC gene;

<https://cancer.sanger.ac.uk/cosmic/gene/analysis?all_data=&coords=AA%3AAA&dr=&end=455&gd=&id=359910&ln=MYC&seqlen=455&sn=haematopoietic_and_lymphoid_tissue&start=1#ts>

**Table S2:** *In silico* analysis of missense substitutions of myc protein

| **Mutation** | **Polyphen2.0** | **Panther** | **PhD-SNP** | **SIFT** | **SNAP** |
| --- | --- | --- | --- | --- | --- |
| Q11R | 0.167 B | NA | 0.621 D | NA | 0.435 N |
| S21N | 0.001 B | 0.154 N | 0.315 N | NA | 0.390 N |
| A59V | 1.000 D | 0.593 D | 0.193 N | NA | 0.450 N |
| P72L | 0.999 D | 0.982 D | 0.559 D | NA | 0.590 D |
| T73A | 0.663 D | 0.766 D | 0.341 N | NA | 0.415 N |
| T73I | 0.981 D | 0.919 D | 0.483 N | NA | 0.565 D |
| T73N | 0.999 D | 0.913 D | 0.531 D | NA | 0.425 N |
| P74A | 1.000 D | 0.916 D | 0.289 N | NA | 0.620 D |
| P74L | 1.000 D | 0.960 D | 0.446 N | NA | 0.590 D |
| P74Q | 1.000 D | 0.971 D | 0.546 D | NA | 0.625 D |
| P74R | 1.000 D | 0.961 D | 0.433 N | NA | 0.660 D |
| P74S | 1.000 D | 0.930 D | 0.383 N | NA | 0.625 D |
| P75S | 0.999 D | 0.450 N | 0.320 N | NA | 0.585 D |
| L97V | 0.058 D | 0.047 N | 0.372 N | NA | 0.280 N |
| R98Q | 0.856 D | 0.067 N | 0.347 N | NA | 0.440 N |
| N127K | 0.170 B | 0.168 N | 0.516 D | 0.020 D | 0.500 N |
| F130L | 0.562 D | 0.208 N | 0.507 D | 0.160 N | 0.445 N |
| F153S | 0.991 D | 0.935 D | 0.849 D | 0.030 D | 0.660 D |
| A156S | 0.481 D | 0.221 N | 0.279 N | 0.220 N | 0.130 N |
| S161L | 0.840 D | 0.367 N | 0.555 D | 0.030 D | 0.490 N |
| V6G | 0.000 B | NA | 0.448 N | NA | 0.550 D |
| Y47C | 0.999 D | 0.862 D | 0.481 N | NA | 0.665 D |
| Q52R | 0.372 B | 0.432 N | 0.074 N | NA | 0.475 N |
| S53T | 0.955 D | 0.298 N | 0.083 N | NA | 0.425 N |
| E54D | 0.056 B | 0.187 N | 0.074 N | NA | 0.490 N |
| A59V | 1.000 D | 0.593 D | 0.193 N | NA | 0.450 N |
| L71V | 0.999 D | 0.782 D | 0.110 N | NA | 0.520 D |
| P72S | 0.778 D | 0.969 D | 0.451 N | NA | 0.630 D |
| P72T | 0.995 D | 0.976 D | 0.450 N | NA | 0.635 D |
| Y89H | 0.965 D | 0.254 N | 0.260 N | NA | 0.415 N |
| D102G | 0.000 B | 0.199 N | 0.359 N | NA | 0.605 D |
| I144L | 0.486 D | 0.258 N | 0.617 D | 0.310 N | 0.375 N |
| F153C | 0.998 D | 0.971 D | 0.827 D | 0.010 D | 0.705 D |
| S189V | 0.045 D | 0.364 N | 0.182 N | 0.210 N | 0.345 N |
| L191M | 0.988 D | 0.369 N | 0.114 N | 0.080 N | 0.330 N |

- Link 3- MAX gene;

<https://cancer.sanger.ac.uk/cosmic/gene/analysis?all_data=&coords=AA%3AAA&dr=&end=161&gd=&id=209776&ln=MAX&seqlen=161&sn=haematopoietic_and_lymphoid_tissue&start=1#distribution>

**Table S3:** *In silico* analysis of missense substitutions of max protein

| **Mutation** | **Polyphen2.0** | **Panther** | **PhD-SNP** | **SIFT** | **SNAP** |
| --- | --- | --- | --- | --- | --- |
| R35H | 1.000 D | 0.816 D | 0.672 D | NA | NA |
| R36W | 1.000 D | 0.941 D | 0.715 D | NA | NA |
| D37G | 1.000 D | 0.708 D | 0.661 D | NA | NA |
| N120T | 0.000 B | 0.067 N | 0.170 N | NA | NA |
| R60Q | 1.000 D | 0.661 D | 0.404 N | NA | NA |

- Link 4- MYB gene;

<https://cancer.sanger.ac.uk/cosmic/gene/analysis?all_data=&coords=AA%3AAA&dr=&end=762&gd=&id=303929&ln=MYB&seqlen=762&sn=haematopoietic_and_lymphoid_tissue&start=1#distribution>

**Table S4:** *In silico* analysis of missense substitutions of myb protein

| **Mutation** | **Polyphen2.0** | **Panther** | **PhD-SNP** | **SIFT** | **SNAP** |
| --- | --- | --- | --- | --- | --- |
| E14K | 0.900 D | NA | 0.590 D | 0.020 D | 0.660 D |
| E14Q | 0.967 D | NA | 0.308 N | 0.020 D | 0.585 D |
| K35N | 0.996 D | NA | 0.550 D | 0.010 D | 0.650 D |
| N68I | 0.055 B | NA | 0.268 N | 0.070 N | 0.565 D |
| N68Y | 0.864 D | NA | 0.283 N | 0.040 D | 0.665 D |
| W82R | 1.000 D | 0.939 D | 0.864 D | 0.260 N | 0.865 D |
| S146P | 0.170 B | 0.703 D | 0.224 N | 0.160 N | 0.470 N |
| E299K | 1.000 D | 0.305 N | 0.806 D | 0.020 D | 0.575 D |
| L301F | 1.000 D | 0.269 N | 0.564 D | 0.040 D | 0.500 N |
| T427I | 0.096 B | NA | 0.210 N | 0.250 N | 0.325 N |
| C322Y | 0.210 B | 0.267 N | 0.566 D | 0.030 D | 0.690 D |
| S745T | 0.015 B | NA | 0.366 N | 0.250 N | 0.270 N |
| F661Y | 0.997 D | NA | 0.470 N | 0.000 D | 0.555 D |
| R510S | 0.017 B | NA | 0.057 N | 0.430 N | 0.450 N |
| D408G | 0.995 D | NA | 0.651 D | 0.390 N | 0.470 N |
| W82R | 0.997 D | 0.939 D | 0.864 D | 0.000 D | 0.900 D |
| T427I | 0.096 B | NA | 0.204 N | 0.020 D | 0.520 D |
| K387N | 0.791 D | 0.212 N | 0.594 D | 0.390 N | 0.345 N |

- Link 5- PCNA gene;

<https://cancer.sanger.ac.uk/cosmic/gene/analysis?all_data=&coords=AA%3AAA&dr=&end=262&gd=&id=343943&ln=PCNA&seqlen=262&sn=haematopoietic_and_lymphoid_tissue&start=1#ts>

**Table S5:** *In silico* analysis of missense substitutions of pcna protein

| **Mutation** | **Polyphen2.0** | **Panther** | **PhD-SNP** | **SIFT** | **SNAP** |
| --- | --- | --- | --- | --- | --- |
| R61H | 0.971 D | 0.504 D | 0.712 D | 0.030 D | 0.680 D |

- Link 6- TOP3A gene;

<https://cancer.sanger.ac.uk/cosmic/gene/analysis?all_data=&coords=AA%3AAA&dr=&end=1002&gd=&id=280375&ln=TOP3A&seqlen=1002&sn=haematopoietic_and_lymphoid_tissue&start=1#distribution>

**Table S6:** *In silico* analysis of missense substitutions of top3a protein

| **Mutation** | **Polyphen2.0** | **Panther** | **PhD-SNP** | **SIFT** | **SNAP** |
| --- | --- | --- | --- | --- | --- |
| E398K | 0.302 B | 0.527 D | 0.385 N | 0.390 N | 0.290 N |
| A461T | 0.995 D | 0.745 D | 0.339 N | 0.050 D | 0.515 D |
| T504N | 0.026 D | 0.600 D | 0.109 N | 0.500 N | 0.095 N |
| P514T | 1.000 D | 0.855 D | 0.702 D | 0.000 D | 0.645 D |
| I647T | 0.008 B | 0.317 N | 0.242 N | 0.620 N | 0.290 N |
| R722C | 0.990 D | 0.904 D | 0.733 D | 0.150 N | 0.620 D |
| V811M | 0.967 D | 0.614 D | 0.532 D | 0.060 N | 0.530 D |
| P990S | 0.959 D | NA | 0.271 N | 0.040 D | 0.615 D |

- Link 7- P73 gene;

<https://cancer.sanger.ac.uk/cosmic/gene/analysis?all_data=&coords=AA%3AAA&dr=&end=637&gd=&id=386532&ln=TP73&seqlen=637&sn=haematopoietic_and_lymphoid_tissue&start=1#distribution>

**Table S7:** *In silico* analysis of missense substitutions of p73 protein

| **Mutation** | **Polyphen2.0** | **Panther** | **PhD-SNP** | **SIFT** | **SNAP** |
| --- | --- | --- | --- | --- | --- |
| H17L | 0.998 D | NA | 0.254 N | 0.030 D | 0.500 N |
| P111L | 1.000 D | NA | 0.398 N | 0.110 N | 0.445 N |
| S145C | 1.000 D | 0.959 D | 0.663 D | 0.000 D | 0.745 D |
| V191M | 1.000 D | 0.897 D | 0.727 D | 0.000 D | 0.640 D |
| P195L | 0.780 D | 0.963 D | 0.771 D | 0.040 D | 0.605 D |
| E205K | 0.607 D | 0.421 N | 0.440 N | 0.050 N | 0.515 D |
| G219D | 0.993 D | 0.954 D | 0.845 D | 0.080 N | 0.545 D |
| Y487D | 1.000 D | 0.684 D | 0.687 D | 0.060 N | 0.655 D |
| I626V | 0.995 D | NA | 0.376 N | 0.230 N | 0.530 D |

- Link 8- BLM gene;

<https://cancer.sanger.ac.uk/cosmic/gene/analysis?all_data=&coords=AA%3AAA&dr=&end=1418&gd=&id=399159&ln=BLM&seqlen=1418&sn=haematopoietic_and_lymphoid_tissue&start=1#ts>

**Table S8:** *In silico* analysis of missense substitutions of blm protein

| **Mutation** | **Polyphen2.0** | **Panther** | **PhD-SNP** | **SIFT** | **SNAP** |
| --- | --- | --- | --- | --- | --- |
| F32I | 0.046 B | NA | 0.333 N | 0.060 N | 0.470 N |
| R85T | 0.002 B | NA | 0.248 N | 0.000 D | 0.640 D |
| M348I | 0.146 B | NA | 0.341 N | 0.060 N | 0.535 D |
| G397W | 1.000 D | NA | 0.867 D | 0.000 D | 0.705 D |
| S601Y | 0.947 D | NA | 0.354 N | 0.020 D | 0.550 D |
| R643H | 0.004 B | 0.325 N | 0.378 N | 0.520 N | 0.240 N |
| S646G | 0.000 B | 0.081 N | 0.095 N | 1.000 N | 0.125 N |
| Q672R | 1.000 D | 0.995 D | 0.885 D | 0.000 D | 0.870 D |
| R791C | 0.981 D | 0.885 D | 0.795 D | 0.010 D | 0.595 D |
| H805Y | 1.000 D | 0.864 D | 0.853 D | 0.000 D | 0.745 D |
| L879V | 0.063 B | 0.082 N | 0.306 N | 0.280 N | 0.185 N |
| H886N | 0.048 B | 0.357 N | 0.446 N | 0.040 D | 0.455 N |
| D906N | 0.018 B | 0.183 N | 0.192 N | 0.120 N | 0.080 N |
| Q909R | 0.013 B | 0.145 N | 0.212 N | 0.260 N | 0.260 N |
| P956L | 0.992 D | 0.779 D | 0.862 D | 0.000 D | 0.490 N |
| Y1044C | 1.000 D | 0.841 D | 0.924 D | 0.000 D | 0.745 D |
| D1076H | 1.000 D | 0.552 D | 0.691 D | 0.000 D | 0.755 D |
| V1077M | 0.890 D | 0.567 D | 0.205 N | 0.090 N | 0.240 N |
| D1080N | 0.699 D | 0.276 N | 0.267 N | 0.090 N | 0.180 N |
| V1198M | 0.017 B | 0.232 N | 0.195 N | 0.020 D | 0.270 N |
| V1321I | 0.010 B | NA | 0.084 N | 0.230 N | 0.370 N |
| V1321L | 0.092 B | NA | 0.443 N | 0.200 N | 0.495 N |
| S1368Y | 0.906 D | NA | 0.569 D | 0.030 D | 0.580 D |

**Model and Structure Validation of myb protein using Phyre2 and PROCHECK Ramachandran Plot**

**
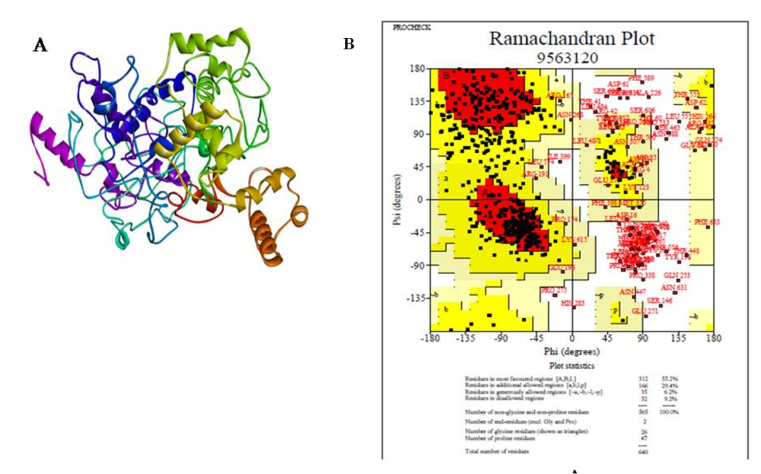
**

**Figure 1:** (A) 3D model of myb protein (B) Ramachandran plot of myb protein

**Table S9:** PROCHECK Ramachandran plot analysis result of myb protein

| **Protein** | **No. of residues in most favoured regions** | **No. of residues in additional allowed regions** | **No. of residues in generously allowed regions** | **No. of residues in disallowed regions** |
| --- | --- | --- | --- | --- |
| **myb** | 55.2% | 29.4% | 6.2% | 9.2% |

**Active Binding Site and Residues involved in the target proteins using MetaPocket2.0**

**Table S10:** Active binding site residues of abl1

| BINDING POCKET 1 | ALA_A^93^ SER_A^94^ TYR_A^283^ LEU_A^285^ VAL_A^92^  LYS_A^282^ SER_A^284^ VAL_A^247^ PHE_A^91^ TRP_A^280^  LEU_A^321^ GLY_A^246^ VAL_A^244^ SER_A^248^ VAL_A^287^  GLU_A^335^ TYR_A^245^ THR_A^286^ PHE_A^336^ GLN_A^319^  LYS_A^241^ TYR_A^134^ THR_A^243^ PRO_A^242^ ASP_A^90^  MET_A^337^ THR_A^338^ LYS_A^397^ LEU_A^320^ ASN_A^240^  GLU_A^392^ LEU_A^395^ ASN_A^133^ PRO_A^315^ GLY_A^391^  HIS_A^314^ LEU_A^88^ TYR_A^89^ SER_A^132^ ILE_A^135^  TYR_A^339^ THR_A^136^ ASN_A^393^ GLU_A^142^ ASN_A^316^  ARG_A^239^ LYS_A^313^ SER_A^368^ TYR_A^372^ LYS_A^375^  GLU_A^371^ LYS_A^238^ PRO_A^150^ TYR_A^147^ GLN_A^365^  GLU_A^174^ HIS_A^148^ GLY_A^149^ HIS_A^394^ TYR_A^361^  VAL_A^396^ HIS_A^144^ VAL_A^151^ HIS_A^509^ TYR_A^158^  LEU_A^159^ THR_A^364^ PRO_A^237^ ALA_A^155^ GLU_A^513^  SER_A^152^ SER_A^175^ GLU_A^172^ GLN_A^510^ SER_A^173^  ASN_A^154^ ARG_A^153^ VAL_A^357^ LEU_A^360^ ARG_A^171^  GLN_A^179^ THR_A^514^ SER_A^176^ SER_A^181^ ARG_A^180^  GLN_A^517^ ARG_A^194^ HIS_A^192^ PHE_A^516^ TYR_A^193^  GLU_A^518^ LYS_A^376^ |
| --- | --- |
| BINDING POCKET 2 | HIS_A^314^ PRO_A^315^ ASN_A^316^ SER_A^368^ PRO_A^150^  GLU_A^174^ ARG_A^239^ VAL_A^151^ SER_A^152^ ALA_A^155^  GLU_A^513^ SER_A^175^ GLN_A^510^ ARG_A^153^ SER_A^173^  ARG_A^171^ GLU_A^172^ GLN_A^517^ THR_A^514^ TYR_A^158^  LEU_A^159^ ASN_A^393^ LYS_A^238^ PRO_A^237^ SER_A^162^  TYR_A^361^ TYR_A^89^ HIS_A^148^ ASN_A^240^ TYR_A^147^  GLY_A^149^ SER_A^161^ HIS_A^394^ GLN_A^352^ GLU_A^353^  ASN_A^355^ VAL_A^358^ ASN_A^154^ GLU_A^157^ HIS_A^192^  PHE_A^516^ SER_A^519^ SER_A^520^ ILE_A^521^ VAL_A^357^  LEU_A^360^ THR_A^364^ GLN_A^365^ HIS_A^509^ |
| BINDING POCKET 3 | ALA_A^452^ THR_A^453^ MET_A^456^ PRO_A^480^ GLU_A^481^  TRP_A^449^ GLU_A^478^ ARG_A^479^ ARG_A^351^ TYR_A^454^  TYR_A^459^ PRO_A^460^ TYR_A^475^ MET_A^477^ ILE_A^462^  ASN_A^355^ ALA_A^356^ VAL_A^525^ GLY_A^482^ LEU_A^529^  CYS_A^483^ PRO_A^484^ VAL_A^487^ ILE_A^521^ LEU_A^359^  ILE_A^451^ LEU_A^360^ LEU_A^448^ ALA_A^363^ PHE_A^512^ |
| BINDING POCKET 4 | GLU_A^274^ THR_A^291^ LEU_A^292^ LYS_A^293^ GLU_A^298^  PHE_A^302^ GLY_A^273^ LYS_A^290^ TYR_A^272^ GLU_A^305^  GLU_A^301^ PHE_A^401^ GLY_A^402^ ARG_A^381^ LEU_A^403^  LYS_A^304^ SER_A^404^ VAL_A^308^ ASP_A^400^ ARG_A^405^ PHE_A^378^ THR_A^296^ GLU_A^300^ LEU_A^303^ VAL_A^275^  HIS_A^415^ TYR_A^412^ ALA_A^418^ GLN_A^271^ ALA_A^414^  ASN_A^382^ THR_A^413^ GLY_A^270^ ALA_A^416^ HIS_A^380^  LYS_A^419^ PHE_A^420^ ASN_A^387^ PHE_A^435^ PRO_A^421^  ARG_A^386^ THR_A^425^ LEU_A^383^ ALA_A^384^ ILE_A^422^  ASP_A^440^ SER_A^439^ TRP_A^424^ ALA_A^443^ ALA_A^426^  LYS_A^423^ TRP_A^442^ |
| BINDING POCKET 5 | LYS_A^238^ ARG_A^239^ ASN_A^393^ PRO_A^237^ ASN_A^240^  TYR_A^158^ LEU_A^159^ HIS_A^394^ TYR_A^361^ ALA_A^236^  SER_A^162^ GLU_A^353^ SER_A^161^ VAL_A^358^ GLY_A^163^  TYR_A^234^ SER_A^167^ LEU_A^160^ VAL_A^354^ GLU_A^157^  GLN_A^352^ ASN_A^355^ LEU_A^359^ LEU_A^360^ ALA_A^363^  LEU_A^448^ ILE_A^451^ ALA_A^452^ PHE_A^512^ VAL_A^487^  ILE_A^521^ THR_A^364^ ALA_A^356^ VAL_A^357^ CYS_A^483^  ARG_A^185^ VAL_A^190^ ARG_A^351^ THR_A^453^ TYR_A^454^  MET_A^456^ PRO_A^484^ VAL_A^525^ SER_A^522^ GLY_A^188^  TYR_A^459^ PRO_A^460^ ILE_A^462^ TYR_A^475^ MET_A^477^  TRP_A^449^ GLU_A^478^ PRO_A^480^ GLU_A^481^ SER_A^520^  ARG_A^189^ GLY_A^461^ ARG_A^476^ ARG_A^479^ GLY_A^482^  HIS_A^192^ LEU_A^529^ ASP_A^523^ TYR_A^191^ ARG_A^153^  SER_A^519^ GLU_A^518^ PHE_A^516^ GLN_A^517^ GLU_A^526^  GLU_A^187^ |

**Table S11:** Active binding site residues of myc

| BINDING POCKET 1 | PHE_C^921^ PHE_C^922^ ALA_C^937^ LYS_C^939^ ILE_C^942^ ARG_C^914^ LEU_C^917^ LYS_C^918^ GLU_C^935^ PRO_C^938^ ARG_C^925^ ASN_C^934^ LYS_C^936^ |
| --- | --- |
| BINDING POCKET 2 | LEU_C^924^ GLN_C^927^ ILE_C^928^ ALA_C^946^ TYR_C^949^ ILE_C^950^ VAL_C^953^ GLN_C^954^ GLU_C^957^ GLU_C^956^ LEU_C^960^ LYS_C^945^ THR_C^947^ PRO_C^929^ ASP_C^926^ ALA_C^948^ |
| BINDING POCKET 3 | ARG_C^968^ LYS_C^969^ ARG_C^971^ GLU_C^972^ |
| BINDING POCKET 4 | ILE_C^928^ GLU_C^930^ LYS_C^945^ ALA_C^948^ TYR_C^949^ PRO_C^929^ SER_C^952^ ALA_C^946^ LYS_C^944^ |
| BINDING POCKET 5 | ARG_C^971^ LEU_C^974^ LYS_C^975^ |

**Table S12:** Active binding site residues of max

| BINDING POCKET 1 | LYS_9^89^ ASN_9^92^ ALA_9^93^ GLU_9^96^ GLN_9^112^ ASN_9^114^ LEU_9^111^ ARG_9^100^ THR_9^113^ SER_9^140^ GLY_9^137^ GLY_9^136^ THR_9^130^ LEU_9^122^ PRO_9^116^ ALA_9^133^ SER_9^138^ GLU_9^103^ SER_9^141^ VAL_9^99^ ILE_9^131^ SER_9^142^ PHE_9^134^ ASP_9^135^ SER_9^121^ SER_9^129^ ALA_9^126^ TYR_9^123^ THR_9^124^ SER_9^117^ SER_9^108^ ALA_9^109^ ALA_9^101^ LEU_9^102^ LYS_9^104^ SER_9^107^ GLN_9^98^ ARG_9^106^ LEU_9^95^ ALA_9^105^ GLN_9^110^ SER_9^118^ ASN_9^125^ SER_9^144^ GLU_9^145^ |
| --- | --- |
| BINDING POCKET 2 | SER_9^2^ ASP_9^12^ GLN_9^19^ ASP_9^23^ ALA_9^26^ HIS_9^27^ ALA_9^30^ MET_9^1^ ASP_9^3^ ASN_9^4^ ASP_9^5^ GLU_9^10^ GLN_9^15^ PRO_9^16^ SER_9^11^ GLU_9^8^ GLU_9^13^ ASP_9^6^ VAL_9^9^ ILE_9^7^ |
| BINDING POCKET 3 | LYS_9^57^ ALA_9^58^ SER_9^59^ ARG_9^60^ LYS_9^40^ ILE_9^63^ PHE_9^43^ ARG_9^47^ HIS_9^44^ |
| BINDING POCKET 4 | LEU_9^122^ ASN_9^125^ ALA_9^126^ ALA_9^133^ PHE_9^134^ ASP_9^135^ GLY_9^136^ LYS_9^127^ SER_9^132^ SER_9^117^ SER_9^140^ SER_9^141^ SER_9^142^ GLU_9^145^ GLU_9^147^ TYR_9^123^ THR_9^124^ SER_9^144^ ASN_9^120^ SER_9^121^ |
| BINDING POCKET 5 | ARG_9^152^ LYS_9^154^ SER_9^151^ LYS_9^153^ ARG_9^156^ PRO_9^149^ GLN_9^150^ GLU_9^158^ GLU_9^147^ GLU_9^148^ ALA_9^159^ ASP_9^119^ MET_9^157^ SER_9^160^ ASN_9^120^ GLU_9^145^ PRO_9^146^ SER_9^117^ SER_9^118^ SER_9^144^ GLN_9^110^ TYR_9^115^ LEU_9^122^ SER_9^121^ SER_9^142^ |

**Table S13:** Active binding site residues of myb

| BINDING POCKET 1 | GLU_9^292^ LEU_9^569^ THR_9^570^ ILE_9^295^ LEU_9^300^ PRO_9^358^ PHE_9^589^ LYS_9^587^ ASN_9^567^ LEU_9^301^ LEU_9^302^ GLU_9^297^ SER_9^571^ ALA_9^276^ LEU_9^357^ LEU_9^586^ ILE_9^568^ PRO_9^275^ LEU_9^298^ LYS_9^296^ PRO_9^566^ GLN_9^274^ ALA_9^565^ MET_9^303^ ALA_9^278^ ALA_9^588^ PRO_9^355^ GLU_9^582^ ASP_9^583^ GLN_9^281^ ALA_9^279^ ILE_9^280^ ARG_9^294^ THR_9^354^ ALA_9^359^ ASP_9^360^ SER_9^579^ ILE_9^269^ VAL_9^270^ ARG_9^282^ THR_9^590^ HIS_9^352^ SER_9^353^ MET_9^575^ PRO_9^577^ ASP_9^27^ ASN_9^271^ GLU_9^580^ ALA_9^576^ ASP_9^564^ VAL_9^267^ GLY_9^28^ LEU_9^487^ ALA_9^264^ MET_9^21^ HIS_9^351^ TYR_9^26^ LEU_9^29^ MET_9^486^ VAL_9^263^ SER_9^257^ PRO_9^31^ SER_9^256^ HIS_9^258^ ASN_9^268^ GLN_9^489^ GLY_9^362^ LEU_9^30^ ALA_9^563^ THR_9^490^ MET_9^221^ LYS_9^192^ LEU_9^364^ PRO_9^491^ VAL_9^193^ SER_9^363^ PRO_9^361^ ASN_9^285^ ASP_9^286^ LYS_9^293^ HIS_9^283^ LYS_9^291^ SER_9^572^ LEU_9^574^ GLU_9^299^ ALA_9^277^ ASN_9^584^ SER_9^356^ PRO_9^365^ SER_9^368^ PRO_9^561^ GLU_9^547^ VAL_9^562^ SER_9^304^ PRO_9^273^ ALA_9^578^ PRO_9^488^ ASP_9^18^ GLU_9^194^ LEU_9^348^ GLU_9^196^ GLN_9^195^ VAL_9^255^ VAL_9^259^ GLY_9^197^ |
| --- | --- |
| BINDING POCKET 2 | THR_9^405^ SER_9^406^ ASN_9^552^ THR_9^553^ MET_9^616^ GLY_9^482^ PRO_9^483^ PHE_9^556^ PRO_9^358^ ASN_9^567^ ILE_9^568^ LEU_9^569^ ALA_9^369^ SER_9^370^ THR_9^570^ ALA_9^359^ PRO_9^371^ ALA_9^372^ ARG_9^373^ VAL_9^386^ PRO_9^275^ ALA_9^276^ LEU_9^302^ LYS_9^587^ ILE_9^295^ GLU_9^297^ LEU_9^300^ PHE_9^589^ GLU_9^292^ LEU_9^301^ LEU_9^357^ GLN_9^274^ GLU_9^547^ ASP_9^549^ ASP_9^564^ PRO_9^566^ MET_9^575^ GLY_9^548^ GLN_9^558^ SER_9^407^ SER_9^550^ LEU_9^551^ TRP_9^608^ ALA_9^611^ TRP_9^546^ VAL_9^573^ GLN_9^602^ SER_9^612^ HIS_9^545^ SER_9^572^ ILE_9^9^ TYR_9^10^ HIS_9^436^ VAL_9^431^ THR_9^433^THR_9^432^ PHE_9^435^ ASP_9^341^ ALA_9^343^ PRO_9^434^ LYS_9^471^ HIS_9^472^ ALA_9^473^ HIS_9^409^ SER_9^342^ VAL_9^345^ GLU_9^350^ ASP_9^13^ GLU_9^14^ GLU_9^17^ ASN_9^411^ ASN_9^404^ GLU_9^410^ THR_9^607^ ASP_9^243^ PRO_9^344^ ASN_9^408^ SER_9^11^ SER_9^12^ SER_9^8^ THR_9^430^ HIS_9^339^ GLY_9^340^ THR_9^466^ HIS_9^335^ THR_9^336^ ARG_9^337^ SER_9^346^ LYS_9^428^ HIS_9^427^ LEU_9^429^ ASP_9^334^ GLY_9^426^ THR_9^331^ SER_9^412^ SER_9^606^ ILE_9^332^ ALA_9^333^ ILE_9^425^ CYS_9^604^ SER_9^605^ ASP_9^413^ LEU_9^414^ THR_9^422^ PRO_9^417^ GLY_9^349^ HIS_9^351^ PRO_9^488^ VAL_9^562^ ALA_9^563^ HIS_9^352^ ALA_9^565^ PRO_9^577^ ALA_9^576^ GLU_9^582^ VAL_9^193^ LEU_9^487^ THR_9^490^ PRO_9^355^ GLY_9^362^ SER_9^363^ GLN_9^489^ PRO_9^491^ SER_9^353^ THR_9^354^ LEU_9^364^ MET_9^486^ LEU_9^348^ LEU_9^484^ PRO_9^361^ SER_9^356^ LEU_9^494^ SER_9^571^ LEU_9^586^ SER_9^579^ ASP_9^583^ VAL_9^270^ MET_9^21^ ALA_9^578^ PRO_9^365^ SER_9^368^ PRO_9^238^ ASN_9^241^ MET_9^1^ LYS_9^92^ LEU_9^555^ LEU_9^574^ PRO_9^603^ CYS_9^347^ SER_9^401^ PRO_9^467^ GLU_9^618^ GLU_9^617^ ARG_9^457^ VAL_9^501^ PHE_9^540^ SER_9^560^ THR_9^559^ GLU_9^366^ LEU_9^498^ PRO_9^561^ ASP_9^497^ HIS_9^493^ VAL_9^495^ ASP_9^16^ LYS_9^32^ ILE_9^91^ GLN_9^237^ GLY_9^93^ PRO_9^94^ ARG_9^437^ ASP_9^438^ |
| BINDING POCKET 3 | PRO_9^233^ ASP_9^48^ ARG_9^45^ ASN_9^217^ SER_9^218^ TYR_9^10^ SER_9^11^ ASP_9^243^ TYR_9^244^ VAL_9^345^ CYS_9^347^ MET_9^1^ ASP_9^16^ ASN_9^242^ PHE_9^19^ LYS_9^32^ ALA_9^2^ ARG_9^42^ PRO_9^31^ SER_9^245^ LEU_9^348^ ASN_9^254^ GLN_9^253^ TYR_9^246^ PHE_9^214^ SER_9^33^ LYS_9^35^ GLY_9^34^ ARG_9^3^ LYS_9^216^ TRP_9^43^ LYS_9^84^ GLN_9^215^ LEU_9^232^ ARG_9^81^ VAL_9^240^ THR_9^44^ THR_9^239^ VAL_9^85^ LEU_9^38^ ARG_9^4^ SER_9^257^ VAL_9^259^ ARG_9^36^ HIS_9^258^ PRO_9^260^ GLN_9^77^ ARG_9^73^ ALA_9^234^ GLU_9^251^ALA_9^252^ LYS_9^40^ THR_9^41^ GLN_9^231^ HIS_9^80^ GLU_9^46^ TYR_9^247^ ALA_9^230^ THR_9^212^ PRO_9^228^ GLU_9^132^ ASN_9^179^ ARG_9^165^ LEU_9^220^ MET_9^221^ VAL_9^76^ THR_9^235^ GLY_9^236^ ARG_9^131^ ASP_9^178^ HIS_9^219^ ASN_9^136^ ALA_9^167^ HIS_9^135^ ARG_9^133^ GLN_9^237^ HIS_9^248^ GLU_9^478^ GLN_9^129^ ALA_9^170^ LYS_9^171^ ILE_9^249^ ILE_9^479^ THR_9^177^ GLU_9^168^ ARG_9^176^ TRP_9^134^ ASN_9^139^ LEU_9^138^ VAL_9^142^ PRO_9^112^ GLU_9^141^ PRO_9^174^ GLY_9^175^ LEU_9^173^ LYS_9^113^ SER_9^12^ SER_9^346^ MET_9^486^ LEU_9^484^ PRO_9^5^ SER_9^256^ LEU_9^30^ LYS_9^52^ GLU_9^49^ LEU_9^51^ HIS_9^37^ PRO_9^227^ GLY_9^39^ |
| BINDING POCKET 4 | ARG_9^465^ ALA_9^476^ SER_9^509^ GLY_9^510^ GLN_9^554^ SER_9^506^ THR_9^557^ ALA_9^473^ ALA_9^475^ GLU_9^514^ LEU_9^555^ ILE_9^479^ PHE_9^556^ GLY_9^482^ HIS_9^472^ LEU_9^474^ LYS_9^442^ ILE_9^502^ PRO_9^483^ LYS_9^480^ TYR_9^481^ LYS_9^503^ GLN_9^499^ ASN_9^183^ SER_9^187^ ASP_9^507^ HIS_9^248^ ALA_9^180^ ARG_9^190^ LYS_9^485^ HIS_9^184^ SER_9^250^ ILE_9^249^ SER_9^146^ LYS_9^144^ THR_9^145^ LYS_9^143^ TRP_9^147^ ARG_9^176^ ASN_9^186^ GLN_9^253^ THR_9^177^ TYR_9^247^ HIS_9^137^ VAL_9^142^ GLN_9^477^ ASN_9^136^ GLU_9^151^ LYS_9^445^ LYS_9^182^ GLU_9^251^ ALA_9^252^ ILE_9^511^ ALA_9^211^ VAL_9^255^ VAL_9^210^ TRP_9^95^ THR_9^212^ ARG_9^165^ THR_9^440^ VAL_9^441^ ALA_9^230^ ASN_9^179^ LYS_9^97^ ASP_9^100^ GLN_9^444^ THR_9^229^ ASP_9^178^ GLN_9^225^ THR_9^96^ THR_9^443^ PRO_9^140^ GLN_9^101^ TRP_9^166^ ILE_9^104^ |
| BINDING POCKET 5 | PRO_9^464^ SER_9^463^ PRO_9^467^ GLU_9^618^ ILE_9^399^ ALA_9^393^ GLU_9^394^ THR_9^534^ GLU_9^531^ LEU_9^396^ THR_9^395^ SER_9^532^ PRO_9^533^ LEU_9^460^ PHE_9^392^ VAL_9^530^ GLN_9^528^ LEU_9^522^ ASN_9^518^ ILE_9^459^ GLY_9^519^ SER_9^462^ GLN_9^397^ PHE_9^398^ PRO_9^521^ PRO_9^520^ THR_9^468^ LYS_9^387^ PHE_9^541^ HIS_9^545^  GLY_9^614^ GLN_9^619^ MET_9^616^ LEU_9^551^ GLN_9^516^ CYS_9^613^ ASN_9^552^ SER_9^612^ GLU_9^461^ SER_9^537^ GLU_9^391^ SER_9^458^ ILE_9^455^ LEU_9^523^ GLU_9^517^ GLY_9^538^ HIS_9^544^ PHE_9^540^ VAL_9^512^ GLY_9^548^ ALA_9^513^ SER_9^550^ THR_9^553^ PHE_9^515^ GLU_9^505^ GLN_9^554^ SER_9^509^ GLU_9^547^ GLN_9^558^ ARG_9^457^ THR_9^466^ GLU_9^508^ ASP_9^549^ THR_9^557^ PHE_9^556^ |

**Table S14:** Active binding site residues of pcna

| BINDING POCKET 1 | VAL_A^123^ GLN_A^125^ GLN_A^38^ GLU_A^124^ ASP_A^29^ CYS_A^27^ TRP_A^28^ ASN_A^36^ LEU_A^37^ SER_A^39^ MET_A^40^ LEU_A^126^ LEU_A^47^ ALA_A^67^ ASP_A^122^ HIS_A^44^ ALA_A^26^ LEU_A^121^ GLU_A^25^ ASP_A^41^ ILE_A^23^ ASN_A^24^ SER_A^42^ GLN_A^49^ LEU_A^50^ THR_A^51^ HIS_A^246^ GLY_A^127^ LEU_A^66^ MET_A^68^ LEU_A^22^ |
| --- | --- |
| BINDING POCKET 2 | GLN_A^38^ LEU_A^47^ GLN_A^49^ ILE_A^128^ LYS_A^248^ TYR_A^250^ GLU_A^132^ GLU_A^198^ TYR_A^133^ VAL_A^136^ ASN_A^200^ GLN_A^131^ SER_A^228^ MET_A^199^ SER_A^134^ PRO_A^129^ GLU_A^130^ VAL_A^236^ GLU_A^238^ LYS_A^240^ THR_A^226^ HIS_A^246^ |
| BINDING POCKET 3 | LEU_A^6^ GLY_A^56^ ILE_A^11^ ALA_A^242^ ASP_A^243^ MET_A^244^ ILE_A^241^ GLN_A^8^ LEU_A^221^ LYS_A^240^ SER_A^222^ PRO_A^220^ LYS_A^14^ SER_A^10^ |
| BINDING POCKET 4 | GLY_A^155^ ASP_A^156^ ALA_A^157^ |
| BINDING POCKET 5 | SER_A^10^ LYS_A^13^ LYS_A^14^ ASN_A^84^ |

**Table S15:** Active binding site residues of top3a

| BINDING POCKET 1 | LYS_A^409^ SER_A^410^ GLU_A^366^ ASP_A^411^ GLN_A^412^  LYS_A^593^ GLU_A^528^ ASP_A^328^ ASN_A^368^ VAL_A^330^  ASP_A^597^ THR_A^329^ THR_A^365^ ALA_A^413^ THR_A^534^  LYS_A^529^ GLY_A^531^ HIS_A^538^ CYS_A^596^ ILE_A^532^  ASP_A^535^ GLU_A^589^ ALA_A^590^ ARG_A^151^ ARG_A^177^  HIS_A^414^ ALA_A^536^ ARG_A^364^ ASP_A^197^ ALA_A^586^  VAL_A^200^ CYS_A^149^ ILE_A^595^ ASP_A^150^ LEU_A^592^  GLU_A^154^ TYR_A^362^ ARG_A^175^ THR_A^537^ ARG_A^585^  GLU_A^152^ ARG_A^207^ GLU_A^333^ MET_A^348^ ALA_A^539^  GLU_A^540^ LEU_A^588^ HIS_A^541^ VAL_A^204^ ALA_A^203^  LYS_A^345^ CYS_A^244^ GLU_A^352^ GLU_A^41^ ASP_A^148^  ARG_A^349^ ASP_A^211^ GLU_A^346^ ILE_A^156^ GLU_A^543^  HIS_A^92^ GLY_A^91^ THR_A^367^ ILE_A^369^ PHE_A^370^  PRO_A^371^ PRO_A^415^ ARG_A^372^ SER_A^179^ LEU_A^374^  ASP_A^373^ GLU_A^180^ PHE_A^178^ ALA_A^185^ HIS_A^184^  ASN_A^375^ THR_A^188^ THR_A^182^ LEU_A^379^ HIS_A^418^  ALA_A^189^ THR_A^420^ GLN_A^382^ ASN_A^192^ VAL_A^378^  GLU_A^195^ ASN_A^408^ HIS_A^530^ |
| --- | --- |
| BINDING POCKET 2 | SER_A^179^ PRO_A^371^ ARG_A^372^ GLN_A^412^ ILE_A^369^  SER_A^410^ PHE_A^370^ ASP_A^373^ LEU_A^374^ ASN_A^375^  LEU_A^376^ THR_A^377^ PRO_A^404^ PRO_A^402^ ASN_A^406^  HIS_A^184^ ALA_A^185^ THR_A^188^ PRO_A^183^ THR_A^182^  GLN_A^382^ GLU_A^180^ THR_A^420^ LEU_A^379^ THR_A^367^  HIS_A^414^ PRO_A^415^ ALA_A^413^ ASP_A^411^ VAL_A^378^  HIS_A^418^ PRO_A^419^ LYS_A^421^ ASN_A^192^ ARG_A^177^  PHE_A^178^ ALA_A^189^ CYS_A^149^ |
| BINDING POCKET 3 | ASP_A^150^ ARG_A^364^ ASP_A^535^ ALA_A^536^ ARG_A^151^  GLU_A^152^ THR_A^537^ GLU_A^540^ TYR_A^362^ GLU_A^352^  ARG_A^207^ GLU_A^41^ ASP_A^148^ ILE_A^156^ HIS_A^92^  THR_A^534^ HIS_A^541^ ASP_A^211^ ARG_A^585^ ALA_A^539^  GLU_A^333^ MET_A^348^ GLY_A^91^ LYS_A^345^ GLU_A^589^  LEU_A^592^ ALA_A^203^ VAL_A^204^ ARG_A^349^ LYS_A^42^  HIS_A^96^ GLN_A^208^ SER_A^90^ LEU_A^94^ THR_A^544^  GLU_A^346^ LYS_A^353^ GLY_A^242^ SER_A^243^ CYS_A^244^  GLN_A^245^ TYR_A^241^ GLY_A^215^ LEU_A^212^ ALA_A^95^  ILE_A^214^ ARG_A^548^ ALA_A^216^ THR_A^219^ PHE_A^126^  SER_A^240^ LEU_A^238^ PRO_A^123^ TRP_A^105^ ARG_A^220^  ASN_A^125^ ASP_A^97^ PHE_A^98^ TYR_A^121^ GLU_A^543^ |
| BINDING POCKET 4 | ASP_A^150^ ARG_A^151^ GLU_A^154^ VAL_A^200^ CYS_A^149^ ARG_A^177^ ALA_A^413^ ASP_A^411^ CYS_A^596^ ASP_A^597^  LYS_A^593^ ASP_A^197^ ASP_A^535^ ARG_A^364^ HIS_A^414^  THR_A^194^ GLU_A^195^ ARG_A^175^ PRO_A^196^ GLN_A^412^  ASN_A^192^ PHE_A^178^ SER_A^179^ GLN_A^198^ ARG_A^199^ |
| BINDING POCKET 5 | ASP_A^211^ SER_A^243^ CYS_A^244^ GLN_A^245^ HIS_A^541^  ARG_A^585^ GLY_A^242^ ARG_A^207^ GLU_A^540^ THR_A^544^  TYR_A^241^ LEU_A^212^ GLY_A^215^ ARG_A^548^ ALA_A^216^  THR_A^219^ SER_A^240^ HIS_A^96^ LEU_A^238^ PHE_A^98^  ARG_A^220^ TRP_A^105^ |

**Table S16:** Active binding site residues of p73 using three PDB IDs.

| PDB ID: 2WQI | BINDING POCKET 1 | LEU_A^357^ GLN_A^358^ VAL_A^359^ LEU_A^368^ PHE_A^365^ ASN_A^364^ MET_A^369^ TYR_A^356^ LEU_A^371^ GLU_A^366^ LYS_A^372^ ILE_A^367^ LEU_A^375^ TYR_A^355^ GLU_A^373^ THR_A^354^ GLU_A^376^ ASP_A^353^ GLU_A^352^ GLU_A^379^ |
| --- | --- | --- |
|  | BINDING POCKET 2 | LEU_A^368^ MET_A^369^ LYS_A^372^ LYS_A^370^ GLU_A^373^ |
|  | BINDING POCKET 3 | GLN_A^358^ VAL_A^359^ ARG_A^360^ |
|  | BINDING POCKET 4 | MET_A^378^ VAL_A^386^ ARG_A^390^ LEU_A^375^ GLU_A^379^ |
|  | BINDING POCKET 5 | VAL_A^381^ LEU_A^385^ TYR_A^389^ MET_A^378^ VAL_A^386^ SER_A^388^ GLN_A^392^ ARG_A^390^ LEU_A^375^ SER_A^374^ GLU_A^379^ LEU_A^377^ GLN_A^383^ LEU_A^380^ PRO_A^382^ |
| PDB ID : 2XWC | BINDING POCKET 1 | ARG_A^268^ ARG_A^300^ ASP_A^301^ ALA_A^304^ ASP_A^305^ HIS_A^308^ ARG_A^293^ ILE_A^294^ CYS_A^295^ PRO_A^270^ GLU_A^291^ SER_A^260^ PRO_A^298^ ARG_A^269^ SER_A^261^ VAL_A^263^ ILE_A^271^ LEU_A^214^ PHE_A^256^ ILE_A^273^ GLY_A^292^ CYS_A^258^ ASN_A^259^ VAL_A^191^ LYS_A^192^ ARG_A^193^ CYS_A^194^ TYR_A^181^ MET_A^257^ ILE_A^215^ PHE_A^290^ PRO_A^179^  CYS_A^262^ GLY_A^264^ VAL_A^190^ PRO_A^195^ ASP_A^189^ |
|  | BINDING POCKET 2 | ILE_A^273^ ILE_A^274^ PHE_A^290^ |
|  | BINDING POCKET 3 | LEU_A^148^ LYS_A^149^ LYS_A^182^ LEU_A^272^ SER_A^289^ PHE_A^290^ GLU_A^291^ ASN_A^118^ THR_A^119^ ASP_A^120^ LYS_A^150^ VAL_A^129^ PHE_A^131^ TYR_A^144^ ARG_A^288^ GLU_A^128^ THR_A^130^ |
|  | BINDING POCKET 4 | GLY_A^172^ PRO_A^239^ THR_A^173^ LEU_A^222^ VAL_A^238^ GLN_A^224^ ALA_A^174^ TYR_A^240^ SER_A^223^ VAL_A^237^ ARG_A^280^ PRO_A^171^ ILE_A^175^ MET_A^279^ ASP_A^281^ GLY_A^282^ GLU_A^278^ ARG_A^176^ VAL_A^226^ GLN_A^283^ |
|  | BINDING POCKET 5 | PHE_A^203^ GLU_A^205^ GLN_A^207^ ARG_A^201^ ASP_A^202^ ASN_A^204^ ALA_A^156^ ARG_A^216^ GLU_A^218^ ASN_A^255^ MET_A^257^ ARG_A^193^ ALA_A^209^ PRO_A^210^ ALA_A^211^ SER_A^208^ HIS_A^213^ ILE_A^215^ TYR_A^225^ |
| PDB ID : 1DXS | BINDING POCKET 1 | GLU_A^21^ SER_A^25^ TYR_A^22^ GLN_A^26^ ALA_A^44^ LYS_A^46^ LEU_A^45^ ASN_A^18^ |
|  | BINDING POCKET 2 | LEU_A^7^ VAL_A^8^ GLN_A^29^ GLY_A^27^ LEU_A^28^ SER_A^30^ HIS_A^33^ GLN_A^26^ LEU_A^34^ PHE_A^23^ LEU_A^37^ ILE_A^31^ |
|  | BINDING POCKET 3 | LEU_A^34^ GLN_A^35^ ASN_A^36^ LEU_A^37^ LEU_A^59^ GLN_A^60^ THR_A^38^ TRP_A^56^ ILE_A^39^ LEU_A^42^ HIS_A^33^ LEU_A^62^ ASP_A^41^ |
|  | BINDING POCKET 4 | LEU_A^45^ |
|  | BINDING POCKET 5 | PHE_A^10^ LEU_A^14^ |

**Table S17:** Active binding site residues of blm using two PDB IDs

| PDB ID: 4O3M | BINDING POCKET 1 | LEU_A^716^ HIS_A^798^ MET_A^951^ GLU_A^796^ GLY_A^952^ GLY_A^950^ LEU_A^719^ PRO_A^715^ GLN_A^975^ ILE_A^953^ ARG_A^982^ THR_A^832^ ARG_A^979^ ASP_A^954^ THR_A^691^ ALA_A^831^ THR_A^830^ PHE_A^949^ GLU_A^976^ ILE_A^947^ ARG_A^859^ TYR_A^974^ ALA_A^833^ VAL_A^837^ LYS_A^695^ GLY_A^978^ ASP_A^795^ GLU_A^971^ GLN_A^723^ GLY_A^692^ SER_A^696^ GLY_A^972^ ASN_A^834^ PHE_A^1045^ ALA_A^797^ TYR_A^811^ LYS_A^726^ GLY_A^693^ GLY_A^694^ PRO_A^956^ GLN_A^700^ ASP_A^983^ LEU_A^697^ ARG_A^669^ ASP_A^722^ LYS_A^955^ PHE_A^1238^ ASN_A^1239^ GLN_A^672^ ASN_A^1242^ LEU_A^665^ LEU_A^730^ VAL_A^1244^ PHE_A^663^ GLY_A^984^ LYS_A^1270^ GLY_A^1265^ ASN_A^667^ ASP_A^1264^ PHE_A^1241^ THR_A^1245^ SER_A^729^ LYS_A^1248^ ASN_A^861^ GLY_A^981^ PRO_A^690^ HIS_A^860^ MET_A^855^ LEU_A^862^ ASN_A^858^ THR_A^670^ ALA_A^980^ SER_A^856^ PHE_A^857^ SER_A^969^ ILE_A^841^ ARG_A^808^ GLN_A^809^ ASP_A^810^ ARG_A^813^ PRO_A^767^ THR_A^1015^ ARG_A^1016^ THR_A^1018^ HIS_A^1019^ HIS_A^1014^ GLU_A^1017^ ALA_A^948^ GLN_A^844^ LYS_A^968^ ASN_A^1022^ ASP_A^840^ THR_A^946^ TYR_A^894^ PRO_A^967^ LEU_A^966^ ARG_A^836^ TYR_A^1044^ SER_A^718^ LEU_A^829^ LEU_A^727^ THR_A^728^ THR_A^1243^ MET_A^689^ GLY_A^664^ PHE_A^659^ PHE_A^668^ GLU_A^985^ HIS_A^666^ |
| --- | --- | --- |
|  | BINDING POCKET 2 | LYS_A^1207^ ASP_A^938^ TRP_A^881^ ASN_A^1194^ ASP_A^1064^ SER_A^1062^ CYS_A^1063^ GLU_A^1193^ LEU_A^867^ LYS_A^1068^ GLU_A^1213^ MET_A^1214^ VAL_A^1215^ LYS_A^1217^ LYS_A^1216^ TYR_A^888^ LEU_A^1219^ GLY_A^1220^ THR_A^1223^ ASP_A^889^ SER_A^890^ GLN_A^937^ THR_A^1243^ ASP_A^957^ GLU_A^1224^ LYS_A^1227^ ARG_A^959^ LYS_A^1247^ GLU_A^1212^ GLU_A^1251^ GLU_A^985^ HIS_A^886^ ILE_A^986^ TYR_A^1237^ VAL_A^1244^ HIS_A^885^ PRO_A^887^ HIS_A^988^ GLN_A^941^ GLN_A^1210^ ARG_A^1211^ ASP_A^1060^ CYS_A^940^ LYS_A^863^ SER_A^1209^ ALA_A^914^ GLY_A^939^ TYR_A^865^ VAL_A^1208^ PRO_A^1059^ CYS_A^1067^ CYS_A^1066^ CYS_A^1055^ |
|  | BINDING POCKET 3 | GLN_A^975^ GLU_A^976^ GLY_A^978^ ARG_A^979^ |
|  | BINDING POCKET 4 | ARG_A^669^ VAL_A^1244^ ASP_A^1264^ LEU_A^665^ ASP_A^983^ ASN_A^1242^ ARG_A^982^ GLY_A^984^ LYS_A^726^ PRO_A^956^ THR_A^1245^ THR_A^1243^ GLY_A^1265^ PHE_A^663^ LEU_A^730^ LYS_A^1270^ GLY_A^664^ VAL_A^1266^ THR_A^1267^ PHE_A^1241^ ASP_A^957^ PHE_A^1238^ TYR_A^1237^ SER_A^696^ GLN_A^723^ ASP_A^954^ LEU_A^719^ ASP_A^722^ ASN_A^1239^ SER_A^718^ GLN_A^672^ GLY_A^694^ LYS_A^695^ LEU_A^697^ GLN_A^700^ SER_A^729^ GLY_A^693^ ASN_A^667^ PHE_A^659^ PHE_A^668^ HIS_A^666^ GLY_A^692^ LYS_A^955^ THR_A^691^ ALA_A^831^ GLU_A^796^ ASP_A^795^ |
|  | BINDING POCKET 5 | ILE_A^1033^ ARG_A^1037^ LEU_A^990^ HIS_A^885^ ASN_A^1032^ ILE_A^986^ LYS_A^1217^ ASP_A^1064^ TYR_A^865^ HIS_A^988^ LEU_A^1004^ MET_A^1007^ GLU_A^1008^ ARG_A^898^ ALA_A^920^ GLY_A^921^ THR_A^946^ SER_A^897^ ILE_A^1005^ LYS_A^968^ ALA_A^948^ ASN_A^1164^ ASP_A^1165^ GLN_A^1166^ ARG_A^1003^ ARG_A^899^ LEU_A^896^ ILE_A^947^ HIS_A^919^ ALA_A^1167^ ARG_A^1000^ GLU_A^900^ LEU_A^1001^ CYS_A^895^ TYR_A^894^ HIS_A^996^ ASN_A^1162^ ILE_A^1168^ THR_A^999^ THR_A^903^ ALA_A^1169^ LYS_A^872^ GLU_A^976^ THR_A^1110^ PRO_A^871^ ASP_A^997^ MET_A^904^ LEU_A^966^ PRO_A^967^ LYS_A^869^ ALA_A^875^ THR_A^907^ VAL_A^874^ ALA_A^964^ SER_A^965^ MET_A^1113^ ASN_A^1112^ MET_A^1111^ GLU_A^1157^ LYS_A^873^ PHE_A^876^ VAL_A^1171^ TYR_A^995^ LYS_A^870^ THR_A^994^ LYS_A^1147^ TYR_A^888^ GLN_A^937^ CYS_A^940^ GLN_A^941^ SER_A^890^ ASP_A^889^ LYS_A^1227^ GLU_A^1143^ GLU_A^1224^ THR_A^1223^ PRO_A^887^ GLY_A^1220^ TYR_A^993^ ASP_A^957^ TYR_A^1237^ PRO_A^868^ ARG_A^959^ THR_A^1243^ GLU_A^1031^ GLU_A^1221^ LEU_A^867^ ASP_A^877^ PHE_A^992^ CYS_A^1030^ LEU_A^1246^ HIS_A^1140^ ARG_A^1144^ GLU_A^880^ VAL_A^866^ TRP_A^881^ LEU_A^1219^ LYS_A^884^ HIS_A^886^ LYS_A^1216^ SER_A^987^ GLU_A^985^ LYS_A^1247^ THR_A^1192^ ASN_A^1065^ ARG_A^883^ LYS_A^1068^ LYS_A^863^ GLU_A^1213^ MET_A^1190^ ASN_A^1194^ GLY_A^984^ GLU_A^1191^ VAL_A^1215^ GLU_A^1193^ ASP_A^1060^ GLU_A^1212^ GLU_A^1251^ LYS_A^1207^ GLN_A^1210^ CYS_A^1063^ SER_A^1209^ SER_A^1062^ TYR_A^864^ VAL_A^1208^ PRO_A^1059^ ARG_A^1211^ VAL_A^1061^ CYS_A^1067^ CYS_A^1066^ CYS_A^1055^ LYS_A^1056^ |
| PDB ID: 5LUP | BINDING POCKET 1 | ILE_C^373^ HIS_C^374^ MET_C^376^ GLU_C^377^ HIS_C^378^ CYS_C^380^ ARG_C^407^ LEU_C^411^ ILE_C^379^ ILE_C^383^ LEU_C^400^ LEU_C^410^ ASP_C^384^ GLN_C^403^ ARG_C^404^ ILE_C^406^ |
|  | BINDING POCKET 2 | LYS_C^381^ ASP_C^384^ LEU_C^411^ ARG_C^404^ ARG_C^408^ |
|  | BINDING POCKET 3 | LEU_C^368^ GLN_C^369^ LEU_C^372^ |
|  | BINDING POCKET 4 | GLU_C^377^ ARG_C^407^ LEU_C^411^ GLN_C^403^ ILE_C^406^ LEU_C^410^ |
|  | BINDING POCKET 5 | ASP_C^384^ ARG_C^408^ LEU_C^411^ |

**Domain Analysis of target proteins under study using MOTIF Search and ScanProsite**


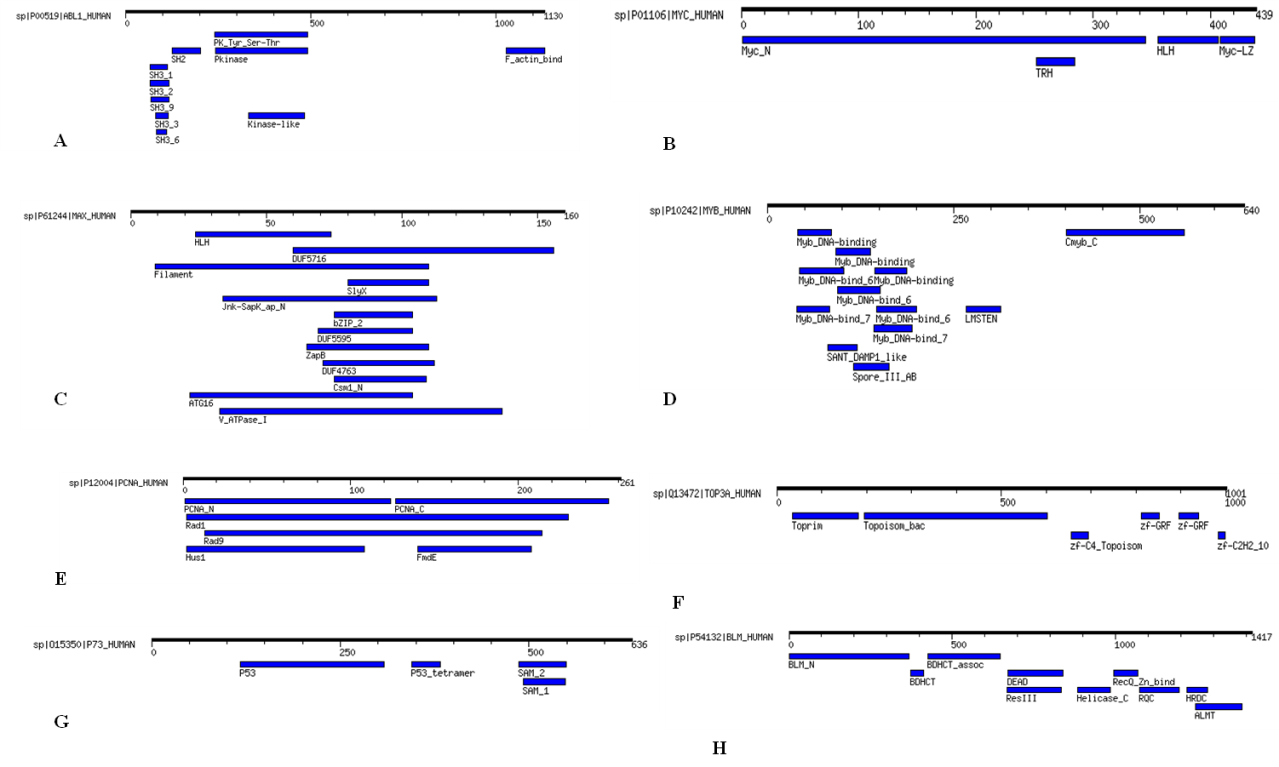


**Figure 2:** Graphical representation of domains of proteins- (A) abl1, (B) myc, (C) max, (D) myb, (E) pcna, (F) top3a, (G) p73, (H) blm. The output from both the tools used, gave a very similar domain analysis for a given protein.

**Table S18:** Domain analysis for ALL protein

**abl1**

| **DOMAIN ID** | **POSITION** | **DESCRIPTION** |
| --- | --- | --- |
| PK_Tyr_Ser-Thr | 242..492 | Protein tyrosine and serine/threonine kinase |
| Pkinase | 244..491 | Protein kinase domain |
| F_actin_bind | 1026..1130 | F-actin binding |
| SH2 | 127..202 | SH2 domain |
| SH3_1 | 67..113 | SH3 domain |
| SH3_2 | 66..118 | Variant SH3 domain |
| SH3_9 | 68..117 | Variant SH3 domain |
| SH3_3 | 82..116 | Bacterial SH3 domain |
| Kinase-like | 333..483 | Kinase-like |
| SH3_6 | 83..112 | SH3 domain (SH3b1 type) |

**myc**

| **DOMAIN ID** | **POSITION** | **DESCRIPTION** |
| --- | --- | --- |
| Myc_N | 1..345 | Myc amino-terminal region |
| Myc-LZ | 408..438 | Myc leucine zipper domain |
| HLH | 355..407 | Helix-loop-helix DNA-binding domain |
| TRH | 252..284 | Thyrotropin-releasing hormone (TRH) |

**max**

| **DOMAIN ID** | **POSITION** | **DESCRIPTION** |
| --- | --- | --- |
| HLH | 24..74 | Helix-loop-helix DNA-binding domain |
| DUF5716 | 60..156 | Family of unknown function (DUF5716) |
| Filament | 9..110 | Intermediate filament protein |
| SlyX | 80..110 | SlyX |
| Jnk-SapK_ap_N | 34..113 | JNK_SAPK-associated protein-1 |
| bZIP_2 | 75..104 | Basic region leucine zipper |
| DUF5595 | 69..104 | Domain of unknown function (DUF5595) |
| ZapB | 65..110 | Cell division protein ZapB |
| DUF4763 | 71..112 | Domain of unknown function (DUF4763) |
| Csm1_N | 75..109 | Csm1 N-terminal domain |
| ATG16 | 22..104 | Autophagy protein 16 (ATG16) |
| V_ATPase_I | 33..137 | V-type ATPase 116kDa subunit family |

**myb**

| **DOMAIN ID** | **POSITION** | **DESCRIPTION** |
| --- | --- | --- |
| Cmyb_C | 401..560 | C-myb, C-terminal |
| Myb_DNA-binding | 41..86,92..138,145..187 | Myb-like DNA-binding domain |
| Myb_DNA-bind_6 | 43..103,95..152,147..201 | Myb-like DNA-binding domain |
| LMSTEN | 267..313 | LMSTEN motif |
| Myb_DNA-bind_7 | 40..84,143..194 | Myb DNA-binding like |
| SANT_DAMP1_like | 81..121 | SANT/Myb-like domain of DAMP1 |
| Spore_III_AB | 116..164 | Stage III sporulation protein AB (spore_III_AB) |

**pcna**

| **DOMAIN ID** | **POSITION** | **DESCRIPTION** |
| --- | --- | --- |
| PCNA_C | 127..254 | Proliferating cell nuclear antigen, C-terminal domain |
| PCNA_N | 1..124 | Proliferating cell nuclear antigen, N-terminal domain |
| Rad1 | 2..230 | Repair protein Rad1/Rec1/Rad17 |
| Rad9 | 13..214 | Rad9 |
| Hus1 | 2..108 | Hus1-like protein |
| FmdE | 140..208 | FmdE, Molybdenum formylmethanofuran dehydrogenase operon |

**top3a**

| **DOMAIN ID** | **POSITION** | **DESCRIPTION** |
| --- | --- | --- |
| Topoisom_bac | 196..603 | DNA topoisomerase |
| zf-GRF | 811..851,896..939 | GRF zinc finger |
| Toprim | 36..181 | Toprim domain |
| zf-C4_Topoisom | 656..693 | Topoisomerase DNA binding C4 zinc finger |
| zf-C2H2_10 | 983..998 | C2H2 zinc-finger |

**p73**

| **DOMAIN ID** | **POSITION** | **DESCRIPTION** |
| --- | --- | --- |
| P53 | 118..308 | P53 DNA-binding domain |
| P53_tetramer | 345..383 | P53 tetramerisation motif |
| SAM_2 | 486..549 | SAM domain (Sterile alpha motif) |
| SAM_1 | 492..548 | SAM domain (Sterile alpha motif) |

**blm**

| **DOMAIN ID** | **POSITION** | **DESCRIPTION** |
| --- | --- | --- |
| BLM_N | 1..367 | N-terminal region of Bloom syndrome protein |
| BDHCT_assoc | 425..647 | BDHCT-box associated domain on Bloom syndrome protein |
| BDHCT | 372..411 | BDHCT (NUC031) domain |
| DEAD | 671..838 | DEAD/DEAH box helicase |
| RecQ_Zn_bind | 995..1067 | RecQ zinc-binding |
| RQC | 1072..1195 | RQC domain |
| Helicase_C | 883..983 | Helicase conserved C-terminal domain |
| HRDC | 1217..1281 | HRDC domain |
| ResIII | 667..833 | Type III restriction enzyme, res subunit |
| ALMT | 1245..1388 | Aluminium activated malate transporter |

**Distribution of Binding Affinity of Compound 88 with eight target proteins as predicted via AutoDock Vina based Virtual Screening**

**
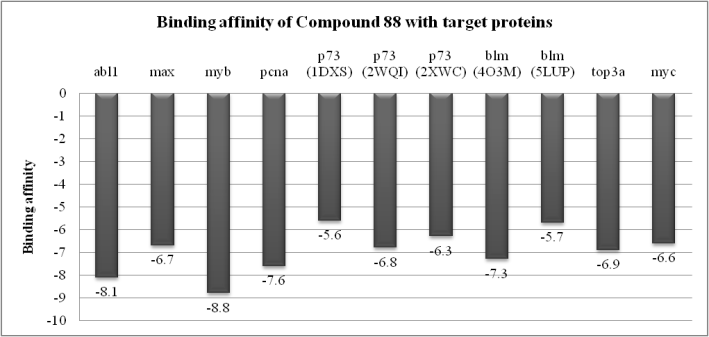
**

**Figure 3:** Distribution of binding affinity of Compound 88 with target proteins as predicted by AutoDock Vina; X-axis corresponds to target proteins; Y-axis corresponds to binding affinity

**3D Ligand Similarity Search (LS-align) - Compound 3 and ATRA**


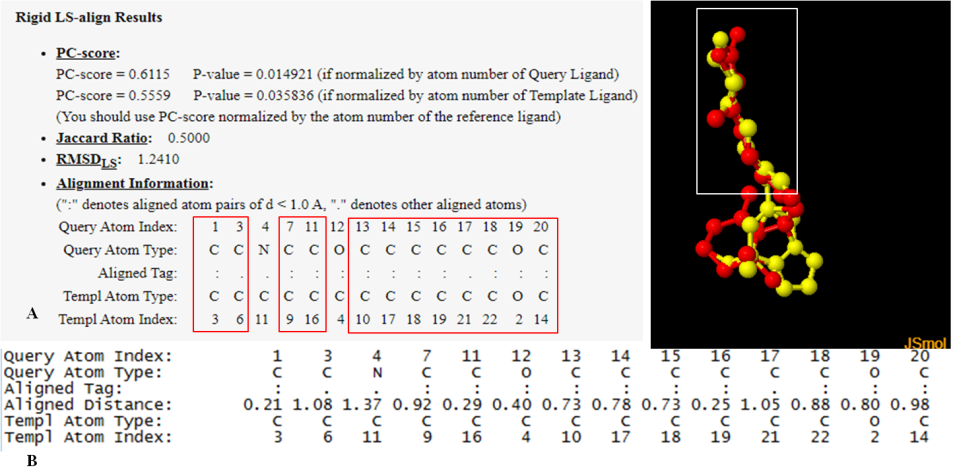


**Figure 4:** (A) The rigid align scores of query (Compound 3-yellow) and template (ATRA-red) ligands; the red box indicates toward strongly aligned atom pairs of the aligned distance <1Å; Query ligand is depicted in yellow and template ligand is depicted in red; the white box depicting the aligned region between compound 3 and ATRA; (B) The rigid align distance (Å) between query (Compound 3) and template (ATRA) ligand atom pairs. Compound 3 and ATRA were found to share approximately 85% atomic identity


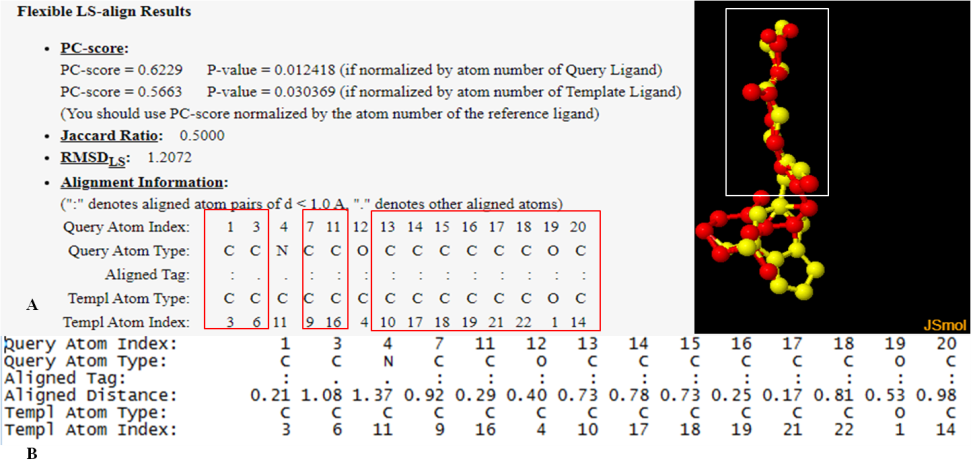


**Figure 5:** (A) The flexible align scores of query (Compound 3-yellow) and template (ATRA-red) ligands; the red box indicates toward strongly aligned atom pairs of the aligned distance <1Å; Query ligand is depicted in yellow and template ligand is depicted in red; the white box depicting the aligned region between compound 3 and ATRA; (B) The flexible align distance (Å) between query (Compound 3) and template (ATRA) ligand atom pairs. Compound 3 and ATRA were found to share approximately 85% atomic identity

**Molecular Docking of ATRA with myc and p73**

ATRA was docked with proteins myc and p73. We observed that the atoms of ATRA and Compound 3 involved in interactions with target proteins were similar (**Fig.6**).


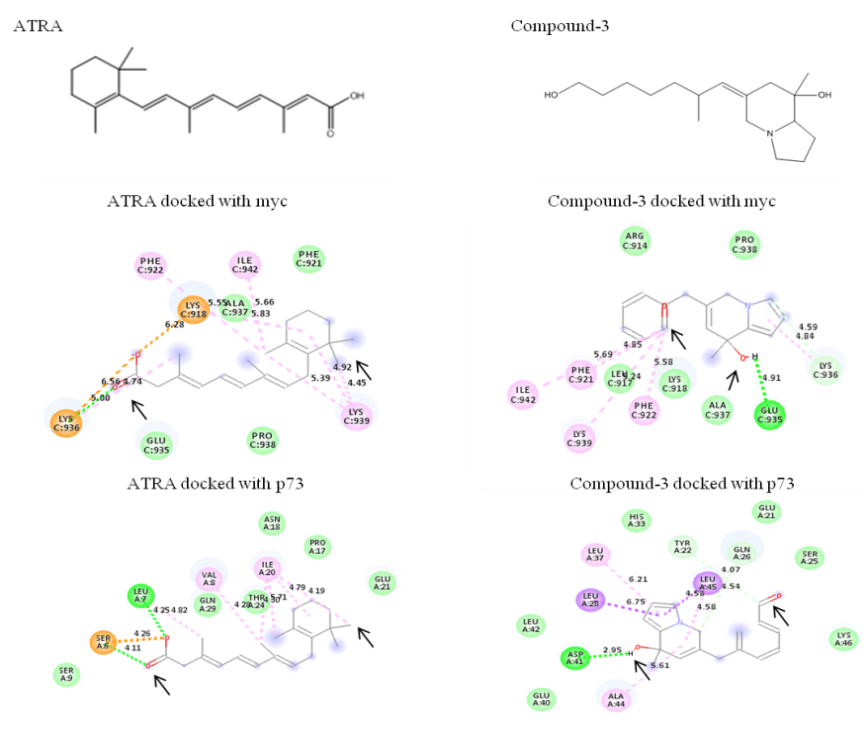


**Figure 6:** Structure of ATRA and Compound 3 and their docking with proteins myc and p73 respectively; the arrows indicate atoms involved in protein binding. The atoms of both the ligands had similar binding atoms with the respective protein, confirming similarity index between the two biomolecules

**Distribution of Binding Energy of UBS109 with eight target proteins as predicted via AutoDock Tools**


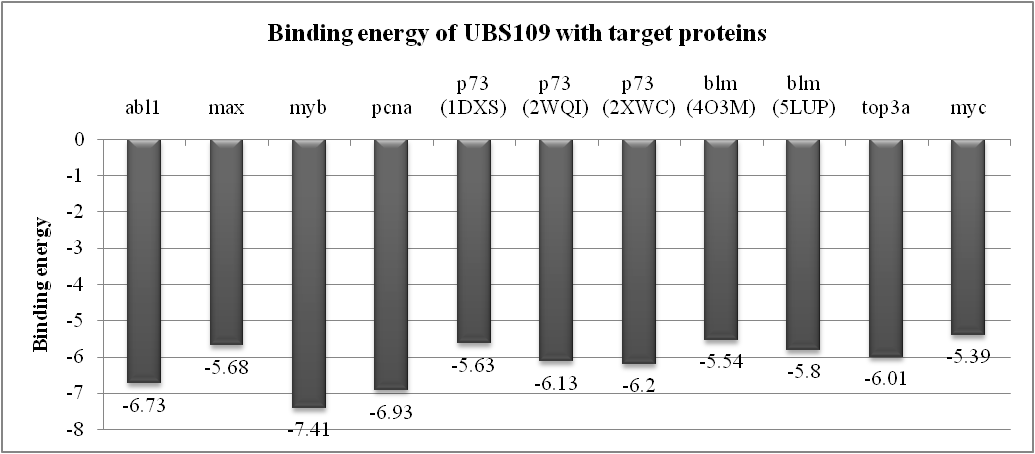


**Figure 7:** Distribution of binding energy of UBS109 with target proteins as predicted by AutoDock Tools; X-axis corresponds to target proteins; Y-axis corresponds to binding energy

**Detailed list of 536 Curcuma compounds**

**Table S19:** List of all 536 Curcuma compounds under study

| **Serial no.** | **Compound ID** | **Chemical name** |
| --- | --- | --- |
| 1 | 1a | 1-acryloyl-3,5-di((E)-benzylidene)piperidin-4-one |
| 2 | 1b | 1-acryloyl-3,5-bis((E)-4-chlorobenzylidene)piperidin-4-one |
| 3 | 1c | 1-acryloyl-3,5-bis((E)-3,4-dichlorobenzylidene)piperidin-4-one |
| 4 | 1d | 1-acryloyl-3,5-bis((E)-4-fluorobenzylidene)piperidin-4-one |
| 5 | 1e | 1-acryloyl-3,5-bis((E)-4-nitrobenzylidene)piperidin-4-one |
| 6 | 1f | 1-acryloyl-3,5-bis((E)-4-methoxybenzylidene)piperidin-4-one |
| 7 | 1g | 1-((3Z,5Z)-3,5-bis((4-(di(l3-methyl)amino)phenyl)methylene)-4-methylenepiperidin-1-yl)prop-2-en-1-one |
| 8 | A17 | (2E,2'E)-3,3'-(1,4-phenylene)bis(1-(2-bromophenyl)prop-2-en-1-one) |
| 9 | B5 | 2,5-bis((E)-4-hydroxy-3,5-dimethoxybenzylidene)cyclopentan-1-one |
| 10 | B19 | (1E,4E)-1-(2,3-dimethoxy-4-methylphenyl)-5-(2,3-dimethoxyphenyl)penta-1,4-dien-3-one |
| 11 | CA15 | 1-ethyl-3,5-bis((E)-3,4,5-trimethoxybenzylidene)piperidin-4-one |
| 12 | CN | ((1E,3Z,6E)-3-hydroxy-5-oxohepta-1,3,6-triene-1,7-diyl)bis(2-methoxy-4,1-phenylene) dinicotinate |
| 13 | Comp1 | 3,5-bis((E)-2,3-dimethoxybenzylidene)piperidin-4-one |
| 14 | comp1a | (1E,4E)-1,5-bis(2-fluorophenyl)penta-1,4-dien-3-one |
| 15 | comp1b | (1E,4Z,6E)-5-hydroxy-1,7-bis(3-hydroxyphenyl)hepta-1,4,6-trien-3-one |
| 16 | compa | 1-methyl-3,5-bis((E)-3,4,5-trimethoxybenzylidene)piperidin-4-one |
| 17 | cur3d | (Z)-2-((E)-1-hydroxy-3-(4-hydroxy-3-methylphenyl)allylidene)-5-((E)-4-hydroxy-3-methoxybenzylidene)cyclopentan-1-one |
| 18 | da0324 | 2-((E)-2,5-dimethoxybenzylidene)-5-((E)-4-hydroxy-3-methoxybenzylidene)cyclopentan-1-one |
| 19 | dimethyl CUR | (1E,4Z,6E)-5-hydroxy-1,7-bis(4-hydroxy-3-methoxyphenyl)-2,6-dimethylhepta-1,4,6-trien-3-one |
| 20 | DM-1 | sodium 4-((1E,4E)-5-(4-hydroxy-3-methoxyphenyl)-3-oxopenta-1,4-dien-1-yl)-2-methoxyphenolate |
| 21 | EF-243C | 1-methyl-3,5-bis((E)-2,4,5-trifluorobenzylidene)piperidin-4-one |
| 22 | EF24-3F | 1-ethyl-3,5-bis((E)-4-(pentafluoro-l6-sulfaneyl)benzylidene)piperidin-4-one |
| 23 | EF31 | (3E,5E)-3,5-bis(pyridin-2-ylmethylene)piperidin-4-one |
| 24 | GL63 | (1E,4E)-1,5-bis(2-bromophenyl)penta-1,4-dien-3-one |
| 25 | Go-y030 | (1E,4E)-1,5-bis(3,5-bis(methoxymethoxy)phenyl)penta-1,4-dien-3-one |
| 26 | Go-y078 | (1E,4E)-1-(4-hydroxy-3,5-dimethoxyphenyl)-5-(3,4,5-trimethoxyphenyl)penta-1,4-dien-3-one |
| 27 | H-4073 | 3,5-bis((E)-4-fluorobenzylidene)piperidin-4-one |
| 28 | HO3H67 | 3,5-bis((E)-4-fluorobenzylidene)-1-((1-hydroxy-2,2,5,5-tetramethyl-2,5-dihydro-1H-pyrrol-3-yl)methyl)piperidin-4-one |
| 29 | IHCH | (E)-2-((1H-indol-3-yl)methylene)-6-((E)-3-hydroxy-4-methoxybenzylidene)cyclohexan-1-one |
| 30 | L48H37 | 1-ethyl-3,5-bis((E)-2,3,4-trimethoxybenzylidene)piperidin-4-one |
| 31 | MC37 | (E)-1-(3'-fluoro-[1,1'-biphenyl]-3-yl)-3-(3-hydroxy-4-methoxyphenyl)prop-2-en-1-one |
| 32 | Tetrahydro curcumin | 1,7-bis(4-hydroxy-3-methoxyphenyl)heptane-3,5-dione |
| 33 | UBS109 | (3E,5E)-1-methyl-3,5-bis(pyridin-2-ylmethylene)piperidin-4-one |
| 34 | WZ26 | (1E,4E)-1-(3-bromo-4-hydroxyphenyl)-5-(4-hydroxy-3-methoxyphenyl)penta-1,4-dien-3-one |
| 35 | WZ35 | (1E,4E)-1-(4-hydroxy-3-methoxyphenyl)-5-(2-nitrophenyl)penta-1,4-dien-3-one |
| 36 | Alpha Curcumin | 1-methyl-4-(6-methylhept-5-en-2-yl)benzene |
| 37 | new-711 | (2E,5E)-2,5-bis((5-bromofuran-2-yl)methylene)cyclopentan-1-one |
| 38 | ECE Curcumin | ethyl (E)-7-(4-hydroxy-3-methoxyphenyl)-4-((E)-3-(4-hydroxy-3-methoxyphenyl)acryloyl)-5-oxohept-6-enoate |
| 39 | JC-9 | (1E,4Z,6E)-1,7-bis(3,4-dimethoxyphenyl)-5-hydroxyhepta-1,4,6-trien-3-one |
| 40 | LL80 | ethyl (2E,4Z,6E)-7-(3,4-dimethoxyphenyl)-4-((E)-3-(3,4-dimethoxyphenyl)acryloyl)-5-hydroxyhepta-2,4,6-trienoate |
| 41 | Xanthorrhizol | 2-methyl-5-[(2*R*)-6-methylhept-5-en-2-yl]phenol |
| 42 | 1 | 2-bromo-1-(4-methoxyphenyl)-3-(4-nitrophenyl)propane-1,3-dione |
| 43 | 2 | 3,5-bis((E)-3-hydroxybenzylidene)tetrahydro-4H-pyran-4-one |
| 44 | 3 | 3,5-bis((E)-3-fluorobenzylidene)piperidin-4-one acetate |
| 45 | 4 | (Z)-2-((1E,6E)-1,7-bis(4-hydroxy-3-methoxyphenyl)-5-oxohepta-1,6-dien-3-ylidene)hydrazine-1-carboxamide |
| 46 | 5 | (1E,6E)-1,7-bis(3,4-dihydroxyphenyl)hepta-1,6-diene-3,5-dione |
| 47 | 6 | methyl (2E,4Z,6E)-5-hydroxy-7-(4-hydroxy-3-methoxyphenyl)-4-((E)-3-(4-hydroxy-3-methoxyphenyl)acryloyl)hepta-2,4,6-trienoate |
| 48 | 8 | (3Z,5E)-4-hydroxy-6-(3-hydroxy-4-methoxyphenyl)hexa-3,5-dien-2-one |
| 49 | 9 | (3Z,5E)-4-hydroxy-6-(2,3,4-trimethoxyphenyl)hexa-3,5-dien-2-one |
| 50 | 10 | (1E,4Z,6E)-5-hydroxy-7-(4-hydroxy-3-methoxyphenyl)-1-(5-(hydroxymethyl)furan-2-yl)hepta-1,4,6-trien-3-one |
| 51 | 11 | (1E,4Z,6E)-5-hydroxy-7-(4-hydroxy-3-methoxyphenyl)-1-(thiophen-2-yl)hepta-1,4,6-trien-3-one |
| 52 | 12 | (1E,4Z,6E)-5-hydroxy-1,7-bis(2,4,5-trimethoxyphenyl)hepta-1,4,6-trien-3-one |
| 53 | 13 | (1E,4Z,6E)-5-hydroxy-1,7-bis(2,3,4-trimethoxyphenyl)hepta-1,4,6-trien-3-one |
| 54 | 14 | (1E,4Z,6E)-7-(3,4-dimethoxyphenyl)-5-hydroxy-1-(3-hydroxy-4-methoxyphenyl)hepta-1,4,6-trien-3-one |
| 55 | 15 | (1E,4Z,6E)-7-(3,4-dimethoxyphenyl)-5-hydroxy-1-(4-hydroxy-3-methoxyphenyl)hepta-1,4,6-trien-3-one |
| 56 | 16 | (Z)-2-((E)-3,4-dimethoxybenzylidene)-6-((E)-3-(3,4-dimethoxyphenyl)-1-hydroxyallylidene)cyclohexan-1-one |
| 57 | 17 | (1E,4E)-1,5-bis(4-(cyclopropylmethyl)-3,5-dimethoxyphenyl)penta-1,4-dien-3-one |
| 58 | 19 | 4,4'-((1E,1'E)-isoxazole-3,5-diylbis(ethene-2,1-diyl))bis(2-methoxyphenol) |
| 59 | acsjm5 | (1E,4Z,6E)-5-hydroxy-1,7-bis(5-methylfuran-2-yl)hepta-1,4,6-trien-3-one |
| 60 | 22 | (1E,6E)-1-(4-(((2R,3S,4R,5R,6R)-3,4,5,6-tetrahydroxytetrahydro-2H-pyran-2-yl)oxy)phenyl)-7-(4-(((2S,3R,4S,5S,6R)-3,4,5-trihydroxy-6-(hydroxymethyl)tetrahydro-2H-pyran-2-yl)oxy)phenyl)hepta-1,6-diene-3,5-dione |
| 61 | 23 | (1E,4E)-1,5-bis(4-hydroxyphenyl)penta-1,4-dien-3-one |
| 62 | 26 | N,N'-(((1E,6E)-3,5-dioxohepta-1,6-diene-1,7-diyl)bis(2-methoxy-4,1-phenylene))diacetamide |
| 63 | 27 | 4,4'-((1E,6E)-3,5-dioxohepta-1,6-diene-1,7-diyl)bis(2-methoxybenzamide) |
| 64 | 28 | (3E,5E)-3,5-bis(naphthalen-2-ylmethylene)piperidin-4-one |
| 65 | 29 | (1E,6E)-1,7-bis(3,4-dimethoxyphenyl)hepta-1,6-diene-3,5-dione |
| 66 | 30 | (1E,6E)-1,7-bis(3,4-dimethoxyphenyl)-4-methylhepta-1,6-diene-3,5-dione |
| 67 | 31 | ethyl (6E)-7-(3,4-dimethoxyphenyl)-4-((E)-3-(3,4-dimethoxyphenyl)acryloyl)-5-oxohepta-2,6-dienoate |
| 68 | 33 | (2E,7E)-5-(2-(3,4-dimethoxyphenyl)ethylidene)-1,9-bis(4-hydroxy-3-methoxyphenyl)nona-2,7-diene-4,6-dione |
| 69 | 34 | (2E,7E)-5-(2-(3-fluorophenyl)ethylidene)-1,9-bis(4-hydroxy-3-methoxyphenyl)nona-2,7-diene-4,6-dione |
| 70 | 35 | (2E,7E)-5-(2-(4-fluorophenyl)ethylidene)-1,9-bis(4-hydroxy-3-methoxyphenyl)nona-2,7-diene-4,6-dione |
| 71 | 36 | 4-((E)-7-(3,4-dimethoxyphenyl)-3-((E)-4-(3,4-dimethoxyphenyl)but-2-enoyl)-4-oxohepta-2,5-dien-1-yl)phthalaldehyde |
| 72 | 37 | (2E,7E)-1,9-bis(4-hydroxy-3-methoxyphenyl)-5-(2-(4-hydroxy-3-methoxyphenyl)ethylidene)nona-2,7-diene-4,6-dione |
| 73 | 40 | 1-(1,5-dimethyl-1H-benzo[d]imidazol-2-yl)-5-(4-hydroxy-3-methoxyphenyl)pentan-3-one |
| 74 | 41 | 1-(6-hydroxy-1-methyl-1H-benzo[d]imidazol-2-yl)-5-(3-hydroxy-4-methoxyphenyl)pentan-3-one |
| 75 | 42.1 | 3-(3,4-dimethoxy-5-methylbenzyl)-5-(3,4,5-trimethoxybenzyl)tetrahydro-4H-pyran-4-one |
| 76 | 42 | 3-(3,4-dimethoxy-5-methylbenzyl)-5-(3,4,5-trimethoxybenzyl)tetrahydro-4H-thiopyran-4-one |
| 77 | 43 | (1E,6E)-1,7-bis(2,3-dimethoxyphenyl)-4-(4-hydroxy-3-methoxybenzylidene)hepta-1,6-diene-3,5-dione |
| 78 | 44 | (1E,4E)-1-(2,6-dichlorophenyl)-5-(2,4-dimethoxy-6-((E)-4-methoxystyryl)phenyl)penta-1,4-dien-3-one |
| 79 | 45 | 3-(4-((1E,6E)-7-(3,4-dimethoxyphenyl)-3,5-dioxohepta-1,6-dien-1-yl)-2-methoxyphenoxy)-2-hydroxy-2-methyl-N-(4-nitro-3-(trifluoromethyl)phenyl)propanamide |
| 80 | 47 | (1E,6E)-1-(3-((dimethylamino)methyl)-4-hydroxyphenyl)-7-(4-hydroxy-3-methoxyphenyl)hepta-1,6-diene-3,5-dione |
| 81 | 50 | 2-((E)-2-chlorobenzylidene)-6-((E)-3,5-dimethoxybenzylidene)cyclohexan-1-one |
| 82 | 51 | 2-((E)-3,4-dihydroxybenzylidene)-6-((E)-3,5-dimethoxybenzylidene)cyclohexan-1-one |
| 83 | 52 | (1E,6E)-1,7-bis(4-hydroxy-3-(trifluoromethoxy)phenyl)hepta-1,6-diene-3,5-dione |
| 84 | 61 | 3-((4,6-bis((E)-3,4-dimethoxystyryl)pyrimidin-2-yl)amino)propan-1-ol |
| 85 | 62 | (1E,4E)-1,5-bis(1-methyl-1H-imidazol-2-yl)penta-1,4-dien-3-one |
| 86 | 64 | (3E,5E)-1-acryloyl-3,5-bis(pyridin-4-ylmethylene)piperidin-4-one |
| 87 | 67 | (2S,3S,4S,5R,6S)-3,4,5-trihydroxy-6-(4-((1E,6E)-7-(4-hydroxy-3-methoxyphenyl)-3,5-dioxohepta-1,6-dien-1-yl)-2-methoxyphenoxy)tetrahydro-2H-pyran-2-carboxylic acid |
| 88 | 68 | (2S,3S,4S,5R,6S)-6-(4-((1E,6E)-7-(4-(((2R,3R,4S,5S,6S)-6-carboxy-3,4,5-trihydroxytetrahydro-2H-pyran-2-yl)oxy)-3-methoxyphenyl)-3,5-dioxohepta-1,6-dien-1-yl)-2-methoxyphenoxy)-3,4,5-trihydroxytetrahydro-2H-pyran-2-carboxylic acid |
| 89 | 69 | ((1E,4E)-3-oxopenta-1,4-diene-1,5-diyl)bis(3,1-phenylene) bis(decanoate) |
| 90 | 70 | (1E,6E)-1-(4-hydroxy-3-methoxyphenyl)-7-(7-methyl-3-methylene-4-(prop-2-yn-1-yl)-3,4-dihydronaphthalen-2-yl)hepta-1,6-diene-3,5-dione |
| 91 | 71 | 3-((1E,4E)-5-(1-methyl-1H-imidazol-2-yl)-3-oxopenta-1,4-dien-1-yl)-4H-chromen-4-one |
| 92 | 73 | (E)-4-(4-chlorophenyl)-2-(5-ethyl-7-(4-methoxybenzylidene)-3-(4-methoxyphenyl)-3,3a,4,5,6,7-hexahydro-2H-pyrazolo[4,3-c]pyridin-2-yl)thiazole |
| 93 | 74 | (1E,4E)-1,5-bis(4-hydroxy-3-methoxyphenyl)penta-1,4-dien-3-one |
| 94 | 76 | (Z)-4-(3,5-di((E)-benzylidene)-4-oxocyclohexyl)-N-(2-fluorophenyl)-4-oxobut-2-enamide |
| 95 | 77 | (Z)-4-(3,5-di((E)-benzylidene)-4-oxocyclohexyl)-N-(2-methoxyphenyl)-4-oxobut-2-enamide |
| 96 | 78 | (Z)-4-(3,5-di((E)-benzylidene)-4-oxocyclohexyl)-N-(2,6-dichlorophenyl)-4-oxobut-2-enamide |
| 97 | 79 | 1-(2-(4-((2-methoxy-4-((1E,4E)-3-oxo-5-(3,4,5-trimethoxyphenyl)penta-1,4-dien-1-yl)phenoxy)methyl)-1H-1,2,3-triazol-1-yl)ethyl)indoline-2,3-dione |
| 98 | 80 | 1-(2-(4-((4-((1E,4E)-5-(3,4-dimethoxyphenyl)-3-oxopenta-1,4-dien-1-yl)-2-methoxyphenoxy)methyl)-1H-1,2,3-triazol-1-yl)ethyl)indoline-2,3-dione |
| 99 | 81 | 1-(2-(4-((2-methoxy-4-((1E,4E)-5-(4-methoxyphenyl)-3-oxopenta-1,4-dien-1-yl)phenoxy)methyl)-1H-1,2,3-triazol-1-yl)ethyl)indoline-2,3-dione |
| 100 | 82 | 1-(2-(4-((2-methoxy-4-((1E,4E)-5-(naphthalen-1-yl)-3-oxopenta-1,4-dien-1-yl)phenoxy)methyl)-1H-1,2,3-triazol-1-yl)ethyl)indoline-2,3-dione |
| 101 | 6R7R bisbolone | (R)-3-Methyl-6-((R)-6-methylhept-5-en-2-yl)cyclohex-2-enone |
| 102 | Alpha Cadinol | (1*R*,4*S*,4*aR*,8*aR*)-1,6-dimethyl-4-propan-2-yl-3,4,4*a*,7,8,8*a*-hexahydro-2*H*-naphthalen-1-ol |
| 103 | Alpha Pinene | 2,6,6-trimethylbicyclo[3.1.1]hept-2-ene |
| 104 | Alpha Zingiberene | (5*S*)-2-methyl-5-[(2*R*)-6-methylhept-5-en-2-yl]cyclohexa-1,3-diene |
| 105 | ar-Turmerol | 2-methyl-6-(4-methylphenyl)hept-2-en-4-ol |
| 106 | Beta Caryophyllene | (1*R*,4*E*,9*S*)-4,11,11-trimethyl-8-methylidenebicyclo[7.2.0]undec-4-ene |
| 107 | Beta Sesquiphellandrene | (3*R*)-3-[(2*S*)-6-methylhept-5-en-2-yl]-6-methylidenecyclohexene |
| 108 | Beta Turmeron | 2-methyl-6-(4-methylidenecyclohex-2-en-1-yl)hept-2-en-4-one |
| 109 | E Alpha Atlantone | (5*E*)-2-methyl-6-(4-methylcyclohex-3-en-1-yl)hepta-2,5-dien-4-one |
| 110 | Gamma Curcumene | 1-methyl-4-[(2*R*)-6-methylhept-5-en-2-yl]cyclohexa-1,3-diene |
| 111 | P- Cymene | 1-methyl-4-propan-2-ylbenzene |
| 112 | Vinyl Propionate | ethenyl propanoate |
| 113 | 1 | N-(2-chlorophenyl)-3,5-bis((E)-4-hydroxy-3-methylstyryl)-1H-pyrazole-1-carboxamide |
| 114 | 2 | N-(4-chlorophenyl)-3,5-bis((E)-4-hydroxy-3-methylstyryl)-1H-pyrazole-1-carboxamide |
| 115 | 3 | N-(4-fluorophenyl)-3,5-bis((E)-4-hydroxy-3-methylstyryl)-1H-pyrazole-1-carboxamide |
| 116 | 4 | N-(4-bromophenyl)-3,5-bis((E)-4-hydroxy-3-methylstyryl)-1H-pyrazole-1-carboxamide |
| 117 | 5 | N-(3-chloro-4-fluorophenyl)-3,5-bis((E)-4-hydroxy-3-methylstyryl)-1H-pyrazole-1-carboxamide |
| 118 | 6 | 3,5-bis((E)-4-hydroxy-3-methylstyryl)-N-(p-tolyl)-1H-pyrazole-1-carboxamide |
| 119 | 7 | 3,5-bis((E)-4-hydroxy-3-methylstyryl)-N-(o-tolyl)-1H-pyrazole-1-carboxamide |
| 120 | 8 | N-(2,4-dimethylphenyl)-3,5-bis((E)-4-hydroxy-3-methylstyryl)-1H-pyrazole-1-carboxamide |
| 121 | 9 | N-(2,6-dimethylphenyl)-3,5-bis((E)-4-hydroxy-3-methylstyryl)-1H-pyrazole-1-carboxamide |
| 122 | 10 | (3,5-bis((E)-4-hydroxy-3-methoxystyryl)-1H-pyrazol-1-yl)(phenyl)methanone |
| 123 | 11 | (3,5-bis((E)-4-hydroxy-3-methoxystyryl)-1H-pyrazol-1-yl)(2-bromophenyl)methanone |
| 124 | 12 | 4,6-bis((E)-4-hydroxy-3-methoxystyryl)pyrimidin-2(1H)-one |
| 125 | 13 | 4,4'-((1E,1'E)-(2-imino-1,2-dihydropyrimidine-4,6-diyl)bis(ethene-2,1-diyl))bis(2-methoxyphenol) |
| 126 | 14 | 4,6-bis((E)-4-hydroxy-3-methoxystyryl)pyrimidine-2(1H)-thione |
| 127 | 1 | (1E,4Z,6E)-5-hydroxy-1,7-bis(4-hydroxy-3-methoxyphenyl)hepta-1,4,6-trien-3-one |
| 128 | 2 | (1E,4Z,6E)-5-hydroxy-1,7-bis(4-methoxyphenyl)hepta-1,4,6-trien-3-one |
| 129 | 4 | (1E,4Z,6E)-5-hydroxy-1,7-bis(3,4,5-trimethoxyphenyl)hepta-1,4,6-trien-3-one |
| 130 | acsjm6 | (1E,4Z,6E)-1,7-di(furan-2-yl)-5-hydroxyhepta-1,4,6-trien-3-one |
| 131 | 7 | (1E,4E)-1,5-bis(3,4,5-trimethoxyphenyl)penta-1,4-dien-3-one |
| 132 | 8 | (E)-3-((E)-3,4,5-trimethoxybenzylidene)-6-(3,4,5-trimethoxyphenyl)hex-5-ene-2,4-dione |
| 133 | 9 | (1E,4E)-1-(3,4-dimethoxyphenyl)-5-(3,4,5-trimethoxyphenyl)penta-1,4-dien-3-one |
| 134 | 10 | (1E,4E)-1-(3-methylthiophen-2-yl)-5-(3,4,5-trimethoxyphenyl)penta-1,4-dien-3-one |
| 135 | 13 | (1E,6E)-4-(4-hydroxy-3-methoxybenzylidene)-1,7-bis(4-methoxyphenyl)hepta-1,6-diene-3,5-dione |
| 136 | 14 | (1E,6E)-4-(3,4-dimethoxybenzylidene)-1,7-bis(4-methoxyphenyl)hepta-1,6-diene-3,5-dione |
| 137 | 15 | (1E,6E)-1,7-bis(4-methoxyphenyl)-4-(3,4,5-trimethoxybenzylidene)hepta-1,6-diene-3,5-dione |
| 138 | 16 | (1E,6E)-1,7-bis(3,4-dimethoxyphenyl)-4-(3,4,5-trimethoxybenzylidene)hepta-1,6-diene-3,5-dione |
| 139 | 17 | (1E,6E)-1,7-bis(3,4-dimethoxyphenyl)-4-(4-hydroxy-3-methoxybenzylidene)hepta-1,6-diene-3,5-dione |
| 140 | 18 | (1E,6E)-4-(3,4-dimethoxybenzylidene)-1,7-bis(3,4-dimethoxyphenyl)hepta-1,6-diene-3,5-dione |
| 141 | 19 | (1E,6E)-1,7-bis(3,4-dimethoxyphenyl)-4-(4-(vinylideneamino)benzylidene)hepta-1,6-diene-3,5-dione compound with dihydrogen (1:2) |
| 142 | 20 | (1E,6E)-1,7-bis(3,4-dimethoxyphenyl)-4-(3-fluorobenzylidene)hepta-1,6-diene-3,5-dione |
| 143 | 21 | (1E,6E)-1,7-bis(3,4-dimethoxyphenyl)-4-(4-fluorobenzylidene)hepta-1,6-diene-3,5-dione |
| 144 | 22 | (1E,6E)-1,7-bis(3,4-dimethoxyphenyl)-4-(4-hydroxybenzylidene)hepta-1,6-diene-3,5-dione |
| 145 | 23 | (1E,6E)-4-(2,5-dimethoxybenzylidene)-1,7-bis(4-hydroxy-3-methoxyphenyl)hepta-1,6-diene-3,5-dione |
| 146 | 24 | (1E,6E)-1,7-bis(3,4-dimethoxyphenyl)-4-(4-ethylbenzylidene)hepta-1,6-diene-3,5-dione |
| 147 | 25 | (1E,6E)-4-(2,3-dimethoxybenzylidene)-1,7-bis(3,4-dimethoxyphenyl)hepta-1,6-diene-3,5-dione |
| 148 | 26 | (1E,6E)-4-(3,4-dimethoxybenzylidene)-1,7-bis(3,4,5-trimethoxyphenyl)hepta-1,6-diene-3,5-dione |
| 149 | 27 | (1E,6E)-4-(2,5-dimethoxybenzylidene)-1,7-bis(3,4-dimethoxyphenyl)hepta-1,6-diene-3,5-dione |
| 150 | 28 | (1E,6E)-4-(2,4-dimethoxybenzylidene)-1,7-bis(3,4-dimethoxyphenyl)hepta-1,6-diene-3,5-dione |
| 151 | 29 | (1E,6E)-4-(4-hydroxy-3-methoxybenzylidene)-1,7-bis(4-hydroxy-3-methoxyphenyl)hepta-1,6-diene-3,5-dione |
| 152 | 30 | (1E,6E)-4-(3,4-dimethoxybenzylidene)-1,7-bis(4-hydroxy-3-methoxyphenyl)hepta-1,6-diene-3,5-dione |
| 153 | 31 | (1E,6E)-4-(2,4-dimethoxybenzylidene)-1,7-bis(4-hydroxy-3-methoxyphenyl)hepta-1,6-diene-3,5-dione |
| 154 | 32 | (1E,6E)-4-(3-fluorobenzylidene)-1,7-bis(4-hydroxy-3-methoxyphenyl)hepta-1,6-diene-3,5-dione |
| 155 | 33 | (1E,6E)-4-(4-fluorobenzylidene)-1,7-bis(4-hydroxy-3-methoxyphenyl)hepta-1,6-diene-3,5-dione |
| 156 | 34 | (1E,6E)-4-(4-ethylbenzylidene)-1,7-bis(4-hydroxy-3-methoxyphenyl)hepta-1,6-diene-3,5-dione |
| 157 | 35 | (1E,6E)-4-(2,3-dimethoxybenzylidene)-1,7-bis(4-hydroxy-3-methoxyphenyl)hepta-1,6-diene-3,5-dione |
| 158 | 36 | (1E,6E)-1,7-bis(4-hydroxy-3-methoxyphenyl)-4-(3-methoxybenzylidene)hepta-1,6-diene-3,5-dione |
| 159 | 37 | (1E,6E)-1,7-bis(3,4-dimethoxyphenyl)-4-((5-methylfuran-2-yl)methylene)hepta-1,6-diene-3,5-dione |
| 160 | 38 | ((1E,6E)-4-(hydroxymethylene)-3,5-dioxohepta-1,6-diene-1,7-diyl)bis(2-methoxy-4,1-phenylene) diacetate |
| 161 | 39 | (1E,6E)-4-(hydroxymethylene)-1,7-bis(4-methoxyphenyl)hepta-1,6-diene-3,5-dione |
| 162 | 40 | (1E,6E)-1,7-bis(3,4-dimethoxyphenyl)-4-(hydroxymethylene)hepta-1,6-diene-3,5-dione |
| 163 | Alpha-turmeron | (S)-2-methyl-6-((R)-4-methylcyclohexa-2,4-dien-1-yl)hept-2-en-4-one |
| 164 | new-715 | (2E,5E)-2,5-bis(thiophen-2-ylmethylene)cyclopentan-1-one |
| 165 | beta-tur | (S)-2-methyl-6-((S)-4-methylenecyclohex-2-en-1-yl)hept-2-en-4-one |
| 166 | BDC | (1E,6E)-1,7-bis(4-hydroxyphenyl)hepta-1,6-diene-3,5-dione |
| 167 | DMC | (1E,6E)-1-(4-hydroxy-3-methoxyphenyl)-7-(4-hydroxyphenyl)hepta-1,6-diene-3,5-dione |
| 168 | 716 | (1E,4E)-1,5-di(thiophen-2-yl)penta-1,4-dien-3-one |
| 169 | bisacurone | (S)-6-((1R,4S,5S)-4,5-dihydroxy-4-methylcyclohex-2-en-1-yl)-2-methylhept-2-en-4-one |
| 170 | caleibin | (E)-4-(4-hydroxy-3-methoxyphenyl)-2-oxobut-3-en-1-yl (E)-3-(4-hydroxy-3-methoxyphenyl)acrylate |
| 171 | curcumol | (3S,3aS,5S,8aS)-5-isopropyl-3-methyl-8-methyleneoctahydro-6H-3a,6-epoxyazulen-6-ol |
| 172 | cyclocurcumin | (E)-2-(4-hydroxy-3-methoxyphenyl)-6-(4-hydroxy-3-methoxystyryl)-2,3-dihydro-4H-pyran-4-one |
| 173 | furanodiene | (5E,9E)-3,6,10-trimethyl-4,7,8,11-tetrahydrocyclodeca[b]furan |
| 174 | 717 | (2E,6E)-2,6-bis(thiophen-2-ylmethylene)cyclohexan-1-one |
| 175 | germachone | (3E,7E)-3,7-dimethyl-10-(propan-2-ylidene)cyclodeca-3,7-dien-1-one |
| 176 | 1a | toluene |
| 177 | 1b | 2-methylnaphthalene |
| 178 | 1c | 2-methylthiophene |
| 179 | 1d | 3-methyl-1H-indole |
| 180 | 1e | 5-methyl-1H-indole |
| 181 | 1f | 1-bromo-4-((p-tolyloxy)methyl)benzene |
| 182 | 1g | 4-methyl-1H-imidazole |
| 183 | 1h | 2,3-dimethylthiophene |
| 184 | 1i | 2,5-dimethylthiophene |
| 185 | CA1 | 3,3'-((1E,3Z,6E)-3-hydroxy-5-oxohepta-1,3,6-triene-1,7-diyl)bis(4-ethyl-7-fluoro-2H-chromen-2-one) |
| 186 | CA2 | 3,3'-((1E,3Z,6E)-3-hydroxy-5-oxohepta-1,3,6-triene-1,7-diyl)bis(7-chloro-4-ethyl-2H-chromen-2-one) |
| 187 | CA3 | 3,3'-((1E,3Z,6E)-3-hydroxy-5-oxohepta-1,3,6-triene-1,7-diyl)bis(7-bromo-4-ethyl-2H-chromen-2-one) |
| 188 | CA4 | 3,3'-((1E,3Z,6E)-3-hydroxy-5-oxohepta-1,3,6-triene-1,7-diyl)bis(4-ethyl-7-iodo-2H-chromen-2-one) |
| 189 | CA5 | 3,3'-((1E,3Z,6E)-3-hydroxy-5-oxohepta-1,3,6-triene-1,7-diyl)bis(4-ethyl-6-fluoro-2H-chromen-2-one) |
| 190 | CA6 | 3,3'-((1E,3Z,6E)-3-hydroxy-5-oxohepta-1,3,6-triene-1,7-diyl)bis(6-chloro-4-ethyl-2H-chromen-2-one) |
| 191 | CA7 | 3,3'-((1E,3Z,6E)-3-hydroxy-5-oxohepta-1,3,6-triene-1,7-diyl)bis(6-bromo-4-ethylchroman-2-one) |
| 192 | CA8 | 3,3'-((1E,3Z,6E)-3-hydroxy-5-oxohepta-1,3,6-triene-1,7-diyl)bis(4-ethyl-6-iodo-2H-chromen-2-one) |
| 193 | 19 | 4,4'-((1E,1'E)-(1H-pyrazole-3,5-diyl)bis(ethene-2,1-diyl))bis(2-methoxyphenol) |
| 194 | 26 | ((1E,6E)-3,5-dioxohepta-1,6-diene-1,7-diyl)bis(2-methoxy-4,1-phenylene) bis(2-aminoacetate) |
| 195 | 27 | ((1E,6E)-3,5-dioxohepta-1,6-diene-1,7-diyl)bis(2-methoxy-4,1-phenylene) bis(5-(benzo[d][1,3]dioxol-5-yl)penta-2,4-dienoate) |
| 196 | 35 | (1E,6E)-1,7-bis(4-hydroxy-3-methoxyphenyl)hepta-1,6-diene-3,5-dione O,O-dibenzyl dioxime |
| 197 | 118 | dimethyl 2,2'-((((1E,3Z,6E)-3-hydroxy-5-oxohepta-1,3,6-triene-1,7-diyl)bis(2-methoxy-4,1-phenylene))bis(oxy))diacetate |
| 198 | 120 | ethyl (4Z,6E)-5-hydroxy-7-(4-hydroxy-3-methoxyphenyl)-4-((E)-3-(4-hydroxy-3-methoxyphenyl)acryloyl)hepta-4,6-dienoate |
| 199 | 121 | (4Z,6E)-5-hydroxy-7-(4-hydroxy-3-methoxyphenyl)-4-((E)-3-(4-hydroxy-3-methoxyphenyl)acryloyl)hepta-4,6-dienoic acid |
| 200 | 137 | 1,3-bis(3,4-dimethoxyphenyl)propane-1,3-dione |
| 201 | 188 | (1E,4Z,6E)-5-hydroxy-1,7-bis(4-hydroxy-3-nitrophenyl)hepta-1,4,6-trien-3-one |
| 202 | 191 | (1E,4Z,6E)-5-hydroxy-1,7-bis(4-nitrophenyl)hepta-1,4,6-trien-3-one |
| 203 | 193 | (1E,4Z,6E)-5-hydroxy-1,7-bis(4-hydroxy-3-methoxy-5-nitrophenyl)hepta-1,4,6-trien-3-one |
| 204 | 206 | (E)-2,6-dibenzyl-1-(4-(3-oxo-3-phenylprop-1-en-1-yl)benzoyl)piperidin-4-one |
| 205 | 212 | (E)-2,6-dibenzyl-1-(4-(3-(3,4-dimethoxyphenyl)-3-oxoprop-1-en-1-yl)benzoyl)piperidin-4-one |
| 206 | 213 | (E)-2,6-dibenzyl-1-(4-(3-(4-nitrophenyl)-3-oxoprop-1-en-1-yl)benzoyl)piperidin-4-one |
| 207 | 214 | (E)-2,6-dibenzyl-1-(4-(3-(4-hydroxyphenyl)-3-oxoprop-1-en-1-yl)benzoyl)piperidin-4-one |
| 208 | 220 | (E)-2,6-dibenzyl-1-(3-(3,4-dimethoxyphenyl)acryloyl)piperidin-4-one |
| 209 | 242 | (E)-1-phenyl-3-(pyridin-2-yl)prop-2-en-1-one |
| 210 | 244 | 2,6-di((E)-benzylidene)cyclohexan-1-one |
| 211 | 249 | ethyl (4Z,6E)-5-hydroxy-7-(3-methoxyphenyl)-4-((E)-3-(3-methoxyphenyl)acryloyl)hepta-4,6-dienoate |
| 212 | 251 | (1E,4Z,6E)-1,7-bis(2,4-difluorophenyl)-5-hydroxyhepta-1,4,6-trien-3-one |
| 213 | 253 | (1E,4Z,6E)-1,7-bis(2-fluoro-6-methoxyphenyl)-5-hydroxyhepta-1,4,6-trien-3-one |
| 214 | 256 | (1E,4Z,6E)-1,7-bis(3,4-dimethoxy-5-nitrophenyl)-5-hydroxyhepta-1,4,6-trien-3-one |
| 215 | 261 | ethyl (4Z,6E)-5-hydroxy-7-(3,4,5-trimethoxyphenyl)-4-((E)-3-(3,4,5-trimethoxyphenyl)acryloyl)hepta-4,6-dienoate |
| 216 | 262 | ethyl (4Z,6E)-7-(3-(dimethylamino)-4-methoxyphenyl)-4-((E)-3-(3-(dimethylamino)-4-methoxyphenyl)acryloyl)-5-hydroxyhepta-4,6-dienoate |
| 217 | 263 | (1E,4Z,6E)-5-hydroxy-1,7-bis(3-methoxy-4-propoxyphenyl)-4-propylhepta-1,4,6-trien-3-one |
| 218 | 266 | (1E,6E)-5-hydroxy-1,7-bis(3-methoxy-4-propoxyphenyl)-4,4-dipropylhepta-1,6-dien-3-one |
| 219 | 290 | 3,5-bis((E)-2-fluorobenzylidene)piperidin-4-one acetate |
| 220 | 297 | (1E,6E)-1,7-bis(3-fluoro-4-hydroxyphenyl)hepta-1,6-diene-3,5-dione |
| 221 | 302 | 2,6-bis((E)-3-fluoro-4-hydroxybenzylidene)cyclohexan-1-one |
| 222 | 309 | 3,5-bis((E)-3-ethoxy-4-hydroxybenzylidene)-1-methylpiperidin-4-one |
| 223 | 351 | (3E,5E)-1-ethyl-3,5-bis((E)-3-(4-hydroxy-3-methoxy-5-methylphenyl)allylidene)piperidin-4-one |
| 224 | 478 | (1E,4E)-1,5-bis(3,4-dimethoxyphenyl)penta-1,4-dien-3-one |
| 225 | DAC | ((1E,3Z,6E)-3-hydroxy-5-oxohepta-1,3,6-triene-1,7-diyl)bis(2-methoxy-4,1-phenylene) diacetate |
| 226 | iiib | ((1E,1'E)-(4-oxopiperidine-3,5-diylidene)bis(methaneylylidene))bis(2-ethoxy-4,1-phenylene) dibutyrate |
| 227 | iva | ((1Z,1'Z)-(1-acetylpiperidine-3,5-diylidene)bis(methaneylylidene))bis(2-ethoxy-4,1-phenylene) dipropionate |
| 228 | ivb | ((1Z,1'Z)-(1-benzoyl-4-oxopiperidine-3,5-diylidene)bis(methaneylylidene))bis(2-ethoxy-4,1-phenylene) dibutyrate |
| 229 | viia | 1-acetyl-3,5-bis((E)-3-ethoxy-4-propoxybenzylidene)piperidin-4-one |
| 230 | viib | 1-acetyl-3,5-bis((E)-3-methoxy-4-propoxybenzylidene)piperidin-4-one |
| 231 | viic | 3,5-bis((E)-3-methoxy-4-propoxybenzylidene)-4-oxo-N-phenylpiperidine-1-carbothioamide |
| 232 | 5 | (1E,6E)-1,7-bis(4-(methylthio)phenyl)hepta-1,6-diene-3,5-dione |
| 233 | 7 | (1E,6E)-1,7-bis(4-(methylsulfonyl)phenyl)hepta-1,6-diene-3,5-dione |
| 234 | 8 | (E)-N-(3-(3-(3-bromo-2,4,6-trimethoxyphenyl)acryloyl)phenyl)-2-fluorobenzamide |
| 235 | 10 | (E)-N-(3-(3-(3-bromo-2,4,6-trimethoxyphenyl)acryloyl)phenyl)thiophene-2-carboxamide |
| 236 | A2 | 2,6-bis((E)-3,4-dihydroxybenzylidene)cyclohexan-1-one |
| 237 | A13 | 2,5-bis((E)-4-(3-(dimethylamino)propoxy)benzylidene)cyclopentan-1-one |
| 238 | B82 | (1E,4E)-1,5-bis(5-bromo-2-ethoxyphenyl)penta-1,4-dien-3-one |
| 239 | BAT3 | (3E,5E)-3,5-bis((1H-indol-3-yl)methylene)piperidin-4-one |
| 240 | PAC | 3,5-bis((E)-4-hydroxy-3-methoxybenzylidene)-1-methylpiperidin-4-one |
| 241 | RA1 | 1,5-bis(2-(trifluoromethyl)phenyl)pentan-3-one |
| 242 | RA2 | 1,5-bis(3-(trifluoromethyl)phenyl)pentan-3-one |
| 243 | RA3 | 1,5-bis(4-(trifluoromethyl)phenyl)pentan-3-one |
| 244 | RA4 | 1-phenyl-5-(2-(trifluoromethyl)phenyl)pentan-3-one |
| 245 | RA5 | 1-phenyl-5-(3-(trifluoromethyl)phenyl)pentan-3-one |
| 246 | RA6 | 1-phenyl-5-(4-(trifluoromethyl)phenyl)pentan-3-one |
| 247 | RB1 | 1,5-bis(2-hydroxyphenyl)pentan-3-one |
| 248 | RB2 | 1,5-bis(3-hydroxyphenyl)pentan-3-one |
| 249 | RB3 | 1,5-bis(4-hydroxyphenyl)pentan-3-one |
| 250 | RB4 | 1-(2-hydroxyphenyl)-5-phenylpentan-3-one |
| 251 | RB5 | 1-(3-hydroxyphenyl)-5-phenylpentan-3-one |
| 252 | RB6 | 1-(4-hydroxyphenyl)-5-phenylpentan-3-one |
| 253 | RC1 | 1,5-bis(2-methoxyphenyl)pentan-3-one |
| 254 | RC2 | 1,5-bis(3-methoxyphenyl)pentan-3-one |
| 255 | RC3 | 1,5-bis(4-methoxyphenyl)pentan-3-one |
| 256 | RC4 | 1-(2-methoxyphenyl)-5-phenylpentan-3-one |
| 257 | RC5 | 1-(3-methoxyphenyl)-5-phenylpentan-3-one |
| 258 | RC6 | 1-(4-methoxyphenyl)-5-phenylpentan-3-one |
| 259 | STO3 | 1,2-bis(3,5-bis((E)-2-chlorobenzylidene)-4-oxopiperidin-1-yl)ethane-1,2-dione |
| 260 | STO8 | 1,2-bis(3,5-bis((E)-4-nitrobenzylidene)-4-oxopiperidin-1-yl)ethane-1,2-dione |
| 261 | 1 | tert-butyl 3-acetyl-4-oxopentanoate |
| 262 | 2 | 3-acetyl-4-oxopentanoic acid |
| 263 | 3 | tert-butyl (E)-3-cinnamoyl-4-oxo-6-phenylhex-5-enoate |
| 264 | 4 | tert-butyl (E)-6-(4-hydroxy-3-methoxyphenyl)-3-((E)-3-(4-hydroxy-3-methoxyphenyl)acryloyl)-4-oxohex-5-enoate |
| 265 | 5 | tert-butyl (E)-6-(3-methoxyphenyl)-3-((E)-3-(3-methoxyphenyl)acryloyl)-4-oxohex-5-enoate |
| 266 | 6 | ((1E,6E)-4-(2-(tert-butoxy)-2-oxoethyl)-3,5-dioxohepta-1,6-diene-1,7-diyl)bis(2-methoxy-4,1-phenylene) diacetate |
| 267 | 7 | (E)-3-cinnamoyl-4-oxo-6-phenylhex-5-enoic acid |
| 268 | 8 | (E)-6-(4-hydroxy-3-methoxyphenyl)-3-((E)-3-(4-hydroxy-3-methoxyphenyl)acryloyl)-4-oxohex-5-enoic acid |
| 269 | 9 | (E)-6-(3-methoxyphenyl)-3-((E)-3-(3-methoxyphenyl)acryloyl)-4-oxohex-5-enoic acid |
| 270 | 10 | (E)-6-(4-acetoxy-3-methoxyphenyl)-3-((E)-3-(4-acetoxy-3-methoxyphenyl)acryloyl)-4-oxohex-5-enoic acid |
| 271 | 9 | (Z)-5-hydroxy-1,7-bis(4-hydroxy-3-methoxyphenyl)hept-4-en-3-one |
| 272 | 10 | 5-hydroxy-1,7-bis(4-hydroxy-3-methoxyphenyl)heptan-3-one |
| 273 | 12 | (1E,6E)-1,7-bis(3-ethoxy-4-hydroxyphenyl)hepta-1,6-diene-3,5-dione |
| 274 | 14 | ((1E,6E)-3,5-dioxohepta-1,6-diene-1,7-diyl)bis(2-methoxy-4,1-phenylene) bis(2-chloroacetate) |
| 275 | 16 | (1E,6E)-1,7-bis(2-hydroxyphenyl)hepta-1,6-diene-3,5-dione |
| 276 | 20 | ((1E,6E)-3,5-dioxohepta-1,6-diene-1,7-diyl)bis(2-methoxy-4,1-phenylene) diacetate |
| 277 | 21 | (1E,6E)-1-(3-methoxy-4-(((2S,3R,4S,5S,6R)-3,4,5-trihydroxy-6-(hydroxymethyl)tetrahydro-2H-pyran-2-yl)oxy)phenyl)-7-(3-methoxy-4-(((3S,4R,5R,6S)-3,4,5-trihydroxy-6-(hydroxymethyl)tetrahydro-2H-pyran-2-yl)oxy)phenyl)hepta-1,6-diene-3,5-dione |
| 278 | 22 | ((1E,6E)-3,5-dioxohepta-1,6-diene-1,7-diyl)bis(2-methoxy-4,1-phenylene) disulfuramidite |
| 279 | 25 | 4-((1E,4E)-5-(3,5-dimethoxy-4-(sulfamoyloxy)phenyl)-3-oxopenta-1,4-dien-1-yl)-2,6-dimethoxyphenyl sulfamate |
| 280 | 26 | (1E,4E)-1,5-bis(2,4,6-trimethoxyphenyl)penta-1,4-dien-3-one |
| 281 | 27 | (1E,6E)-1,7-bis(2-hydroxyphenyl)-4,4-dimethylhepta-1,6-diene-3,5-dione |
| 282 | 28 | (1E,6E)-1,7-bis(2,3,4-trimethoxyphenyl)hepta-1,6-diene-3,5-dione |
| 283 | 29 | dimethyl 4,4'-((1E,6E)-3,5-dioxohepta-1,6-diene-1,7-diyl)dibenzoate |
| 284 | 30 | (1E,6E)-1,7-bis(4-methoxydibenzo[b,d]furan-2-yl)hepta-1,6-diene-3,5-dione |
| 285 | 32 | (5Z,5'Z)-5,5'-((2E,4Z,7E)-4-hydroxy-6-oxonona-2,4,7-triene-1,9-diylidene)bis(3,4-dimethoxyfuran-2(5H)-one) |
| 286 | 33 | (1E,4Z,6E)-5-hydroxy-1,7-di(naphthalen-2-yl)hepta-1,4,6-trien-3-one |
| 287 | 34 | (1E,4Z,6E)-5-hydroxy-1,7-bis(5-methylthiophen-2-yl)hepta-1,4,6-trien-3-one |
| 288 | 35 | (1E,4Z,6E)-5-hydroxy-1,7-bis(3-methylfuran-2-yl)hepta-1,4,6-trien-3-one |
| 289 | 43 | ((1E,6E)-3,5-dioxohepta-1,6-diene-1,7-diyl)bis(2-methoxy-4,1-phenylene) (2E,2'E,4E,4'E)-bis(5-(benzo[d][1,3]dioxol-5-yl)penta-2,4-dienoate) |
| 290 | 44 | (1E,4Z,6E)-5-hydroxy-1-(4-hydroxy-3-methoxyphenyl)-7-(5-methylfuran-2-yl)hepta-1,4,6-trien-3-one |
| 291 | 45 | (1E,4Z,6E)-5-hydroxy-7-(5-(hydroxymethyl)furan-2-yl)-1-(3,4,5-trimethoxyphenyl)hepta-1,4,6-trien-3-one |
| 292 | 46 | (1E,4Z,6E)-5-hydroxy-7-(5-(hydroxymethyl)furan-2-yl)-1-(4-methoxyphenyl)hepta-1,4,6-trien-3-one |
| 293 | 50 | 4-((1E,3Z,6E)-3-hydroxy-7-(3-methoxy-4-((4-methoxy-4-oxobutanoyl)oxy)phenyl)-5-oxohepta-1,3,6-trien-1-yl)phenyl methyl succinate |
| 294 | 51 | O,O'-(((1E,3Z,6E)-3-hydroxy-5-oxohepta-1,3,6-triene-1,7-diyl)bis(2-methoxy-4,1-phenylene)) dimethyl disuccinate |
| 295 | 52 | diethyl O,O'-(((1E,3Z,6E)-3-hydroxy-5-oxohepta-1,3,6-triene-1,7-diyl)bis(2-methoxy-4,1-phenylene)) disuccinate |
| 296 | 53 | O,O'-(((1E,3Z,6E)-3-hydroxy-5-oxohepta-1,3,6-triene-1,7-diyl)bis(4,1-phenylene)) dimethyl disuccinate |
| 297 | 59 | (1E,4Z,6E)-5-hydroxy-7-(4-hydroxy-3-methoxyphenyl)-1-(4-hydroxyphenyl)hepta-1,4,6-trien-3-one |
| 298 | 60 | (1E,4Z,6E)-5-hydroxy-1,7-bis(4-hydroxyphenyl)hepta-1,4,6-trien-3-one |
| 299 | 62 | 4-((E)-2-(5-((E)-4-butoxy-3-methoxystyryl)-1H-pyrazol-3-yl)vinyl)-2-methoxyphenol |
| 300 | 64 | 3,5-bis((E)-4-butoxy-3-methoxystyryl)isoxazole |
| 301 | 65 | 3,5-bis((E)-3-methoxy-4-(octyloxy)styryl)isoxazole |
| 302 | 70 | 4-(3,5-bis((E)-4-hydroxy-3-methoxystyryl)-1H-pyrazol-1-yl)benzoic acid |
| 303 | 72 | (1E,4Z,6E)-1,7-bis(4-hydroxy-3-methoxyphenyl)-5-(isopropylamino)hepta-1,4,6-trien-3-one |
| 304 | 73 | (1E,3Z,5Z,6E)-1,7-bis(4-hydroxy-3-methoxyphenyl)hepta-1,6-diene-3,5-dione O,O-diphenyl dioxime |
| 305 | 78 | sodium 4-(3,5-bis((E)-4-hydroxy-3-methoxystyryl)-1H-pyrazol-1-yl)benzoate |
| 306 | 79 | 1-(4-hydroxy-3-methoxyphenyl)-7-phenylheptan-3-one |
| 307 | 80 | (E)-1-(4-hydroxy-3-methoxyphenyl)-7-phenylhept-1-en-3-one |
| 308 | 83.1 | ethyl (E)-4-fluoro-7-(4-hydroxy-3-methoxyphenyl)-4-((E)-3-(4-hydroxy-3-methoxyphenyl)acryloyl)-5-oxohept-6-enoate |
| 309 | 84 | ethyl (2E,4Z,6E)-5-hydroxy-7-(4-hydroxy-3-methoxyphenyl)-4-((E)-3-(4-hydroxy-3-methoxyphenyl)acryloyl)hepta-2,4,6-trienoate |
| 310 | 85 | ethyl (2E,4Z,6E)-5-hydroxy-7-(3-methoxy-4-((tetrahydro-2H-pyran-2-yl)oxy)phenyl)-4-((E)-3-(3-methoxy-4-((tetrahydro-2H-pyran-2-yl)oxy)phenyl)acryloyl)hepta-2,4,6-trienoate |
| 311 | 89 | ethyl (4Z,6E)-7-(3,4-dimethoxyphenyl)-5-hydroxy-4-((E)-3-(4-hydroxy-3-methoxyphenyl)acryloyl)hepta-4,6-dienoate |
| 312 | 90 | ethyl (4Z,6E)-7-(3,4-dimethoxyphenyl)-4-((E)-3-(3,4-dimethoxyphenyl)acryloyl)-5-hydroxyhepta-2,4,6-trienoate |
| 313 | 91 | methyl (4Z,6E)-7-(3,4-dimethoxyphenyl)-4-((E)-3-(3,4-dimethoxyphenyl)acryloyl)-5-hydroxyhepta-2,4,6-trienoate |
| 314 | 92 | (4Z,6E)-7-(3,4-dimethoxyphenyl)-4-((E)-3-(3,4-dimethoxyphenyl)acryloyl)-5-hydroxyhepta-2,4,6-trienenitrile |
| 315 | 95 | (1E,6E)-1,7-bis(3,4-dimethoxyphenyl)-4-((E)-3-hydroxyprop-1-en-1-yl)hepta-1,6-diene-3,5-dione |
| 316 | 96 | 3,5-bis((E)-3,4-dimethoxystyryl)-4-methyl-1H-pyrazole |
| 317 | 97 | methyl 3-(3,5-bis((E)-3,4-dimethoxystyryl)-1H-pyrazol-4-yl)acrylate |
| 318 | 98 | ethyl 3-(3,5-bis((E)-3,4-dimethoxystyryl)-1H-pyrazol-4-yl)acrylate |
| 319 | 99 | 3,5-bis((E)-3,4-dimethoxystyryl)-4-methylisoxazole |
| 320 | 100 | ethyl 3-(3,5-bis((E)-3,4-dimethoxystyryl)isoxazol-4-yl)acrylate |
| 321 | 106 | (1E,6E)-1,7-bis(3,4-dimethoxyphenyl)-4,4-dimethylhepta-1,6-diene-3,5-dione |
| 322 | 107 | (2E,2'E)-1,1'-(cyclohexane-1,1-diyl)bis(3-(3,4-dimethoxyphenyl)prop-2-en-1-one) |
| 323 | 109 | (1E,6E)-4-(4-hydroxy-3-methoxybenzyl)-1,7-bis(4-hydroxy-3-methoxyphenyl)hepta-1,6-diene-3,5-dione |
| 324 | 110 | (1E,6E)-1,7-bis(4-hydroxy-3-methoxyphenyl)-4-(2-(4-methoxyphenyl)hydrazineylidene)hepta-1,6-diene-3,5-dione |
| 325 | 111 | (1E,6E)-1,7-bis(4-hydroxy-3-methoxyphenyl)-4-(2-phenylhydrazineylidene)hepta-1,6-diene-3,5-dione |
| 326 | 112 | (1E,6E)-1,7-bis(4-hydroxy-3-methoxyphenyl)-4-(2-(p-tolyl)hydrazineylidene)hepta-1,6-diene-3,5-dione |
| 327 | 113 | (1E,6E)-4-(2-(4-chlorophenyl)hydrazineylidene)-1,7-bis(4-hydroxy-3-methoxyphenyl)hepta-1,6-diene-3,5-dione |
| 328 | 114 | (1E,6E)-1,7-bis(4-hydroxy-3-methoxyphenyl)-4-(2-(4-nitrophenyl)hydrazineylidene)hepta-1,6-diene-3,5-dione |
| 329 | 115 | 4,4'-((1E,1'E)-(1-hydrazineyl-4,5-dihydro-1H-pyrazole-3,5-diyl)bis(ethene-2,1-diyl))bis(2-methoxyphenol) |
| 330 | 116 | 4,4'-(((2-(4-nitrophenyl)hydrazineylidene)methylene)bis(4,5-dihydro-1H-pyrazole-3,5-diyl))bis(2-methoxyphenol) |
| 331 | 126 | ((1E,6E)-4-((1H-benzo[d]imidazol-2-yl)glycyl)-3,5-dioxohepta-1,6-diene-1,7-diyl)bis(2-ethoxy-4,1-phenylene) bis(2-((1H-benzo[d]imidazol-2-yl)amino)acetate) |
| 332 | 127 | ((1E,6E)-4-((5-methyl-1,3,4-thiadiazol-2-yl)glycyl)-3,5-dioxohepta-1,6-diene-1,7-diyl)bis(2-ethoxy-4,1-phenylene) bis(2-((5-methyl-1,3,4-thiadiazol-2-yl)amino)acetate) |
| 333 | 128 | 4-((1E,6E)-4-(3-(((1S,3R,5S)-adamantan-1-yl)amino)propanoyl)-7-(4-((3-(((1S,3R,5S)-adamantan-1-yl)amino)propanoyl)oxy)-3-methoxyphenyl)-3,5-dioxohepta-1,6-dien-1-yl)-2-methoxyphenyl 3-(((3s,5s,7s)-adamantan-1-yl)amino)propanoate |
| 334 | 129 | ((1E,6E)-4-(3-((5-methyl-1,3,4-thiadiazol-2-yl)amino)propanoyl)-3,5-dioxohepta-1,6-diene-1,7-diyl)bis(2-methoxy-4,1-phenylene) bis(3-((5-methyl-1,3,4-thiadiazol-2-yl)amino)propanoate) |
| 335 | 131 | (1E,6E)-4-((E)-4-hydroxy-3-methoxybenzylidene)-1-(4-hydroxy-3-methoxyphenyl)-7-(2,6,6-trimethylcyclohex-1-en-1-yl)hepta-1,6-diene-3,5-dione |
| 336 | 132 | (1E,4E,6E)-4-((9-ethyl-9H-carbazol-2-yl)methylene)-1-(4-hydroxy-3-methoxyphenyl)-7-(2,6,6-trimethylcyclohex-1-en-1-yl)hepta-1,6-diene-3,5-dione |
| 337 | 139 | (1E,6E)-4-(3,4-difluorobenzylidene)-1,7-bis(4-hydroxy-3-methoxyphenyl)hepta-1,6-diene-3,5-dione |
| 338 | 140 | 4,4'-((1E,3E,5E,6E)-4-(3,4-difluorobenzylidene)-3,5-bis((3,4-difluorophenyl)imino)hepta-1,6-diene-1,7-diyl)bis(2-methoxyphenol) |
| 339 | 142 | (2E,2'E)-2,2'-((1E,6E)-4-(3,4-difluorobenzylidene)-1,7-bis(4-hydroxy-3-methoxyphenyl)hepta-1,6-diene-3,5-diylidene)bis(hydrazine-1-carboxamide) |
| 340 | 143 | (2E,2'E)-2,2'-((1E,6E)-4-(3,4-difluorobenzylidene)-1,7-bis(4-hydroxy-3-methoxyphenyl)hepta-1,6-diene-3,5-diylidene)bis(hydrazine-1-carbothioamide) |
| 341 | 713 | (2E,5E)-2,5-bis((5-methylthiophen-2-yl)methylene)cyclopentan-1-one |
| 342 | 155 | (1E,4E)-1,5-di(pyridin-2-yl)penta-1,4-dien-3-one |
| 343 | 156 | (1E,4E)-1,5-bis(3,5-difluoropyridin-2-yl)penta-1,4-dien-3-one |
| 344 | 159 | (1E,4E)-1-(4-hydroxy-3,5-dimethoxyphenyl)-5-(3,4,5-tris(methylperoxy)phenyl)penta-1,4-dien-3-one |
| 345 | 160 | (1E,4E)-1-(4-(1-ethoxyethoxy)-3,5-dimethoxyphenyl)-5-(3-methoxy-4,5-bis(methylperoxy)phenyl)penta-1,4-dien-3-one |
| 346 | 162 | (1E,4E)-1-(3-(1-ethoxyethoxy)phenyl)-5-(3-methoxy-4,5-bis(methylperoxy)phenyl)penta-1,4-dien-3-one |
| 347 | 163 | (1E,4E)-1-(3-hydroxyphenyl)-5-(3-methoxy-4,5-bis(methylperoxy)phenyl)penta-1,4-dien-3-one |
| 348 | 166 | 1-benzyl-3,5-bis((E)-4-ethoxybenzylidene)piperidin-4-one |
| 349 | 167 | 1-benzyl-3,5-bis((E)-3-hydroxybenzylidene)piperidin-4-one |
| 350 | 170 | (1E,4E)-1,5-bis(4-hydroxy-3,5-dimethoxyphenyl)penta-1,4-dien-3-one |
| 351 | 172 | (1E,4E)-1,5-bis(3,5-dimethoxy-4-(methoxymethoxy)phenyl)penta-1,4-dien-3-one |
| 352 | 180 | (E)-6-(3,4-dihydroxyphenyl)hex-5-ene-2,4-dione |
| 353 | 181 | (E)-6-(4-hydroxy-3-methoxyphenyl)hex-5-ene-2,4-dione |
| 354 | 182 | (E)-6-(4-hydroxyphenyl)hex-5-ene-2,4-dione |
| 355 | 183 | (E)-6-(3,4,5-trihydroxyphenyl)hex-5-ene-2,4-dione |
| 356 | 184 | (E)-6-(3,4-dihydroxy-5-methoxyphenyl)hex-5-ene-2,4-dione |
| 357 | 185 | (E)-6-(4-hydroxy-3,5-dimethoxyphenyl)hex-5-ene-2,4-dione |
| 358 | 186 | (3E,5E)-3,5-bis((E)-3-(4-hydroxy-3-methoxy-5-methylphenyl)allylidene)-1-methylpiperidin-4-one |
| 359 | E21CH | (4Z,6E)-5-hydroxy-7-(4-hydroxy-3-methoxyphenyl)hepta-4,6-dien-3-one |
| 360 | Q012095H | (1E,4Z,6E)-1,7-bis(5-((2-(dimethylamino)ethyl)thio)thiophen-2-yl)-5-hydroxyhepta-1,4,6-trien-3-one |
| 361 | Q012138 | (S)-(4-((3-(benzo[b]thiophen-2-yl)-1-ethoxy-1-oxopropan-2-yl)carbamoyl)phenyl)methanaminium |
| 362 | Q012138_1 | (S)-4-((3-(benzo[b]thiophen-2-yl)-1-ethoxy-1-oxopropan-2-yl)carbamoyl)-N-methylbenzenaminium |
| 363 | Q012169AT | N-ethyl-5-hydroxy-2-phenoxybenzamide |
| 364 | BJC4 | (1E,6E)-1,7-bis(4-methoxy-3-nitrophenyl)hepta-1,6-diene-3,5-dione |
| 365 | BJC5 | (1E,6E)-1,7-bis(4-hydroxy-3-methoxy-5-nitrophenyl)hepta-1,6-diene-3,5-dione |
| 366 | CHC1 | (1E,6E)-1,7-bis(3-methoxyphenyl)hepta-1,6-diene-3,5-dione |
| 367 | CHC2 | (1E,6E)-1,7-bis(3,4,5-trimethoxyphenyl)hepta-1,6-diene-3,5-dione |
| 368 | CHC3 | (1E,6E)-1,7-bis(3,5-dimethoxyphenyl)hepta-1,6-diene-3,5-dione |
| 369 | CHC5 | (1E,6E)-1,7-bis(4-methoxyphenyl)hepta-1,6-diene-3,5-dione |
| 370 | CHC7 | (1E,6E)-1,7-bis(4-hydroxy-3-nitrophenyl)hepta-1,6-diene-3,5-dione |
| 371 | CHC8 | (1E,6E)-1,7-bis(3-hydroxyphenyl)hepta-1,6-diene-3,5-dione |
| 372 | CHC9 | (1E,6E)-1,7-bis(3-nitrophenyl)hepta-1,6-diene-3,5-dione |
| 373 | CHC11 | (1E,6E)-1,7-bis(4-nitrophenyl)hepta-1,6-diene-3,5-dione |
| 374 | A1 | 3,5-bis((E)-2,4,5-trimethoxybenzylidene)piperidin-4-one |
| 375 | A2 | 3,5-bis((E)-3,4,5-trimethoxybenzylidene)piperidin-4-one |
| 376 | A3 | 1-benzyl-3,5-bis((E)-2,4,5-trimethoxybenzylidene)piperidin-4-one |
| 377 | A4 | 1-benzyl-3,5-bis((E)-3,4,5-trimethoxybenzylidene)piperidin-4-one |
| 378 | 1BT | 1,8-cineole |
| 379 | 2BT | 3-methyl-2-methylene-3-[2-(1,3-dioxolan-2-yl)]bicyclo[2.2.2]octane |
| 380 | 3BT | Pumiliotoxin |
| 381 | 4BT | Tetrahydro-4b,4c,9b,9c-tetramethyl-2,7-d imethoxycyclobutaimethoxycyclobuta[1,2-a:3,4-a']diindene-5,10-dione |
| 382 | 5BT | 2-methoxy-1-phenylcyclohexylamine |
| 383 | 6BT | alpha-selinene |
| 384 | 7BT | bicyclo[2.2.1]hept-5-en-2-one |
| 385 | 8BT | Oxauracil |
| 386 | 9BT | ethyl N-[(ethoxycarbonyl)methyl]-9-azabicyclo[3.3.1]nonane-4-carboxylate |
| 387 | 10BT | 19-Methylenepentaspiro [3.0.2.0.3.0.2.0.3.1] nonadecane |
| 388 | 11BT | Tricyclo[3.2.1.0(2,4)]octane, 3,3-dimethyl-8-methylene-, (1à,2à,4à,5à) |
| 389 | 12BT | Spathulenol |
| 390 | 13BT | Camphor |
| 391 | 14BT | (1S)-(+)-2,2-dimethyl-6-methylene-1-cyclohexanemethanol |
| 392 | 15BT | ethyl 2,3,3a,6,7,7a-hexahydro-3,3-dimethyl-3a,6-epoxy-1-oxoindene-7-carboxylate |
| 393 | 16BT | ethyl (3-isopropyl-2-oxo-5-phenyltetrahydrofuran-3-yl)carbamate |
| 394 | 17BT | 3-(2-Furyl)-3-methylindan-1-one |
| 395 | 18BT | Alpha citronellol |
| 396 | 19BT | Norambreinolid |
| 397 | 20BT | 4,4'-Bis[2-phenyl-3,6-dioxabicyclo[3.2.1]octan-7-one] |
| 398 | 21BT | Isoborneol |
| 399 | 22BT | Borneol |
| 400 | 23BT | Alpha-Terpineol |
| 401 | 24BT | Isoborneol acetate |
| 402 | 25BT | beta elemene |
| 403 | 26BT | caryophyllene |
| 404 | 27BT | Alpha Selinene |
| 405 | 28BT | alpha bulnesene |
| 406 | 29BT | Delta cadinene |
| 407 | 30BT | Caryophyllene oxide |
| 408 | 31BT | Gamma-Cadinene |
| 409 | 32BT | Rosifoliol |
| 410 | 33BT | Tropolone |
| 411 | 34BT | cis-alpha-Copaene-8-ol |
| 412 | 35BT | Beta Selinene |
| 413 | 36BT | Beta-guaiene |
| 414 | 37BT | Globulol |
| 415 | 38BT | Ledol |
| 416 | 39BT | ar-Turmerone |
| 417 | 40BT | Decahydro-1,1,7-trimethyl-4-methylene-,[1ar-(1a.alpha.,4a.alpha.,7.beta.,7a.beta.,7b.alpha |
| 418 | 41BT | 2,7 di methyl Oxepine |
| 419 | 42BT | Beta elemenone |
| 420 | 43BT | Occidentalol |
| 421 | 44BT | Cyclohexanol, 1,3,3-trimethyl-2-(3-methyl-2-methylene-3-butenylidene) |
| 422 | 45BT | 6-isopropenyl-4,8a-dimethyl-1,2,3,5,6,7,8,8a-octahydro-napthalene-2ol |
| 423 | 46BT | megastigmatrienone |
| 424 | 47BT | 4-dimethylamino-benzoic acid |
| 425 | 48BT | 4-(dimethylamino)-, 3,5-dimethyl-phenol |
| 426 | 49BT | 6-isopropylidene-bicyclo[3.1.0]hexane |
| 427 | 50BT | Bicycle[3.1.0]hexan-3one |
| 428 | 51BT | 6-methyl-2(1H)-pteridinone |
| 429 | 52BT | 1,4-dihydro-1,1,4,4-tetramethyl-2,3-Naphthalenedione |
| 430 | 53BT | Delta1(9)-2-Octalone |
| 431 | 54BT | alpha-elemol |
| 432 | 55BT | Epiglobulol |
| 433 | 56BT | viridiflorol |
| 434 | 57BT | Germacrene B |
| 435 | 58BT | beta-caryophyllene |
| 436 | 59BT | gamma-Elemene |
| 437 | 60BT | Gamma-curcumene |
| 438 | 61BT | ar-curcumene |
| 439 | 62BT | Terpinen-4-ol |
| 440 | 63BT | Estragole |
| 441 | 64BT | Endo-fenchyl acetate |
| 442 | 65BT | Bornyl acetate |
| 443 | 66BT | delta-Elemene |
| 444 | 67BT | beta-cubebene |
| 445 | 68BT | beta-elemene |
| 446 | 69BT | (Z,E)-alpha-Farnesene |
| 447 | 70BT | Camphene |
| 448 | 71BT | p-cymene |
| 449 | 72BT | (Z-)Beta-Ocimene |
| 450 | 73BT | Nonanone |
| 451 | 74BT | alpha-Terpinolene |
| 452 | 75BT | Linalool |
| 453 | 76BT | Endo-fenchol |
| 454 | 77BT | Methone |
| 455 | 78BT | Isomenthone |
| 456 | 79BT | bornylene |
| 457 | 80BT | ocimene |
| 458 | 81BT | curzerene |
| 459 | 82BT | curzerenone |
| 460 | 83BT | zingiberol |
| 461 | 84BT | (1S,6R,7R)-1-methyl-7-(3-oxobutyl)-4-(propan-2-ylidene)bicyclo[4.1.0]heptan-3-one |
| 462 | 85BT | (5E,9E)-3,6,10-trimethyl-4,7,8,11-tetrahydrocyclodeca[b]furan |
| 463 | 86BT | (5E,9E)-3,6,10-trimethyl-8,11-dihydrocyclodeca[b]furan-4(7H)-one |
| 464 | 87BT | (3E,6Z,10E)-10-(1-hydroxypropan-2-ylidene)-3,7-dimethylcyclodeca-3,6-dien-1-one |
| 465 | 88BT | (3aR,8R,9aR)-3a-hydroxy-1,5,8-trimethyl-4,7,8,9a-tetrahydronaphtho[2,1-b]furan-2,9(3aH,6H)-dione |
| 466 | 89BT | (3S,3aR,6S,8aR)-3,8-dimethyl-5-(propan-2-ylidene)-1,2,3,4,5,8a-hexahydro-6H-3a,6-epoxyazulen-6-ol |
| 467 | 90BT | (1S,3aR,4R,8aR)-1,4-dimethyl-7-(propan-2-ylidene)decahydroazulen-4-ol |
| 468 | 91BT | (1S,3aS,4S,8aR)-1,4-dihydroxy-1,4-dimethyl-7-(propan-2-ylidene)octahydroazulen-6(1H)-one |
| 469 | 92BT | 1-methyl-4-[(2R)-6-methylheptan-2-yl]benzene |
| 470 | 93BT | (3R,8aR)-5,8a-dimethyl-3-(propan-2-yl)-1,2,3,7,8,8a-hexahydronaphthalene |
| 471 | 94BT | (1r,4r)-4,7,7-trimethylbicyclo[2.2.1]heptan-1-ol |
| 472 | 95BT | (3R,3aS)-3,8a-dihydroxy-3,3a,8-trimethyl-5-(propan-2-ylidene)-2,3,3a,4,5,8a-hexahydroazulen-6(1H)-one |
| 473 | 96BT | (3R,3aR,6S,8aR)-3,8-dimethyl-5-(propan-2-ylidene)-1,2,3,4,5,8a-hexahydro-6H-3a,6-epoxyazulen-6-ol |
| 474 | 97BT | (1aS,4Z,10aR)-1a,5,9-trimethyl-1a,3,6,10a-tetrahydrooxireno[4,5]cyclodeca[1,2-b]furan-10(2H)-one |
| 475 | 98BT | (5Z,9Z)-3,6,10-trimethyl-4,7,8,11-tetrahydrocyclodeca[b]furan |
| 476 | 99BT | (3Z,7Z)-3,7-dimethyl-10-(propan-2-ylidene)cyclodeca-3,7-dien-1-one |
| 477 | 100BT | (1R,10S)-6,10-dimethyl-3-(propan-2-ylidene)-11-oxabicyclo[8.1.0]undec-6-en-4-one |
| 478 | 101BT | (5Z,9E)-3,6,10-trimethyl-8,11-dihydrocyclodeca[b]furan-4(7H)-one |
| 479 | 102BT | 6-ethenyl-3,6-dimethyl-5-(prop-1-en-2-yl)-6,7-dihydro-1-benzofuran-4(5H)-one |
| 480 | 103BT | (1R,6S,7S)-1-methyl-7-(3-oxobutyl)-4-(propan-2-ylidene)bicyclo[4.1.0]heptan-3-one |
| 481 | 78 | 2-((E)-4-hydroxy-3-methoxybenzylidene)-5-((E)-4-hydroxybenzylidene)cyclopentan-1-one |
| 482 | 21 | 4,4'-((1E,1'E)-(1H-pyrazole-3,5-diyl)bis(ethene-2,1-diyl))diphenol |
| 483 | 20 | 4-((E)-2-(5-((E)-4-hydroxystyryl)-1H-pyrazol-3-yl)vinyl)-2-methoxyphenol |
| 484 | 159 | 2^6^-hydroxy-1^5^,1^6^-dimethoxy-6-aza-1,2(1,3)-dibenzenacyclodecaphan-5-one |
| 485 | 58 | (1E,6E)-1,7-diphenylhepta-1,6-diene-3,5-dione |
| 486 | 122 | (1E,4Z,6E)-1,7-bis(4-fluorophenyl)-5-hydroxyhepta-1,4,6-trien-3-one |
| 487 | 123 | (1E,4Z,6E)-1,7-bis(3-fluorophenyl)-5-hydroxyhepta-1,4,6-trien-3-one |
| 488 | 124 | (1E,4Z,6E)-1,7-bis(2-fluorophenyl)-5-hydroxyhepta-1,4,6-trien-3-one |
| 489 | 125 | (1E,4Z,6E)-1,7-bis(3-fluoro-4-methoxyphenyl)-5-hydroxyhepta-1,4,6-trien-3-one |
| 490 | 126 | (1E,4Z,6E)-1,7-bis(4-fluoro-3-(trifluoromethyl)phenyl)-5-hydroxyhepta-1,4,6-trien-3-one |
| 491 | 127 | (1E,4Z,6E)-5-hydroxy-1,7-bis(4-(trifluoromethoxy)phenyl)hepta-1,4,6-trien-3-one |
| 492 | 140 | 2-bromo-1,3-bis(4-nitrophenyl)propane-1,3-dione |
| 493 | 141 | 1,3-bis(4-nitrophenyl)propane-1,3-dione |
| 494 | 142 | 2-benzoyl-1,4-diphenylbutane-1,4-dione |
| 495 | 143 | ((1E,3Z,6E)-3-hydroxy-5-oxohepta-1,3,6-triene-1,7-diyl)bis(2-methoxy-4,1-phenylene) diethaneperoxoate |
| 496 | 144 | (1E,4Z,6E)-1,7-bis(3,4-dihydroxyphenyl)-5-hydroxyhepta-1,4,6-trien-3-one |
| 497 | 145 | (1E,4Z,6E)-1,7-bis(3,4-dimethoxyphenyl)-5-hydroxy-4-methylhepta-1,4,6-trien-3-one |
| 498 | 180 | 1-(4-aminophenyl)-2-bromo-3-(4-methoxyphenyl)propane-1,3-dione |
| 499 | 163 | 1,3-di(pyridin-3-yl)-2-(pyridin-3-ylmethylene)propane-1,3-dione |
| 500 | 153 | 2^6^-hydroxy-1^5^,1^6^-dimethoxy-1,2(1,3)-dibenzenacyclononaphan-6-one |
| 501 | 183 | 4-methoxybenzoic 4-nitrobenzoic anhydride compound with 1-(4-methoxyphenyl)-2-methylene-3-(3-nitrosophenyl)propane-1,3-dione (1:1) |
| 502 | 184 | 1-(3-(1,3-dioxo-1,3-diphenylpropan-2-yl)phenyl)-2-methyl-3-phenylpropane-1,3-dione |
| 503 | 185 | 2-(furan-2-ylmethyl)-1,3-diphenylpropane-1,3-dione |
| 504 | 131 | 1^4^,2^6^-dihydroxy-1^5^,1^6^-dimethoxy-1,2(1,3)-dibenzenacyclononaphan-5-one |
| 505 | 132 | 2^6^-hydroxy-1^4^,1^5^,1^6^-trimethoxy-1,2(1,3)-dibenzenacyclononaphan-5-one |
| 506 | 133 | 1^4^,1^5^,1^6^,2^6^-tetramethoxy-1,2(1,3)-dibenzenacyclononaphan-5-one |
| 507 | 134 | 1^5^,1^6^-dimethoxy-5-oxo-1,2(1,3)-dibenzenacyclononaphane-14,26-diyl diacetate |
| 508 | 356 | (E)-1-(3-aminophenyl)-3-(4-hydroxy-3-methoxyphenyl)prop-2-en-1-one |
| 509 | 357 | (E)-N-(3-(3-(4-hydroxy-3-methoxyphenyl)acryloyl)phenyl)acetamide |
| 510 | 358 | (E)-N-(3-(3-(4-hydroxy-3-methoxyphenyl)acryloyl)phenyl)butyramide |
| 511 | 359 | (E)-3-(4-hydroxy-3-methoxyphenyl)-1-(3-(isopropoxyamino)phenyl)prop-2-en-1-one |
| 512 | 360 | ethyl (E)-2-((3-(3-(4-hydroxy-3-methoxyphenyl)acryloyl)phenyl)amino)-2-oxoacetate |
| 513 | 361 | methyl (E)-4-((3-(3-(4-hydroxy-3-methoxyphenyl)acryloyl)phenyl)amino)-4-oxobutanoate |
| 514 | 362 | (E)-3-(4-hydroxy-3-methoxyphenyl)-1-(3-(phenoxyamino)phenyl)prop-2-en-1-one |
| 515 | 363 | (E)-1-(3-((4-chlorophenoxy)amino)phenyl)-3-(4-hydroxy-3-methoxyphenyl)prop-2-en-1-one |
| 516 | 364 | (E)-1-(3-((2,4-dichlorophenoxy)amino)phenyl)-3-(4-hydroxy-3-methoxyphenyl)prop-2-en-1-one |
| 517 | 365 | (E)-1-(3-((2,6-dichlorophenoxy)amino)phenyl)-3-(4-hydroxy-3-methoxyphenyl)prop-2-en-1-one |
| 518 | 366 | (E)-1-(3-((3,4-dichlorophenoxy)amino)phenyl)-3-(4-hydroxy-3-methoxyphenyl)prop-2-en-1-one |
| 519 | 367 | (E)-1-(3-((3,5-dichlorophenoxy)amino)phenyl)-3-(4-hydroxy-3-methoxyphenyl)prop-2-en-1-one |
| 520 | 370 | (1E,6E)-1,7-bis(2-hydroxy-5-methoxyphenyl)hepta-1,6-diene-3,5-dione |
| 521 | 371 | (1E,6E)-1-(2-hydroxyphenyl)-7-(3-hydroxyphenyl)hepta-1,6-diene-3,5-dione |
| 522 | 372 | (1E,6E)-1,7-bis(4-(dimethylamino)phenyl)hepta-1,6-diene-3,5-dione |
| 523 | 373 | (1E,6E)-1-(4-hydroxy-3,5-dimethoxyphenyl)-7-(4-hydroxy-3-methoxyphenyl)hepta-1,6-diene-3,5-dione |
| 524 | 374 | (1E,6E)-1-(3-bromo-4-hydroxy-5-methoxyphenyl)-7-(3-bromo-4-hydroxyphenyl)hepta-1,6-diene-3,5-dione |
| 525 | 375 | (1E,6E)-1,7-bis(3-(tert-butyl)-4-hydroxyphenyl)hepta-1,6-diene-3,5-dione |
| 526 | 376 | (1E,6E)-1,7-bis(3,5-di-tert-butyl-4-hydroxyphenyl)hepta-1,6-diene-3,5-dione |
| 527 | 377 | (1E,6E)-1,7-bis(3,4-bis(benzyloxy)-5-methoxyphenyl)hepta-1,6-diene-3,5-dione |
| 528 | 378 | dimethyl 5,5'-((1E,6E)-3,5-dioxohepta-1,6-diene-1,7-diyl)bis(2-hydroxybenzoate) |
| 529 | 154 | 2^6^-hydroxy-1^5^,1^6^-dimethoxy-14-methyl-1,2(1,3)-dibenzenacyclononaphan-6-one |
| 530 | 380 | (1E,6E)-1-(3,4-dihydroxyphenyl)-7-(4-hydroxy-3-methoxyphenyl)hepta-1,6-diene-3,5-dione |
| 531 | 381 | (1E,6E)-1,7-bis(2,5-dihydroxyphenyl)hepta-1,6-diene-3,5-dione |
| 532 | 382 | (1E,6E)-1,7-bis(3,4-dihydroxy-5-methoxyphenyl)hepta-1,6-diene-3,5-dione |
| 533 | 383 | (1E,6E)-1-(3,4-dihydroxy-5-methoxyphenyl)-7-(3,4-dihydroxyphenyl)hepta-1,6-diene-3,5-dione |
| 534 | 384 | (1E,6E)-1-(3,4-dihydroxyphenyl)-7-(3,4,5-trihydroxyphenyl)hepta-1,6-diene-3,5-dione |
| 535 | 385 | (1E,6E)-1,7-bis(3,4,5-trihydroxyphenyl)hepta-1,6-diene-3,5-dione |
| 536 | 386 | 5,5'-((1E,6E)-3,5-dioxohepta-1,6-diene-1,7-diyl)bis(2-hydroxybenzoic acid) |

*Grey highlights- 30 compounds selected from initial screening as mentioned in Table 1, 2, 3(A-B); BT-Black turmeric *C. caesia. Roxb.* compounds
